# A Strategy to Select Macrocyclic Peptides Featuring Asymmetric Molecular Scaffolds as Cyclization Units by Phage Display

**DOI:** 10.1021/jacs.1c12822

**Published:** 2022-02-16

**Authors:** Titia Rixt Oppewal, Ivar D. Jansen, Johan Hekelaar, Clemens Mayer

**Affiliations:** Stratingh Institute for Chemistry, University of Groningen, Nijenborgh 4, Groningen 9474 AG, The Netherlands

## Abstract

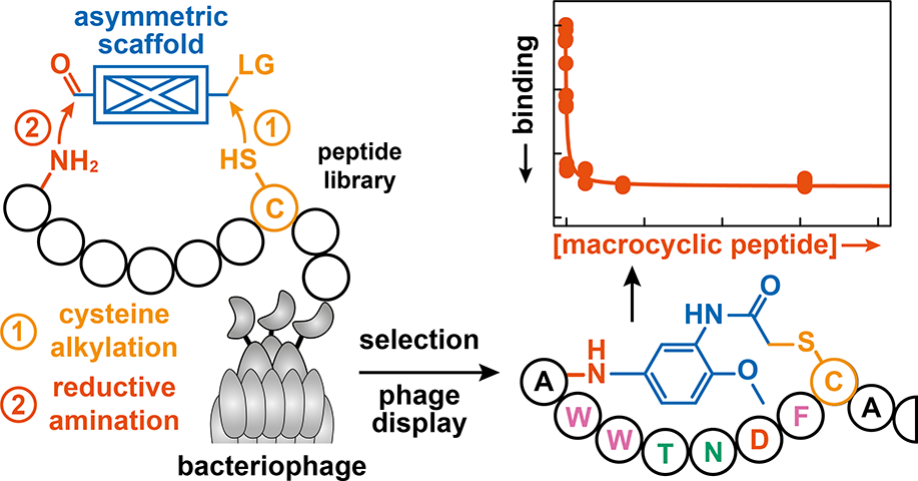

Macrocyclic peptides (MPs) have positioned themselves as a privileged class of compounds for the discovery of therapeutics and development of chemical probes. Aided by the development of powerful selection strategies, high-affinity binders against biomolecular targets can readily be elicited from massive, genetically encoded libraries by affinity selection. For example, in phage display, MPs are accessed on the surface of whole bacteriophages via disulfide formation, the use of (symmetric) crosslinkers, or the incorporation of non-canonical amino acids. To facilitate a straightforward cyclization of linear precursors with asymmetric molecular scaffolds, which are often found at the core of naturally occurring MPs, we report an efficient two-step strategy to access MPs via the programmed modification of a unique cysteine residue and an N-terminal amine. We demonstrate that this approach yields MPs featuring asymmetric cyclization units from both synthetic peptides and when linear precursors are appended onto a phage-coat protein. Finally, we showcase that our cyclization strategy is compatible with traditional phage-display protocols and enables the selection of MP binders against a model target protein from naïve libraries. By enabling the incorporation of non-peptidic moieties that (1) can serve as cyclization units, (2) provide interactions for binding, and/or (3) tailor pharmacological properties, our head-to-side-chain cyclization strategy provides access to a currently under-explored chemical space for the development of chemical probes and therapeutics.

## Introduction

**Figure 1:**
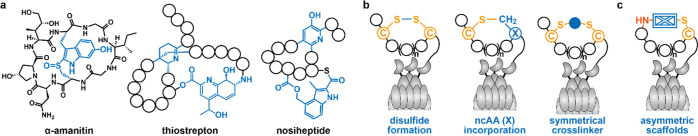
Creating natural-product-like MPs that feature non-peptidic cyclization units on whole bacteriophages. (a) Structures or representations of naturally occurring MPs, with (non-peptidic) asymmetric cyclization units highlighted in blue. (b) Schematic overview of peptide-cyclization strategies compatible with phage display. Molecules that crosslink two or more of the same amino acids must be symmetric to avoid the formation of stereo- or regio-isomers. (c) The programmed modification of two distinct functional groups, here a unique thiol and an N-terminal amine, alleviates symmetry constraints for cyclization units and enables the incorporation of asymmetric molecular scaffolds.

Macrocyclic peptides (MPs) hold great promise for the discovery of lead compounds in drug discovery efforts and the development of chemical probes to interrogate biological functions.^1−5^ When compared to their linear counterparts, MPs combine several features that make them attractive for potential applications in the clinic and in research labs:^6−9^ (1) cyclization of linear peptides restricts their conformational flexibility and reduces the entropic penalty upon binding to a biological target, (2) MPs cannot only target well-defined binding pockets but also disrupt protein–protein interfaces with high affinities and selectivities, and (3) short MPs (<15 amino acids) elicit a low immune response and display good stability against protease degradation. The presence of a plethora of MPs in nature, where their biosynthesis bestows a competitive advantage to producing organisms, further attests on the versatile nature of MPs as bioactive compounds.^10,11^ For example, thiostrepton and nosiheptide are produced by bacteria to keep rivaling bacterial populations in check, while the biosynthesis of α-amanitin in the death cap mushroom renders its consumption toxic (Figure 1a).^12−14^

Critically, MPs found in nature are the result of evolutionary algorithms that finetuned both their amino acid sequences and the post-translational processes for the introduction of non-peptidic moieties as cyclization units. Mimicking such a *chemogenetic* optimization process in the laboratory is desirable but can be challenging as synthetic approaches for peptide diversification and/or macrocyclization are often not compatible with the biological strategies used to select binders from genetically encodable peptide libraries.^15−19^ However, a seamless integration of chemical cyclization strategies with biological selection approaches is critical. By providing a rapid means to identify MP binders from vast libraries with 10^8^ to 10^13^ members, biological selections circumvent the laborious task of synthesizing and assessing MPs one by one.^20−22^

Among available in vitro and in vivo selection strategies, phage display has proven a robust platform for the identification of binders as randomized linear peptides can readily be appended onto phage-coat proteins.^23,24^ Strategies that enable the selection of MPs achieve cyclization via the formation of disulfides,^25−28^ the introduction of non-canonical amino acids (ncAAs) with uniquely reactive handles that trigger the spontaneous macrocyclization,^29−31^ or the action of small-molecule cross linkers (Figure 1b).^32^ Phage-compatible approaches for the latter have been pioneered by the Heinis and Derda groups, who created MP libraries by the reaction of two or three cysteine side chains with symmetric lynchpin molecules that feature weak electrophilic groups (e.g., di-/tri-bromomethylbenzenes or bromo-/chloro-acetamides).^33−37^ In these approaches, the use of crosslinkers with two- or threefold symmetry is critical to prevent the unwanted formation of regioisomers and/or diastereomers.^38−40^ Further refining these strategies to enable the straightforward incorporation of asymmetric molecular scaffolds is desirable^41,42^ as these moieties are often found in natural MPs (Figure 1a), where they are critical for fine-tuning pharmacological properties and can improve binding to protein targets.

To provide access to such natural-product-like MPs, we describe a two-step strategy to select MPs featuring asymmetric molecular scaffolds as cyclization units by phage display (Figure 1c). Specifically, we make use of a selective head-to-side-chain cyclization following an initial cysteine alkylation step with diverse molecular scaffolds. Notably, the resulting natural-product-like MPs provide access to an under-explored chemical space from which new binders against biological targets can be selected.

## Results and Discussion

### Two-Step Head-to-Side-Chain Peptide Cyclization Strategy

Accessing well-defined MPs through the incorporation of asymmetric molecular scaffolds instead of symmetric crosslinkers necessitates the programmed modification of two distinct functional groups with orthogonal reactivity in a peptide or protein substrate. Besides making use of a uniquely reactive cysteine thiol, such approaches typically rely on the incorporation of ncAAs to install an orthogonal handle for a second modification.^43,44^ Most notably, the Fasan group has accessed conformationally constrained organo–peptide hybrids in bacteria via such a tandem chemoselective reaction between synthetic molecules and genetically encoded peptides.^45−48^

**Figure 2:**
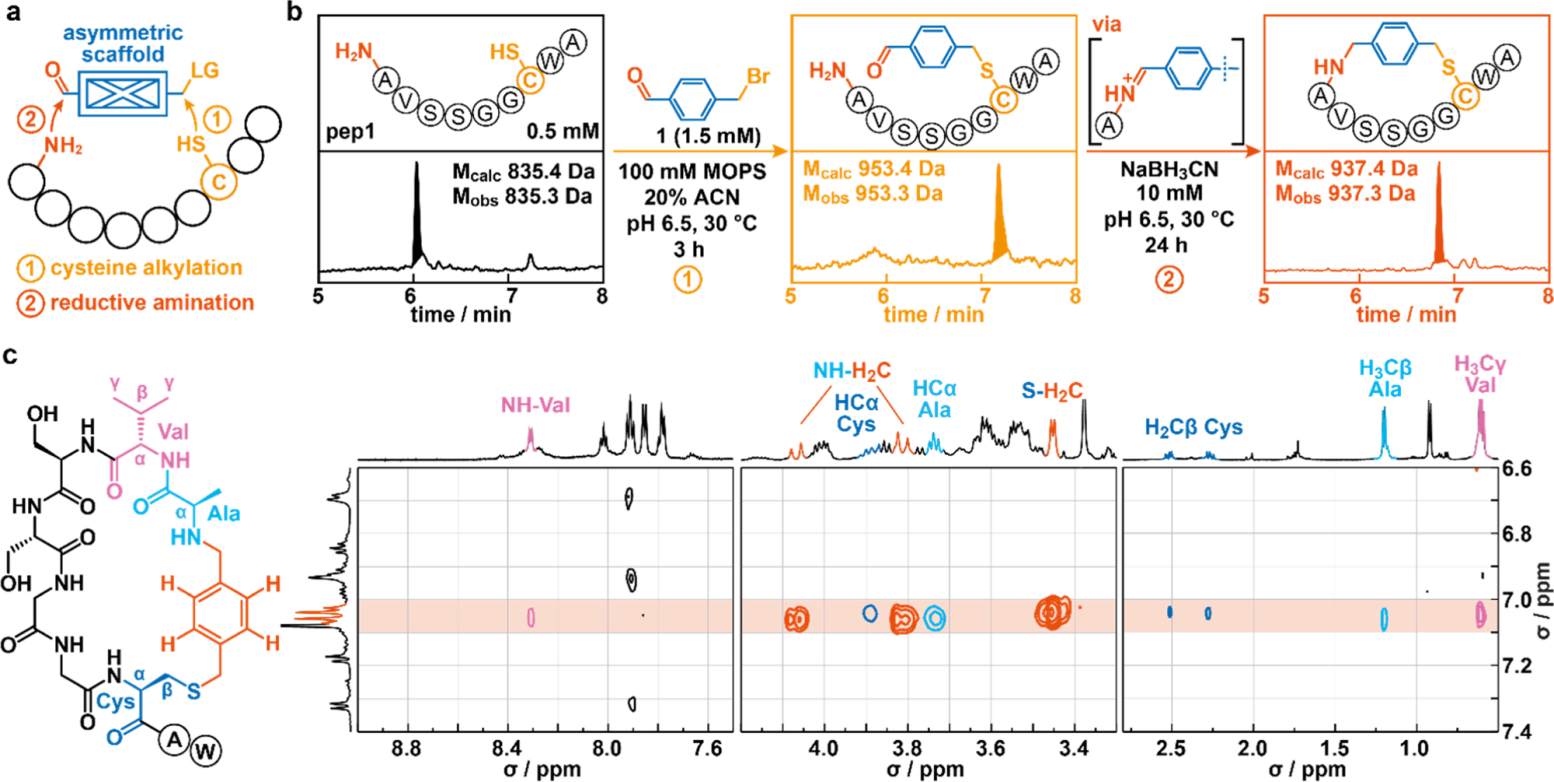
Two-step, head-to-side-chain peptide cyclization strategy gives rise to a model MP in high selectivity. (a) Schematic representation of the proposed two-step, head-to-side-chain cyclization strategy. Cyclic peptides are obtained via (1) the alkylation of a unique cysteine residue and the subsequent reductive amination of a transiently formed iminium ion. (b) Reaction conditions and representative examples of UPLC-MS chromatograms of synthetic pep1 (left) and crude reaction mixtures that were obtained following the modification (middle) and cyclization (right) of pep1 with 4-(bromomethyl)benzaldehyde. Total ion counts (TICs) are depicted with masses found for the highlighted, major species inserted. (c) Structure of the obtained cyclic product from the reaction of pep1 with **1** as well as excerpts from a 2D NOESY spectrum are shown (see Figure S3 for full spectrum). NOEs between the phenylic protons and amino acid residues are highlighted. Color code: phenylic protons = red, cysteine protons = dark blue, alanine protons = light blue, valine protons = magenta. Note that excerpts of the spectrum have been scaled independently to highlight the weak NOEs observed between phenylic protons and NH-Val as well as H_2_Cβ-Cys.

To enable the formation and selection of such organo-peptide hybrids on whole bacteriophages, we reasoned that peptides and/or proteins with a unique cysteine residue and a nearby N-terminal amine should readily undergo cyclization in the presence of bifunctional cyclization units featuring a good leaving group and an (aromatic) aldehyde (Figure 2a). Specifically, the initial cysteine-alkylation step should bring the aldehyde moiety of the cyclization unit in close proximity to the N-terminal amine. Then, condensation of these two moieties would result in the transient formation of an iminium ion, which can be selectively and irreversibly reduced by NaBH_3_CN to install an amine linkage. Notably, both cysteine alkylation and reductive amination typically proceed under mild conditions and are therefore expected to be compatible with whole bacteriophages.^43,44^

### Proof-of-Concept Cyclization on a Model Peptide

To test this approach, we first synthesized a model peptide (**H**_**2**_**N**-AVSSGG**C**WA-CONH_2_, pep1) featuring a flexible sequence between the N-terminal alanine and a unique cysteine residue as well as a tryptophan residue to allow for accurate quantification (Figure 2b). Next, we added commercially available 4-(bromomethyl)benzaldehyde (**1**, 1.5 mM) as a model bifunctional cyclization unit to crude pep1 (0.5 mM) at pH 6.5 and followed the reaction progress by ultraperformance liquid chromatography–mass spectrometry (UPLC-MS). As anticipated, we observed full conversion to the cysteine-modified peptide over a period of 3 h (Figure 2b). The following addition of NaBH_3_CN (three additions, final concentration = 10 mM) to the reaction mixture resulted in the smooth conversion (>90%) of the intermediate over 24 h to a new species. Critically, the mass observed for the reaction product (−16 Da when compared to the alkylated peptide) is consistent with the cyclization occurring via the envisioned reductive amination.

To confirm the nature and connectivity of the resulting product, we recorded one-dimensional (1D) and two-dimensional (2D) NMR spectra for pep1 as well as for crude products obtained after cysteine modification and the subsequent reduction step (see the Supporting Information for details). Several indirect and direct observations confirmed the successful cyclization of pep1 with the correct head-to-side-chain connectivity (Supporting Information Figures S1–S2). Among others, we noted the splitting of protons for several methylene groups of amino acid main and side chains, which were particularly notable for the β-protons of cysteine. Moreover, in comparison to the modified intermediate, we observed the disappearance of the benzaldehyde proton in the reduced product, which was accompanied with a drastic shift of most of the amide protons. These observations are consistent with the formation of a cyclic product, which would significantly influence the environment of protons in both methylene groups and the amide backbone. Critically, when performing nuclear Overhauser effect spectroscopy (NOESY) on the sample containing the crude cyclized product, we identified several interactions between **1** and the synthetic peptide that are consistent with peptide cyclization (Figure 2c). Specifically, nuclear Overhauser effects (NOEs) were not only found between aromatic protons and those of cysteine—the site of initial modification—but also with protons from the N-terminal alanine and valine residues. Similarly, we observed NOEs between the benzylic protons of the cyclization unit and those of cysteine and the N-terminal alanine residue (Figure S3). Combined, these experiments attest that the envisioned two-step strategy results in the formation of MPs following the selective, reductive amination of a transiently formed iminium ion.

### Generality of the Two-Step Cyclization Strategy

**Figure 3:**
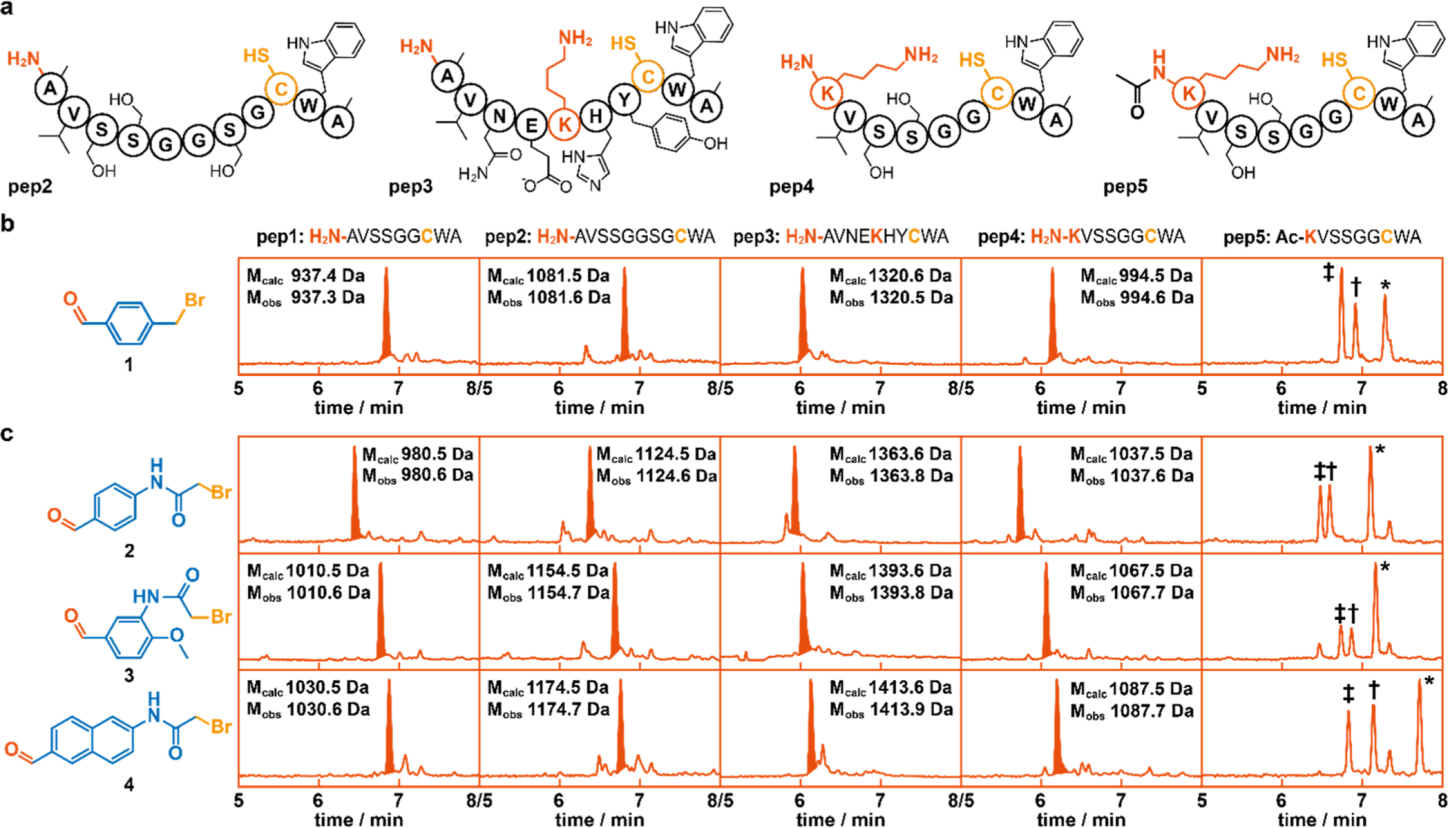
Head-to-side-chain cyclization proceeds with diverse peptides and bifunctional cyclization units. (a) Schematic representation of model peptides used to investigate the generality of our two-step cyclization strategy: pep1–4 vary in length and composition. The N-terminal and ε-amines from lysine residues are highlighted in red. Note that for pep5, the N-terminal amine functionality is blocked by acetylation. (b,c) Representative UPLC-MS chromatograms of crude reaction mixtures obtained for pep1–5 following cyclization with cyclization units **1–4**. TICs are depicted with masses found for the major species inserted. For pep5, the species formed are as follows: alkylated linear peptides with the aldehyde intact (*) or reduced to an alcohol (†) as well as some cyclization via the lysine side-chain amine (‡). UPLC chromatograms of linear precursors and peptides modified with **1–4** are displayed in Figures S4 and S6–S9.

To investigate the generality of this cyclization strategy, we prepared four additional model peptides (pep2–5, Figure 3a). Besides varying the ring sizes that can be formed upon cyclization (pep2 and pep3), we assessed the potential cross-reactivity of amino acid side chains in the modification and reductive amination step, with a particular focus on ε-amines from lysine residues. As such, pep3 and pep4 feature potentially competing internal and N-terminal lysine residues, respectively, while pep5 is an analogue of pep4 in which the N-terminal amine is blocked by acetylation. Modification and cyclization reactions were performed in the presence of **1** under analogous conditions to those employed for pep1, and UPLC-MS analyses confirmed the smooth conversion to the desired cyclic peptide for pep2–4 (Figures 3b and S4). Subjecting the crude reaction product of pep4 to 1D and 2D NMR analysis further indicated the formation of an MP via the N-terminal amine rather than that of the lysine side chain (Figure S5). Specifically, we observed NOEs between protons of the cyclization unit and the α- and β-carbons of the N-terminal lysine but not with protons from the γ-ε positions. Consistent with the unique reactivity of the N-terminus (p*K*_a_ ∼ 8 vs 10.5 for ε-amines of lysine side chains), upon its acetylation, the reduction step proceeded sluggishly. Specifically, pep5 afforded a mixture of alkylated linear peptides with the aldehyde intact or reduced to the corresponding alcohol, as well as some MP resulting from cyclization via the lysine side-chain amine (Figure 3B). This selectivity of the N-terminal over ε-amines of lysine residues is consistent with the increased nucleophilicity of the N-terminus under neutral conditions, which has been previously exploited for the selective N-terminal modification of peptides and proteins.^49−51^

One notable advantage of the programmed modification of two functional groups in a peptide is the ability for the incorporation of asymmetric molecular scaffolds in order to avoid the formation of regioisomers that are observed when using traditional crosslinking strategies.^38−40^ To showcase this aspect, we synthesized three bifunctional, asymmetric cyclization units (**2–4**, see the Supporting Information for details)^52−54^ that in addition to the benzaldehyde functionality featured an electrophilic bromoacetamide moiety. When subjecting pep1–4 to our cyclization protocol, all three linkers underwent efficient conversion to the corresponding MPs (Figures 3c and S6–S8). Once again, only pep5, for which the N-terminal amine is blocked by acetylation, gave rise to the same mixture of products observed with **1** (Figure S9). Together, these results demonstrate the generality of our strategy for the cyclization of synthetic peptides with a set of bifunctional cyclization units. Notably, the efficient formation of MPs when following this two-step protocol proved selective for the N-terminal amine and independent of the amino acid sequence and the cyclization unit used.

### Cyclization of Peptides Appended to a Phage-Coat Protein

**Figure 4:**
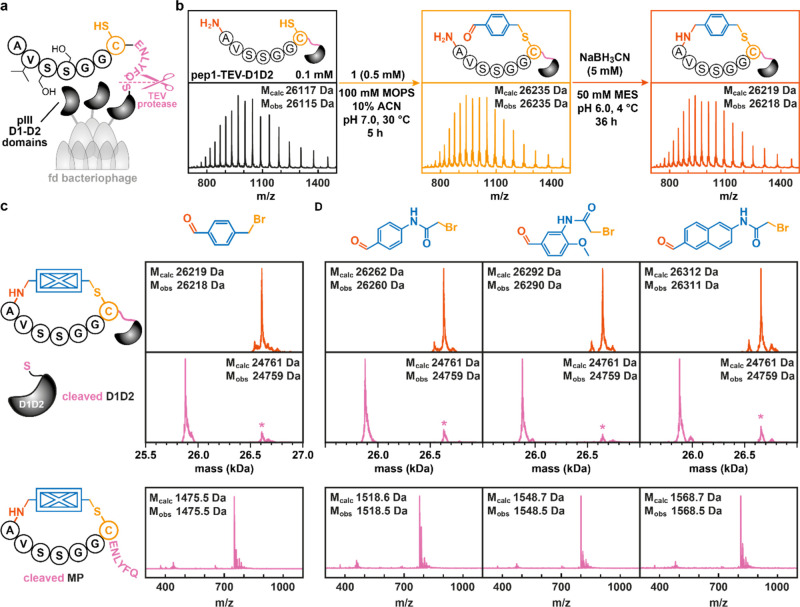
Head-to-side-chain cyclization of peptides appended onto a phage-coat protein. (a) Schematic representation of the soluble D1D2-domain of the fd bacteriophage. In this work, a peptide featuring a unique cysteine residue, followed by the recognition site for the TEV protease, was installed. The latter enables the cleavage of MPs following the two-step cyclization approach. (b) Reaction conditions employed for the cyclization of peptides on soluble D1D2 domains as well as representative raw mass spectra obtained for the purified protein and crude reaction mixtures of proteins following modification and cyclization. (c,d) Deconvoluted mass spectra of protein fragments before (top) and after TEV cleavage (middle) following the cyclization of pep-TEV-D1D2 with bifunctional cyclization units **1**–**4**. Peaks labeled with a * denote pep-TEV-D1D2 species that have cyclized but did not undergo cleavage. For raw mass spectra of protein fragments before and after TEV cleavage, see Figures S10 and S12. Mass spectra obtained for the cleaved cyclic peptides (+2 species are shown) are displayed in the bottom row.

While encouraging, the results obtained for the cyclization of synthetic model peptides do not accurately reflect the challenges associated with achieving chemo- and regioselective modification of linear peptides displayed on bacteriophages, which is a prerequisite for the application of this strategy in biological selections. Toward this end, a sequence encoding for the peptide **H**_**2**_**N**-AVSSGG**C** was appended to the N-terminus of the soluble D1D2-domains of a cysteine-free phage-coat protein III (pIII, Figure 4a).^55^ Additionally, we inserted the recognition site for the tobacco etch virus (TEV) protease between the appended peptide and the D1D2 domains in order to identify cyclized peptides following proteolysis (see the Supporting Information for details). The resulting C-terminally His_6_-tagged protein pep-TEV-D1D2 was subsequently produced in *Escherichia Coli* and purified by Ni^2+^-affinity chromatography (yield ∼ 100 mg/L), and its identity and purity were confirmed by sodium dodecyl sulfate–polyacrylamide gel electrophoresis and UPLC-MS (Figure 4b).

Addition of the bifunctional cyclization unit **1** (0.5 mM) to purified pep-TEV-D1D2 (0.1 mM) at pH 7 resulted in its conversion to a single, modified product over 5 h at 30 °C (Figures 4b and S10).^56^ Following removal of excess **1** by size exclusion and concomitant buffer exchange (to pH 6), this intermediate underwent quantitative conversion upon addition of NaBH_3_CN (five additions over 24 h, final concentration = 5 mM) to a species with a mass that is consistent with cyclization of the appended peptide sequence with **1**. Conversely, when performing the same procedure with the parent, cysteine-free D1D2 variant, we did not observe any appreciable levels of modification throughout the procedure (Figure S11). To verify that the cyclization of pep-TEV-D1D2 took place selectively via its N-terminal amine, we added TEV protease to the crude reaction mixture and analyzed the resulting cleavage products by UPLC-MS (Figures 4c and S10). Consistent with the high degree of selectivity observed in the model peptides, the only two species we detected following the addition of TEV protease were those with masses corresponding to (1) the cleaved protein and (2) the cyclic peptide. Comparable results were obtained when using bifunctional cyclization units **2–4**, which all yielded cleaved MPs following cysteine alkylation, reductive amination, and TEV protease treatment (Figures 4d and S10).

Notably, in these experiments, we were unable to detect either appreciable levels of double-modified pep-TEV-D1D2 or instances of the cyclization taking place via a lysine side chain. With all eight lysine residues in D1D2 being C-terminal of the TEV cleavage site, competing cyclization reactions would result in an intact pep-TEV-D1D2 species with a +18 Da-mass peak following TEV cleavage. We ascribe the lack of double modification and the high selectivity for the N-terminus to the removal of the excess linker by size exclusion following the initial cysteine alkylation step. As imine formation is reversible, aldehydes prone to condense with primary amines are therefore removed prior to the reduction step, resulting in singly cysteine-modified proteins. At this stage, the aldehyde of a cyclization unit can only condense with the nearby N-terminal amine (= proximity driven), facilitating the efficient conversion of appended, modified peptides to their cyclic counterparts.

### Phage Compatibility of the Head-to-Side-Chain Cyclization Strategy

**Figure 5:**
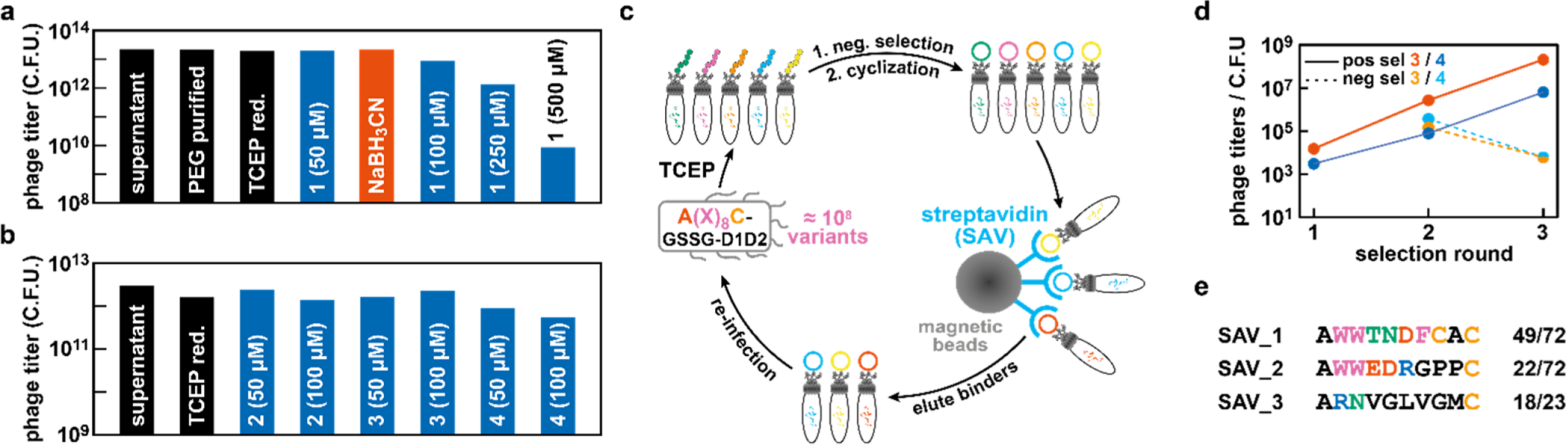
Phage compatibility and selection of streptavidin-binding MPs. (a,b) Phage titers measured as cfus when subjecting bacteriophages produced from *E. coli* TG1 cells to the two-step cyclization protocol. Bifunctional cyclization units (up to 100 μM) and NaBH_3_CN have a negligible effect on the life cycle of bacteriophages and give rise to comparable phage titers to those obtained after NaCl/PEG precipitation and TCEP reduction. (c) Schematic representation of the phage display cycle employed for the selection of MPs against streptavidin. For negative selections, phages featuring reduced peptides were incubated with streptavidin immobilized on magnetic beads. Cyclization reactions on phages that were not retained in the negative selection were performed with cyclization unit **3** or **4** in parallel (see the Supporting Information for details). (d) Phage titers corresponding to the number of retained phages after positive or negative selection per selection round. An increase in phage titers for the positive selections over consecutive rounds is indicative for the amplification of binders. (e) Sequences of peptides enriched after three rounds of selection in the presence of cyclization unit **3** (**SAV_1-2**) and **4** (**SAV_3**). *n*/*n* = number of times a sequence was identified by sequencing compared to the total number of sequencing runs for selections in the presence of **3** (*N* = 72) and **4** (*N* = 23).

To find applications in the selection of natural-product-like MPs by phage display, we investigated whether the conditions employed for our head-to-side-chain cyclization are compatible with the life cycle of bacteriophages. For this, we inserted the disulfide-free D1D2 domains of pIII featuring the model peptide sequence into the fd bacteriophage genome and produced virions following established protocols (see the Supporting Information for details).^36^ We reproducibly observed 10^12^ to 10^13^ infective phage particles in the supernatant when producing the resulting fd bacteriophages from commercially available *E. coli* TG1 cells (Figure 5a,b). With the appropriate bacteriophages in hand, we measured the infectivity of phage particles [=phage titers in colony forming units (cfus)] throughout all steps necessary for peptide cyclization (see the Supporting Information for details).

Gratifyingly, at 30 °C, neither the addition of **1** (50 μM) at pH 8 for 1 h nor the addition of NaBH_3_CN (five additions, final concentration = 2.5 mM) at pH 6 over 24 h at 4 °C had a significant impact on infectivity levels (Figure 5a). We chose pH 8 for the initial cysteine alkylation as these conditions have previously been used for comparable modifications on peptide-displayed libraries.^36^ Only upon increasing the concentration of **1** above 250 μM, we observed a ∼10-fold decrease in cfus following incubation at pH 8 at 30 °C for 1 h (Figure 5a). Last, the addition of bifunctional cyclization units **2–4**, which feature electrophilic bromoacetamide moieties, had also no apparent impact on phage infectivity at concentrations up to 100 μM (Figure 5b). Combined, these results are consistent with previous reports that independently found no significant impact on phage infectivity upon addition of either electrophilic crosslinkers or NaBH_3_CN to modify peptides on whole bacteriophages.^36,57,58^

### Selection of Streptavidin-Binding MPs

The apparent phage compatibility of our two-step cyclization strategy encouraged us to pursue phage selections of MPs featuring our asymmetric cyclization units against streptavidin as a model protein target. Thus, we constructed a naïve phage library (**H**_**2**_**N**-A(X)_8_**C**-GGSG-pIII) by appending a randomized octapeptide (X_8_) and a flexible linker (GGSG) to the pIII protein (see the Supporting Information for details). Following transformation of the vector into electrocompetent *E. coli* TG1 cells, we obtained ∼10^8^ colonies, as judged by the phage titers from plating serial dilutions of the transformation mixture.

To identify MP binders from this library, we performed phage selections with the asymmetric scaffolds **3** and **4** in parallel (Figure 5c). In order to deplete the pool of library members from linear streptavidin-binding peptides, a negative selection step was included prior to modification and cyclization (see the Supporting Information for details). Consistent with the enrichment of cyclic rather than linear streptavidin-binding peptides over consecutive rounds of biopanning and reinfection, we observed a significant increase in phage titers only for positive selections after cyclization but not for negative selections (Figure 5d). Notably, the choice of the bifunctional cyclization unit also impacted the outcome of the selection, with **3** consistently resulting in a higher number of library members being retained after each round of selection.

To identify potential streptavidin-binding MPs, we sequenced a total of 95 library members that were retained following three selection/reinfection cycles (72 and 23 from selections with **3** and **4**, respectively). Somewhat unexpected, these results revealed three peptides, **SAV_1–3**, which were strongly enriched in these samples and accounted for 89/95 submitted sequences (Figure 5e). We surmise that the low diversity of sequences is not only an indication of their enrichment but also a consequence of the relatively small size of the starting library (∼10^8^), which may have encoded only for a handful of streptavidin-binding MPs. Nevertheless, some notable observations can be inferred from the performed model selections: (1) none of the enriched sequences share similarity with streptavidin-binding linear (or cyclic) peptides that have been identified in previous (in vitro) selections^59^ and the absence of sequences such as the well-known HPQ motif attests on their successful depletion in the negative selection step. (2) Starting from identical libraries, vastly different peptide sequences were enriched when phage selections were performed with cyclization unit **3** or **4**. This result is consistent with different scaffolds giving rise to different ring sizes and/or peptide conformations/orientations that enable distinct binding modes. (3) Selection with **3** strongly enriched for sequences that contain an N-terminal Trp–Trp motive, while cyclization unit **4** appears to favor more hydrophobic sequences. (4) The presence of an additional cysteine residue in the most enriched peptide (**SAV_1**) might indicate a preference for smaller ring sizes in our cyclization strategy (vide infra).

### Validation and Characterization of Selected MPs

**Figure 6:**
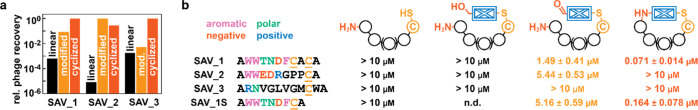
In vitro characterization of streptavidin-binding MPs. (a) Relative recovery rates of bacteriophages displaying linear, modified, or cyclized **SAV_1**/**SAV_3** peptides following biopanning against streptavidin. Values for **SAV_1** and **SAV_2** are normalized for phages subjected to cyclization conditions. (b) Apparent *K*_d,app_ values obtained from competitive ligand-binding analysis of **SAV_1-3** variants. For peptides that did not display saturation, a *K*_d,app_ of >10 μM is given; n.d. not determined.

To independently verify that our strategy selects peptide binders, we first produced phages displaying **SAV_1–3**. Next, we performed the modification and cyclization reactions as described previously and determined phage titers before and after biopanning against streptavidin for phages featuring the linear precursor, the modified and cyclized peptides (Figure S12, see the Supporting Information for details). Consistent with the selection of peptide binders upon addition of our bifunctional cyclization units, the recovery rates for linear precursors were the lowest for all three peptides (Figure 6A). Conversely, for both **SAV_1** and **SAV_3**, we observed the highest levels of retention for phages that have been subjected to our cyclization conditions. Unexpectedly though, for **SAV_2**, the modified rather than the cyclized peptide gave rise to the highest recovery rates. Featuring a reactive aldehyde moiety, this apparently higher affinity of the modified analogue of **SAV_2** could be the result of the transient formation of an iminium ion via condensation with either the N-terminal amine or a lysine side chain of streptavidin.

To pinpoint the binding mode of **SAV_1–3** variants, we first synthesized the linear precursors and then prepared their modified and cyclized versions (Figure S13). Notably, the cyclization of **SAV_1** that features a second cysteine closer to the N-terminus gave rise to a single cyclic species, which also formed faster than cyclic **SAV_2** and **SAV_3**. To further elucidate the role of the free aldehyde for binding, we additionally prepared **SAV_1–3** peptides that were modified with cyclization units bearing an alcohol moiety instead (Figures 6b and S13). Binding affinities for all peptide variants were then determined by employing a previously reported competition assay with 4′-hydroxy-azobenzene-2-carboxylic acid (HABA, see the Supporting Information for details).^60,61^

Consistent with the recovery rates observed with whole bacteriophages (Figure 6a), neither the linear precursors nor peptides featuring the alcohol displayed any affinity for streptavidin in the competition assay (Figures 6b and S14). Conversely, measuring aldehyde-modified and cyclic peptides of **SAV_1-3** revealed some notable differences. For once, we did not observe measurable affinities for any of the **SAV_3** variants, which likely indicates that they bind (unspecifically) to a different pocket than the one occupied by HABA. Conversely, for **SAV_2**, the aldehyde-bearing peptide proved to be a modest binder (*K*_d,app_ = 5.44 ± 0.53 μM) that could outcompete HABA (Figure S15). Given that the cyclic peptide did not display any affinity, binding of modified **SAV_2** is likely the result of the aldehyde moiety condensing with a lysine side chain of streptavidin (e.g., K121, which is located in the vestibule of the binding site).^62,63^

Gratifyingly, cyclic **SAV_1** proved to be a potent binder (*K*_d,app_ = 71 ± 14 nM) and outperformed the aldehyde-modified peptide by a factor of ∼20 (*K*_d,app_ = 1.49 ± 0.41 μM, Figures 6b and S15). As we anticipated that the cysteine closer to the N-terminal amine would be preferred for cyclization, we also prepared a shorter version of **SAV_1** (**SAV_1S**, Figures 6b and S16), which does not feature the original, C-terminal cysteine residue. Cyclic **SAV_1S** displayed similar affinity for streptatvidin as its longer counterpart (*K*_d,app_ = 164 ± 78 nM) while maintaining a ∼30-fold advantage over the modified peptide featuring the aldehyde (*K*_d,app_ = 5.16 ± 0.59 μM), which can form a transient imine with its N-terminal amine (Figures 6b and S17 and S18). We surmise that a restricted conformational flexibility and reduced entropic penalty upon binding are responsible for the observed differences between cyclic **SAV_1/SAV_1S** and their linear, modified counterparts. Overall, these studies attest on a straightforward integration of our two-step cyclization strategy into standard phage-display protocols to facilitate the selection of natural-product-like MP binders against protein targets.

## Conclusions

In summary, we have developed a selective two-step cyclization method that makes use of the programmed modification of a unique cysteine thiol and a nearby N-terminal amine. This strategy provides straightforward access to MPs starting from synthetic peptides or adequate sequences appended to proteins. The minimal requirements for bifunctional cyclization units—the presence of an (aromatic) aldehyde and an appropriate electrophile—should readily be installable onto a wide variety of asymmetric molecular scaffolds. Furthermore, the transiently formed iminium ion intermediate should lend itself to further diversification in the presence of nucleophiles other than NaBH_3_CN.^17,64^ As a result, we anticipate that our strategy will prove to be valuable for accessing cyclic analogues of existing bioactive MPs,^15,16,65−67^ where diverse cyclization units can be used for fine-tuning the properties of these MPs.

Furthermore, the compatibility of our cyclization strategy with whole bacteriophages enabled us to perform phage selections against streptavidin. Incorporating our head-to-side-chain cyclization strategy into a standard phage-display workflow facilitated the selection of an MP that displayed <100 nM affinity for this model protein. We also demonstrated that the choice of the bifunctional cyclization unit influenced the outcome of the selection, with distinct peptide sequences being enriched in response to different bifunctional scaffolds. These results augur well for a combinatorial approach, in which starting from a single library, the addition of different cyclization units gives rise to diverse binders.

The successful selection of an MP binder encourages the application of our approach to biologically relevant targets in the near future. Particularly, the ability to incorporate asymmetric scaffolds promises a chemogenetic optimization akin to the evolutionary processes that gave rise to natural MPs. Allowing for the incorporation of non-peptidic moieties that (1) can serve as cyclization units, (2) provide interactions for binding, and/or (3) tailor pharmacological properties, we are confident that our head-to-side-chain cyclization strategy will provide access to a currently under-explored chemical space. Being able to search this chemical space efficiently by phage display promises many opportunities for ligand diversification in drug discovery efforts and the development of chemical probes.

## Supporting Information Available

Experimental procedures, synthesis of peptides and bifunctional cyclization units, molecular biology operations, phage display protocols, and binding studies (PDF)
