# Supplementary material for: A Strategy to Select Macrocyclic Peptides Featuring Asymmetric Molecular Scaffolds as Cyclization Units by Phage Display
Source: J Am Chem Soc. 2022 Feb 16;144(8):3644–52. doi: 10.1021/jacs.1c12822 (PMC8895403; doi:10.1021/jacs.1c12822)
Supplement: Supplementary file 1 — ja1c12822_si_001.pdf [file ja1c12822_si_001.pdf]

Supporting Information

A strategy to select macrocyclic peptides featuring asymmetric molecular scaffolds as cyclization units by phage display

*Titia Rixt Oppewal, Ivar D. Jansen, Johan Hekelaar and Clemens Mayer**

Table of contents

1. Supporting discussion of 1D and 2D NMR experiments S2
2. Supporting Figures S4
3. Material & Methods S14
4. Solid phase peptide synthesis S17
5. Chemical Synthesis S20
6. Peptide cyclization reactions S25
7. Molecular Biology S27
8. Protein cyclization and TEV cleavage S30
9. Phage production and infectivity studies S31
10. Library preparation S33
11. Phage selections S36
12. HABA competition binding assay S39
13. Isothermal titration calorimetry S42
14. Primer table S43
15. NMR spectra S44
16. References S55

1. Supporting discussion of 1D and 2D NMR experiments

Figure S1: A comparison of ^{1}H-NMR spectra obtained for synthetic pep1 before and after modification with **1** shows the appearance of signals consistent with the para-substituted benzene and aldehyde protons of the cyclization unit. Moreover, the two β-protons of cysteine are split in the modified sample, which is consistent with a conformational restriction upon thiol alkylation. Notably, for the ^{1}H-NMR spectrum obtained following reduction with $NaBH_3CN$ individual β-protons of cysteine become even more distinct, which is consistent with cyclization. Similarly, we noted the disappearance of the aldehyde proton and the concomitant shift of one pair of aromatic protons, which are consistent with the change from the benzaldehyde to the corresponding benzylamine moiety. Similarly, we observe two new signals around 4 and 4.3 ppm, which were assigned to the benzylic protons formed upon reductive amination.

Figure S2: For the comparison of ^{1}H total correlated spectra (TOCSY) spectra between the synthetic (**A**), modified (**B**) and cyclized peptide (**C**), we focused on identifying significant shifts of amide protons. As amide protons form a spin-system with protons from their respective side chains, this analysis enabled us to assign individual amide protons to their corresponding amino acids in all three spectra. For example, cysteine alkylation has little effect on the environment of most amide protons, with exception of the small shifts observed for those corresponding to the C-terminal alanine, tryptophan and glycine residues (spectrum **A** and **B**). As such, this result is indicative for an interaction of the newly installed benzene ring from the cyclization unit with these residues. In stark contrast, amide protons in the TOCSY spectrum of the reduced product have undergone drastic changes. Particularly, signals corresponding to serine and glycine residues shift by approximately 0.5 ppm. This difference is consistent with the formation of a cyclic product that will require these flexible amino acids to adopt different

conformations. Moreover, the splitting of α- and β-methylene protons of glycine and serine residues, respectively can be observed, which is also consistent with the formation of a MP.

Figure S3: As stated in the main text of the manuscript, we performed Nuclear Overhauser Effect Spectroscopy (NOESY) on the crude cyclized product after modification of pep1 with **1** and subsequent reduction with $NaBH_3CN$. Note that the spectrum was recorded at a lower temperature (4 °C vs 25 °C) than the ^{1}H-NMR and TOCSY spectra discussed before to improve the signal-to-noise ratio of NOEs over background. In addition to the signals observed for the aromatic protons of **1** (**Fig. 2C**), we also identified NOEs between the benzylic protons and those of cysteine and the N-terminal alanine residue. Particularly, this analysis revealed that the alpha and beta-protons of cysteine exclusively interact with the benzylic protons with a shift of 4.45 ppm, while the corresponding alanine protons give rise to NOEs with the second set of benzylic protons at 3.8 and 4.1 ppm.

2. Supporting Figures

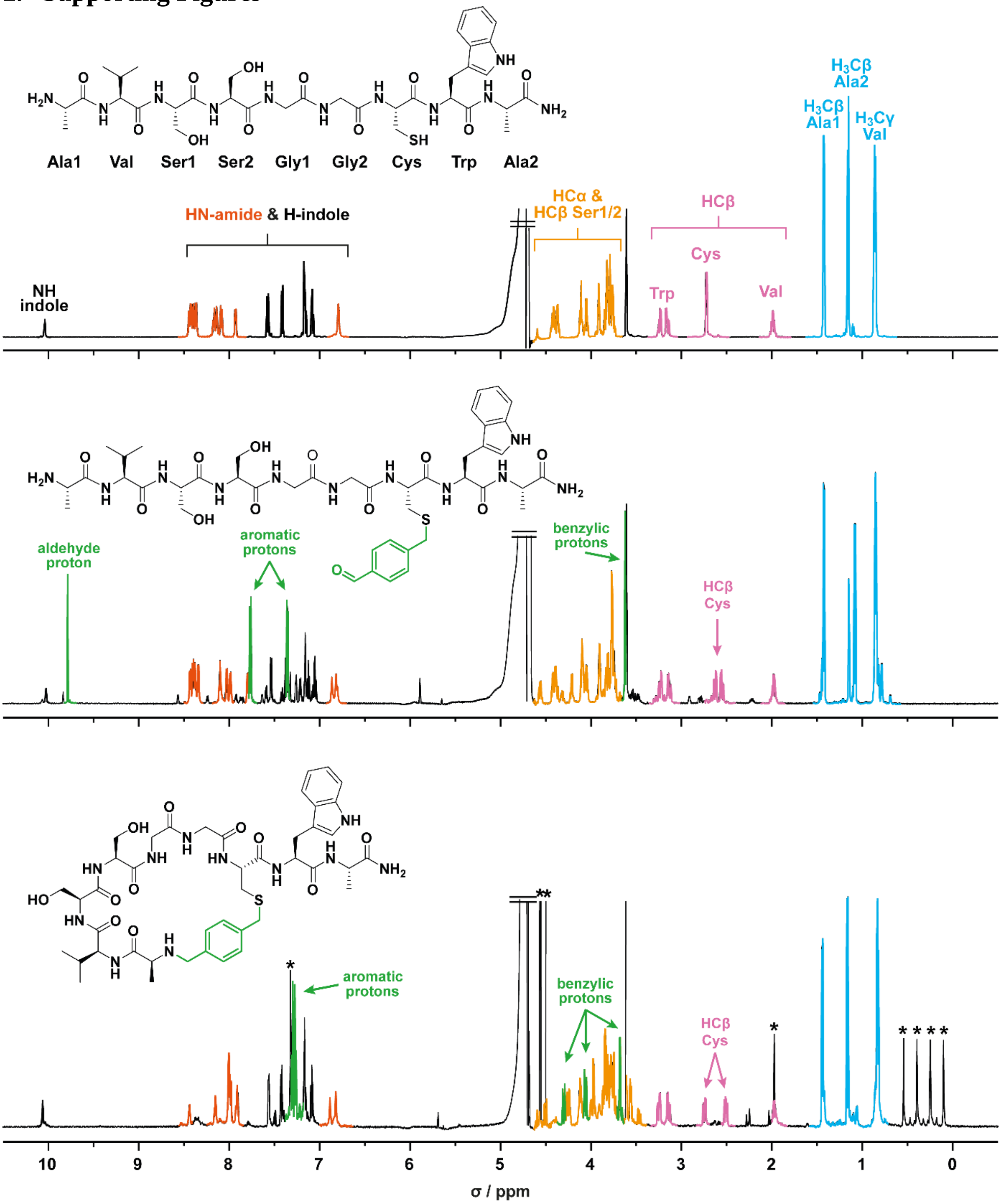

Figure S1: ^{1}H-NMR of synthetic pep1, after cysteine alkylation with **1** and following cyclization in presence of $NaBH_3CN$. See Supporting Discussion for an analysis of the most relevant changes. Relevant peaks are highlighted by different colors. Peaks assigned with a * are ascribed to hydrolysis/decomposition products of $NaBH_3CN$.

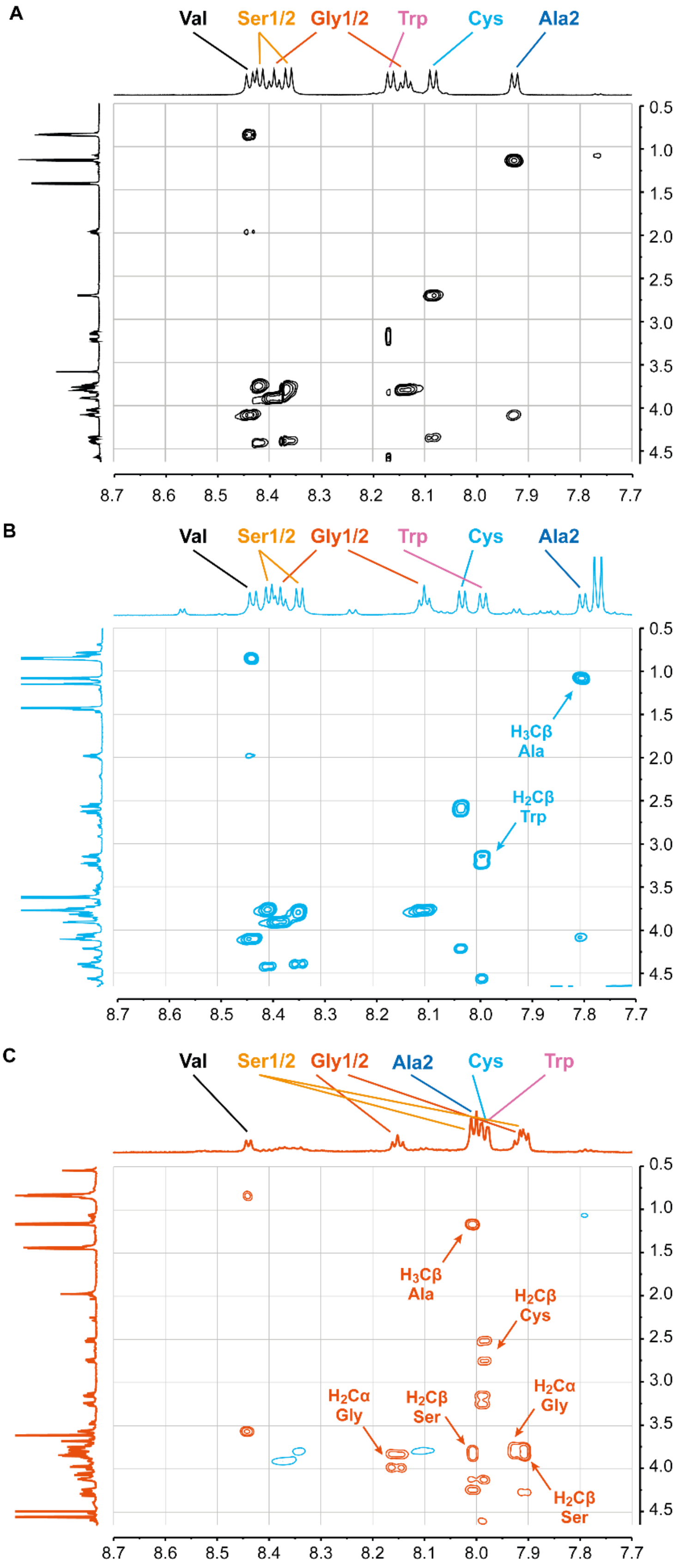

Figure S2: ^{1}H-TOCSY spectra highlighting the spin systems between amide protons and amino acid side chains of synthetic pep1 (**A**), after cysteine alkylation with **1** (**B**) and following cyclization in presence of $NaBH_3CN$ (**C**). Residual signals resulting from incomplete conversion to the MP are highlighted in light blue in panel **C**. See Supporting Discussion for the interpretation of the most relevant changes in-between spectra.

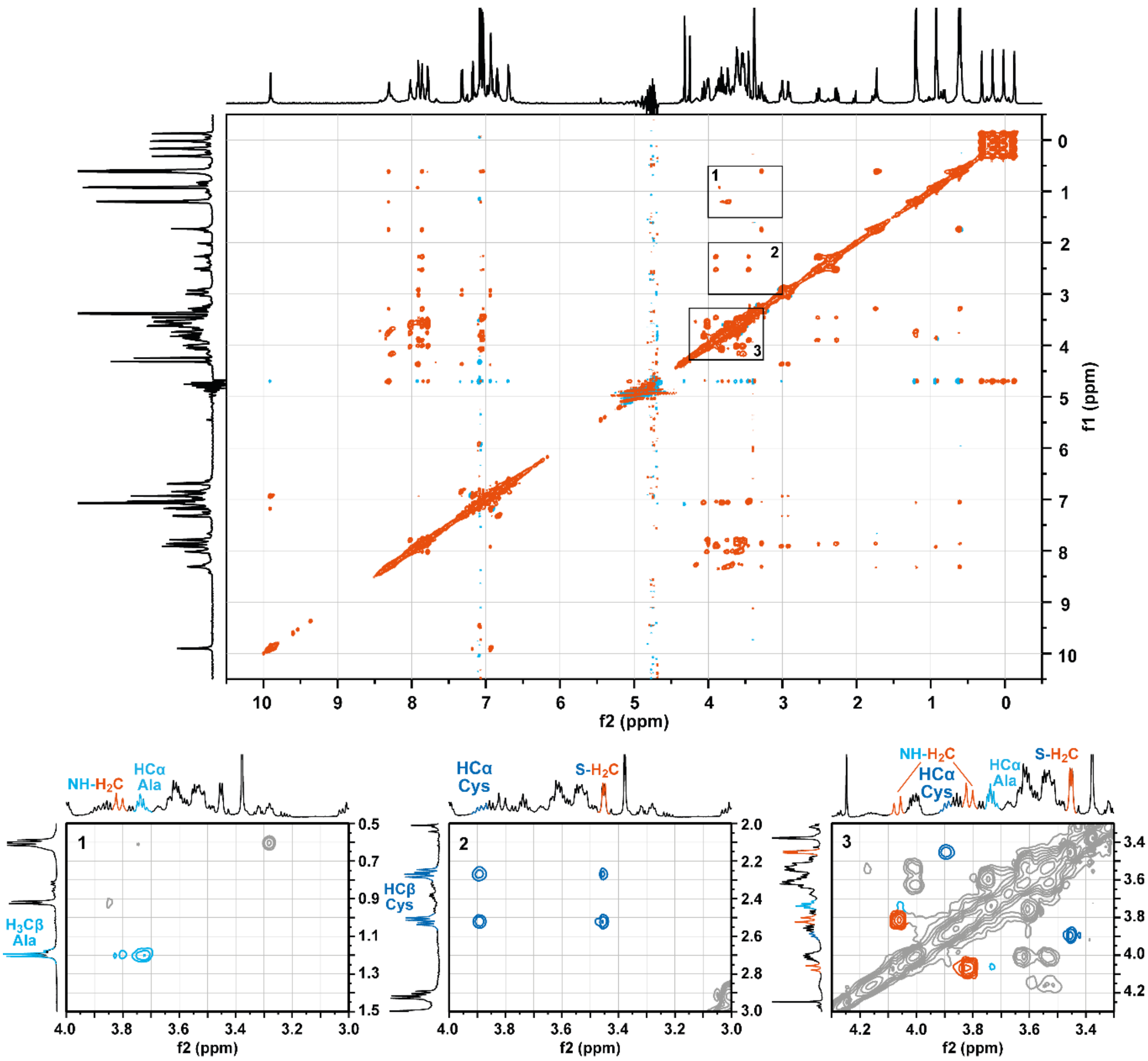

Figure S3: ^{1}H-NOESY obtained from the crude reaction mixture following cyclization of pep1 with **1**. Inserts show critical NOEs observed between the benzylic protons of the bifunctional cyclization unit and the N-terminal alanine residue as well as the unique cysteine. See Supporting Discussion for the interpretation of the most relevant changes in-between spectra.

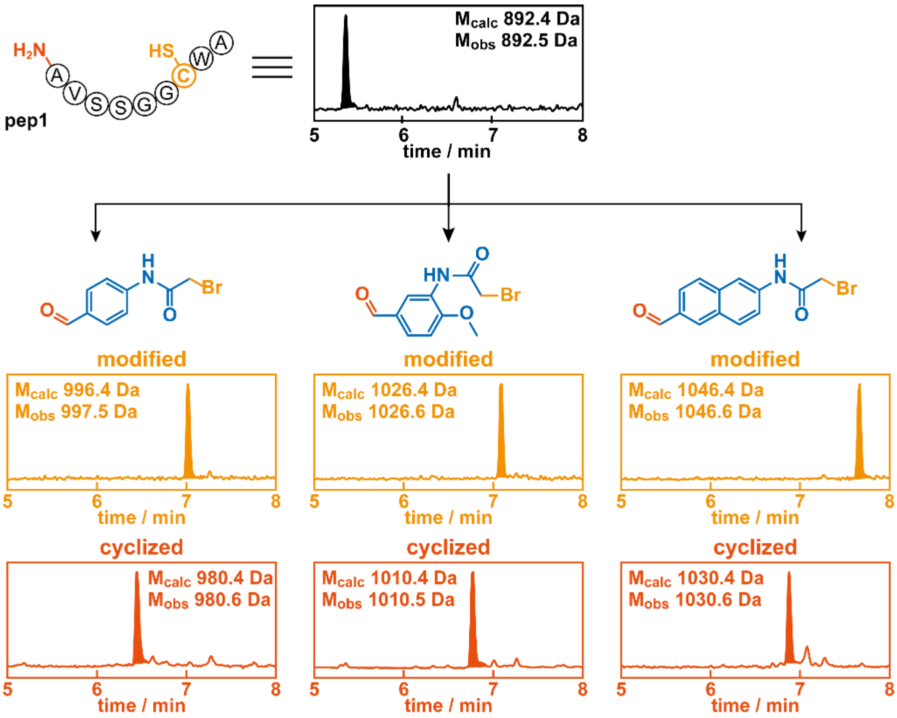

Figure S4: Representative UPLC-MS chromatograms of synthetic pep1 (top) and crude reaction mixtures obtained following reaction with bifunctional cyclization units **2-4** (middle) and cyclization in presence of $NaBH_3CN$ (bottom). Total ion counts (TICs) are depicted with masses found for the major species inserted.

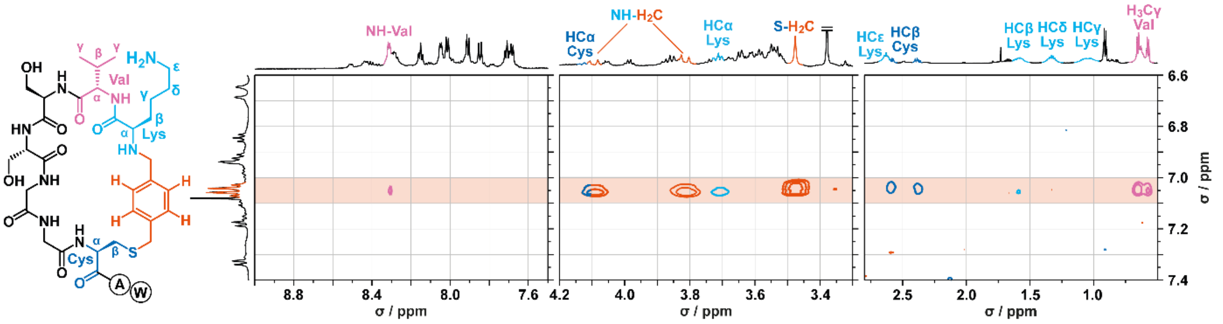

Figure S5: The structure of the obtained cyclic product from the reaction of pep4 with **1** as well as excerpts from a 2D NOESY spectrum are shown. NOEs between the phenylic protons and amino acid residues are highlighted. Color code: phenylic protons = red, cysteine protons = dark blue, lysine protons = light blue, valine protons = magenta. Critically, NOEs are only observed for the α- and β-protons of lysine but are absent for γ-ε protons. This observation is consistent with the cyclization occurring selectively via the N-terminus instead of the ε-amine. Note that excerpts of the spectrum have been scaled independently to highlight the weak NOEs observed between phenylic protons and the NH-Val as well as the H2Cβ-Lys**.**

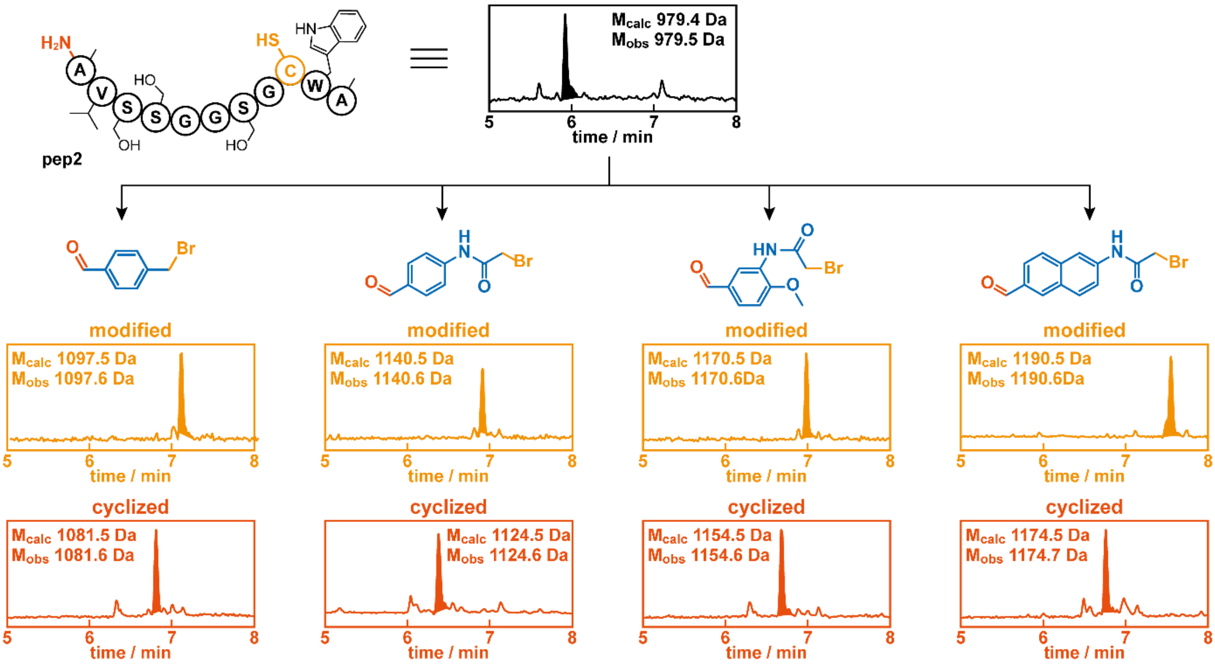

Figure S6: Representative UPLC-MS chromatograms of synthetic pep2 (top) and crude reaction mixtures obtained following reaction with bifunctional cyclization units **1-4** (middle) and cyclization in presence of $NaBH_3CN$ (bottom). Total ion counts (TICs) are depicted with masses found for the major species inserted.

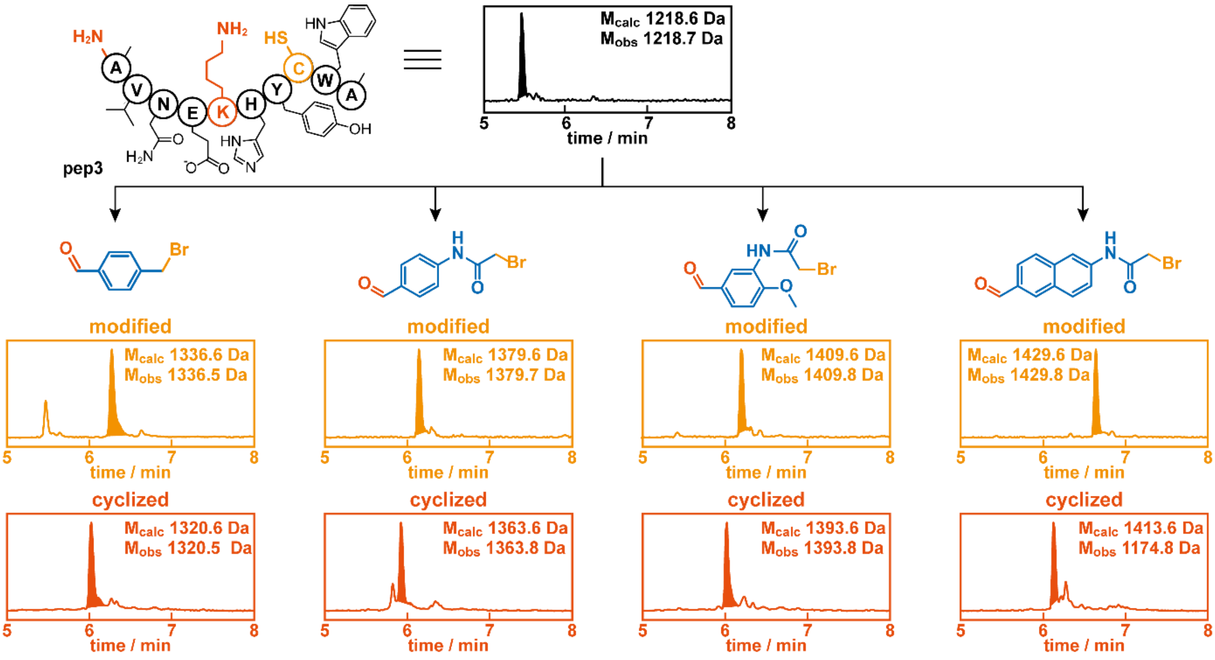

Figure S7: Representative UPLC-MS chromatograms of synthetic pep3 (top) and crude reaction mixtures obtained following reaction with bifunctional cyclization units **1-4** (middle) and cyclization in presence of $NaBH_3CN$ (bottom). Total ion counts (TICs) are depicted with masses found for the major species inserted.

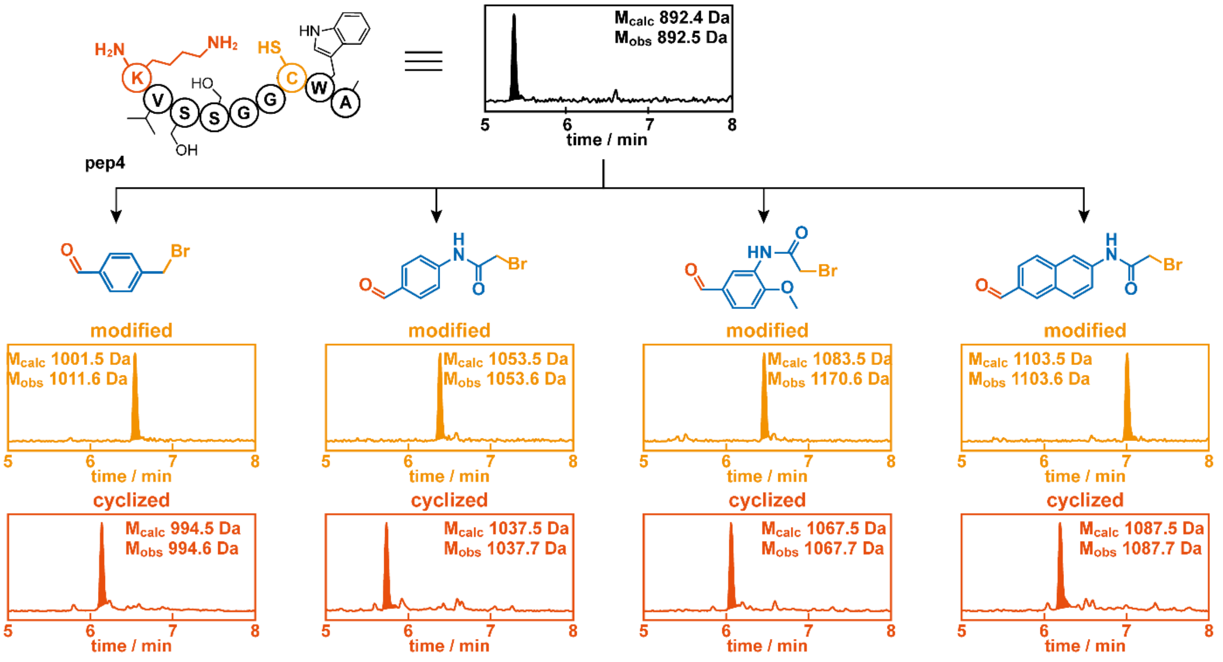

Figure S8: Representative UPLC-MS chromatograms of synthetic pep4 (top) and crude reaction mixtures obtained following reaction with bifunctional cyclization units **1-4** (middle) and cyclization in presence of $NaBH_3CN$ (bottom). Total ion counts (TICs) are depicted with masses found for the major species inserted.

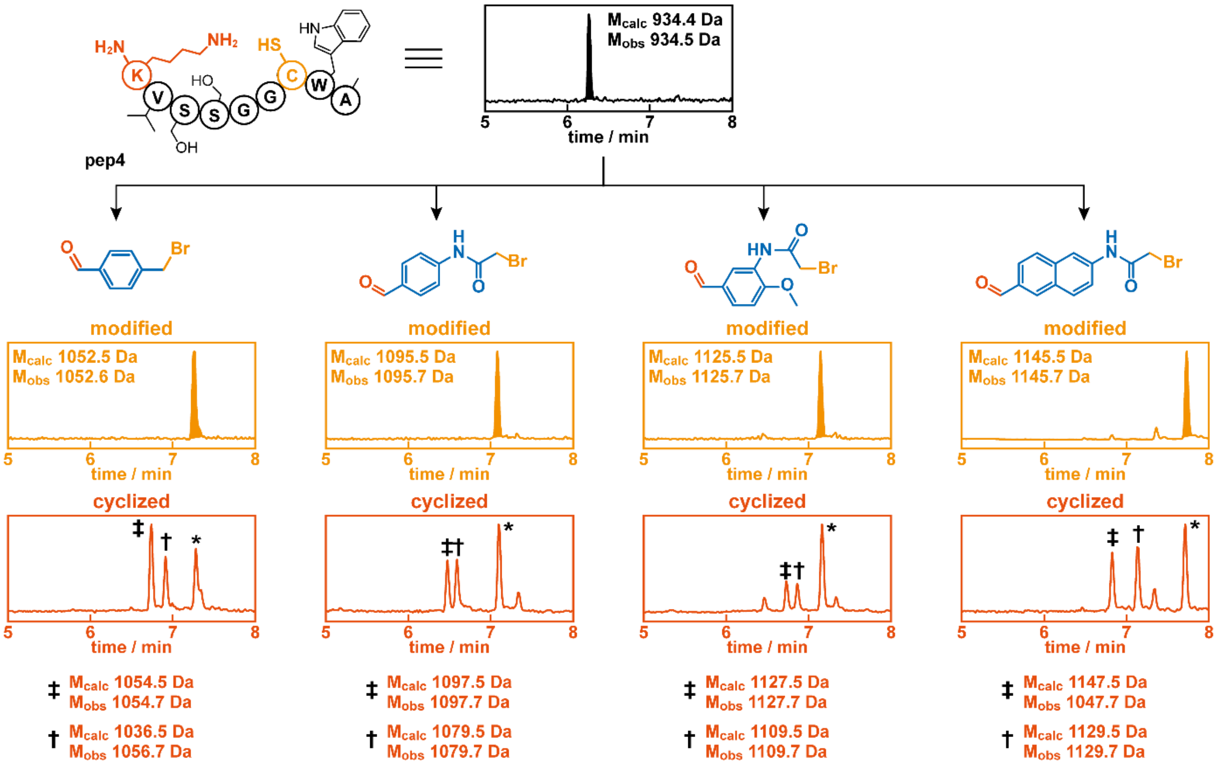

Figure S9: Representative UPLC-MS chromatograms of synthetic pep5 (top) and crude reaction mixtures obtained following reaction with bifunctional cyclization units **1-4** (middle) and cyclization in presence of $NaBH_3CN$ (bottom). Total ion counts (TICs) are depicted with masses found for the major species displayed.

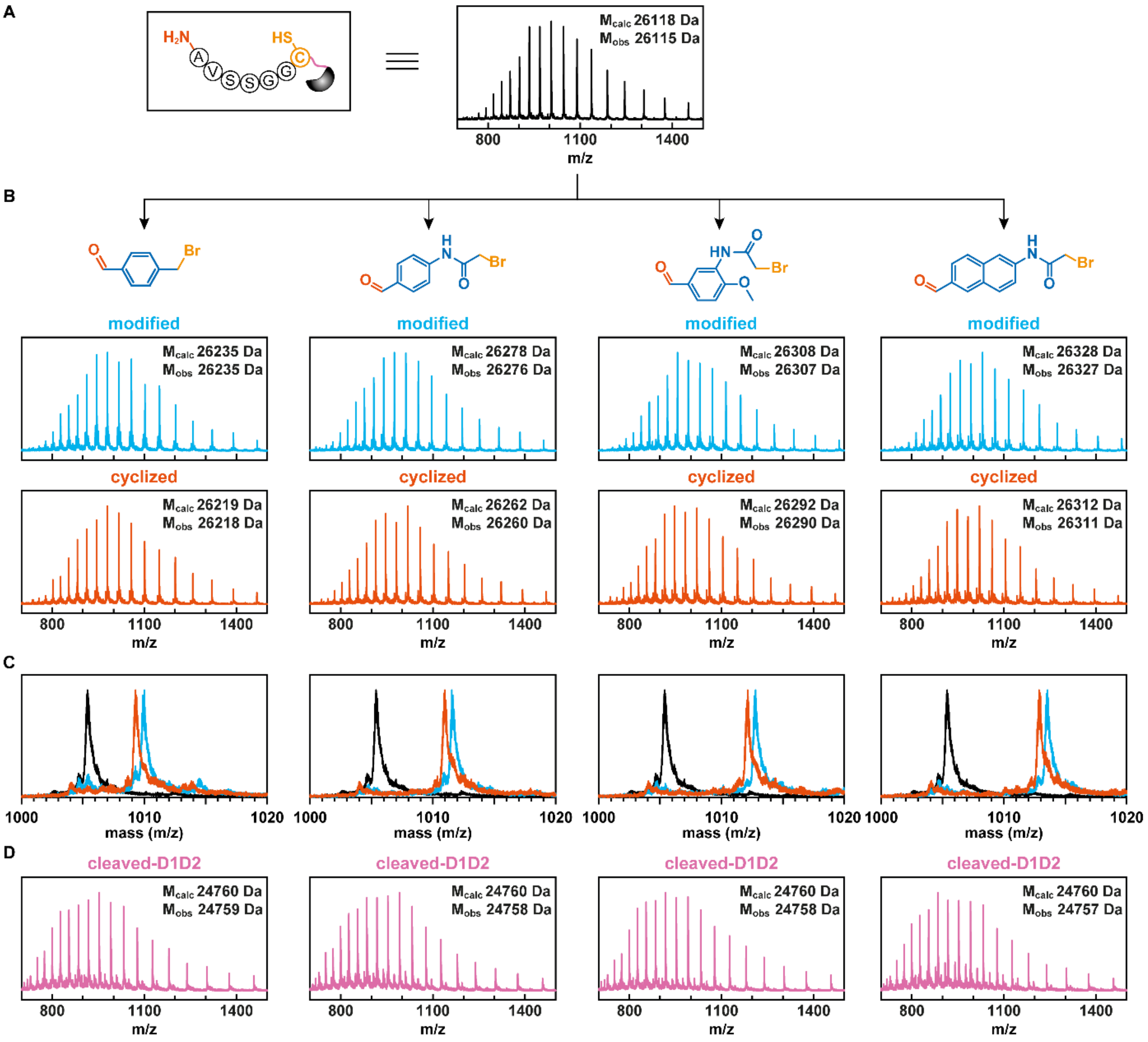

Figure S10: A-B: Representative raw mass spectrum and deconvoluted mass obtained for purified pep-TEV-D1D2 (**A**) and crude reaction mixtures obtained following modification and cyclization with **1-4** (**B**). **C:** Zoom-in of the +26 m/z peak around 1000-1020 Da for pep-TEV-D1D2 (black), following modification (blue) and cyclization (red). The formation of a distinct species following reductive amination demonstrates the selective formation of cyclic peptides on pep-TEV-D1D2. **D:** Representative raw mass spectrum and deconvoluted mass obtained for the protein fragment following TEV protease treatment.

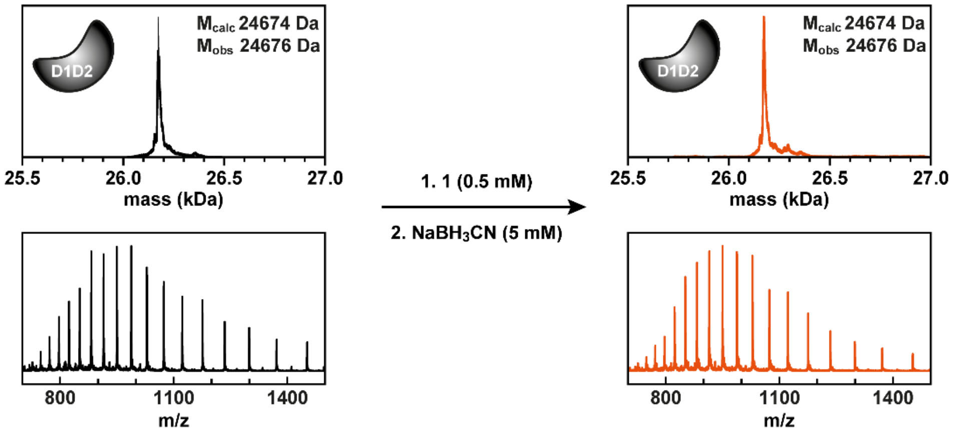

Figure S11: Representative raw mass spectra obtained for purified disulfide-free D1D2 and the crude reaction mixture following modification of D1D2 with **1** and $NaBH_3CN$ treatment. As stated in the main text, we were unable to observe any appreciable levels of modification in absence of the appended peptide.

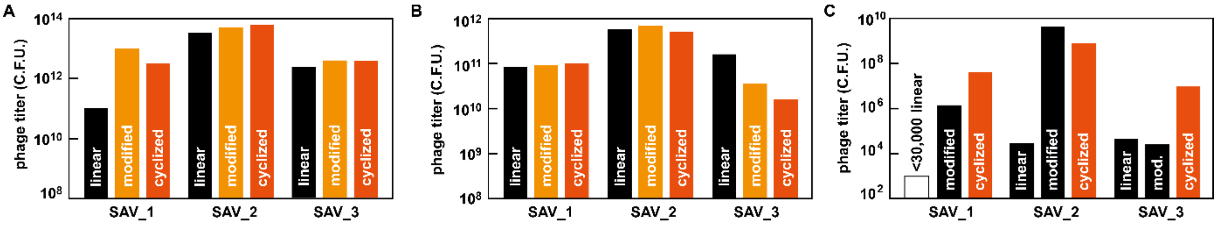

Figure S12: A-B: Phage titers obtained for linear, modified and cyclized phages displaying **SAV_1**, **SAV_2** or **SAV_3** before (**A**) and after dilution to approximately 10^{11} c.f.u.s (**B**). **C:** Phage titers obtained after biopanning bacteriophages featuring linear, modified and cyclized **SAV_1**, **SAV_2** or **SAV_3** against streptavidin. Note that no colonies for the linear **SAV_1** sample were obtained at the dilutions that were plated, resulting in an upper limit of 30,000 c.f.u.s for this sample.

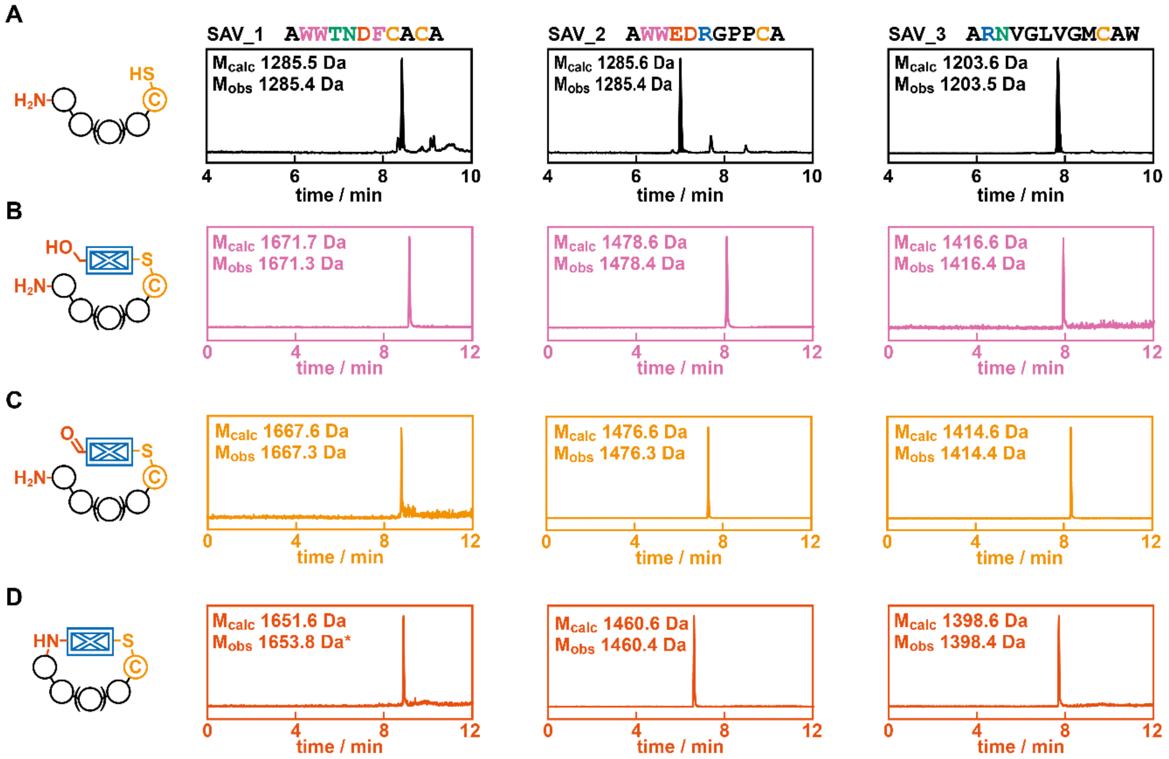

Figure S13: A-C: A: Representative UPLC-MS chromatograms of crude SAV_1-3 **B-D:** HPLC-purified modified and cyclized peptides. **B:** SAV_1-3 modified with alcohol containing scaffolds **S5** (SAV1 and SAV2) and **S8** (SAV3). **C:** SAV_1-3 modified with aldehyde containing scaffolds **3** (SAV1 and SAV2)

and **4** (SAV3). **D:** SAV_1-3 cyclized with scaffolds **3** (SAV1 and SAV2) and **4** (SAV3). Total ion counts (TICs) are depicted with masses found for the species inserted. *In panel **D** the observed mass for SAV1_cyclized is 2 Da higher than calculated due to reduction of the aldehyde on the scaffold that modified the second cysteine residue.

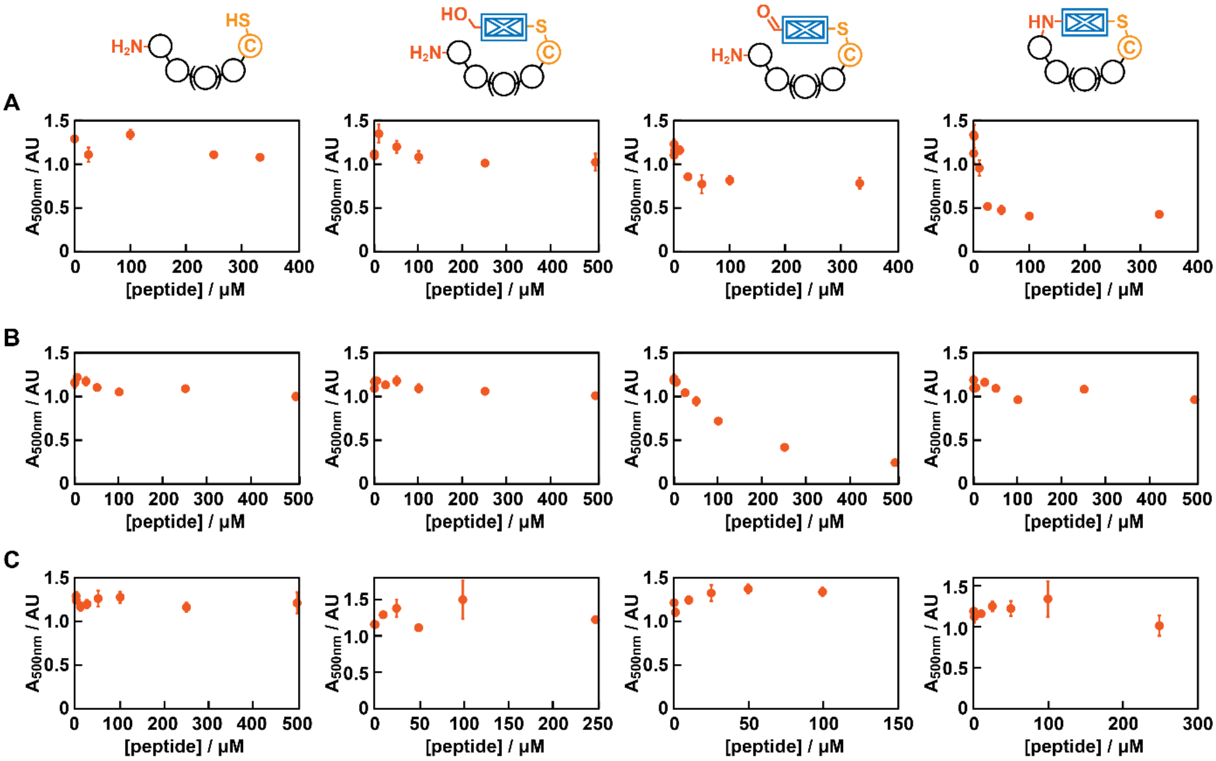

Figure S14: A-C: Results of competition assay with HABA for SAV_1-3 (**A-C**). A500nm values were measured after addition of linear, modified and cyclic SAV_1-3 variants to a preformed streptavidin-HABA complex. A500nm values displayed are the result of measurements performed in triplicate with standard deviations shown.

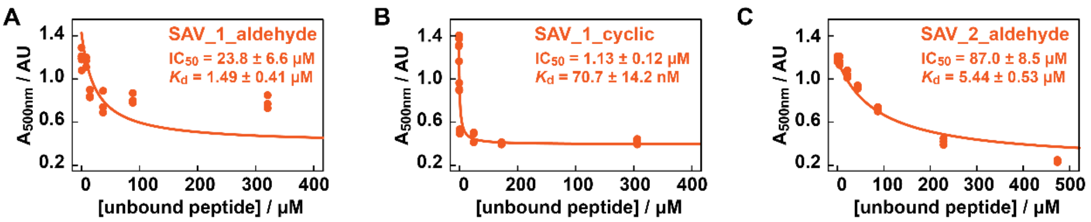

Figure S15: A-C: Competitive ligand-binding analysis of modified (**A**) and cyclic (**B**) SAV_1, as well as modified SAV_2 (**C**). Data points correspond to the A500nm measured after addition of peptides to a preformed streptavidin-HABA. IC_{50} and K_d values were obtained after fitting the data points to the Hill equation (see Supporting Information for details). Note, that no fit could be obtained for any of the other measurements displayed in Figure S14.

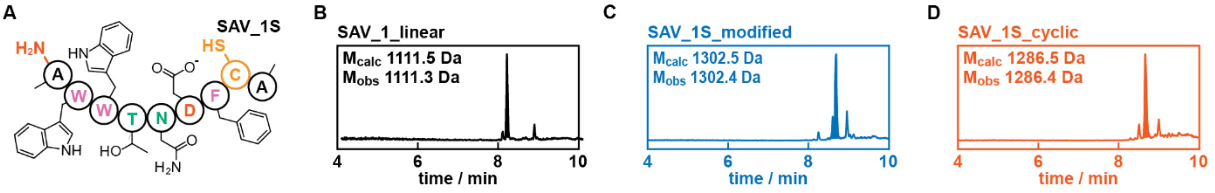

Figure S16: A: Schematic representation of **SAV_1S**. **B-D:** Representative UPLC-MS chromatograms of synthetic **SAV_1S** (**B**) and crude reaction mixtures obtained following reaction with bifunctional cyclization units **3** (**C**) and cyclization in presence of $NaBH_3CN$ (**D**). Total ion counts (TICs) are depicted with masses found for the major species inserted.

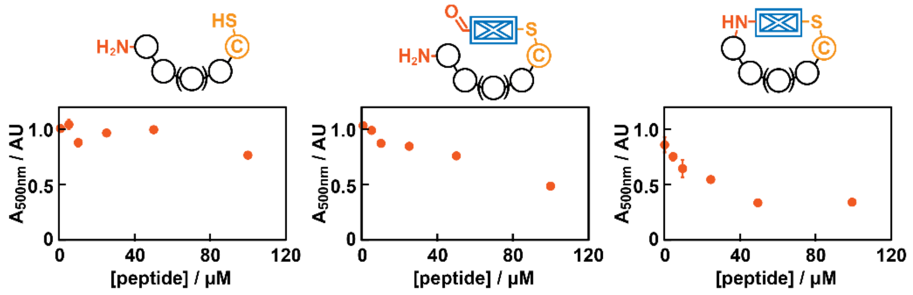

Figure S17: Results of competition assay with HABA for SAV_1S (**A-C**). A500nm values were measured after addition of linear, modified and cyclic SAV_1S variants to a preformed streptavidin-HABA complex. A500nm values displayed are the result of measurements performed in triplicate with standard deviations shown.

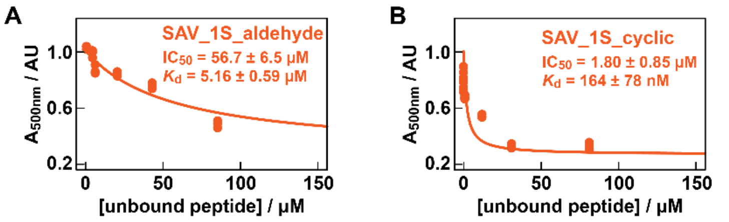

Figure S18: A-B: Competitive ligand-binding analysis of modified (**A**) and cyclic (**B**) SAV_1S. Data points correspond to the A500nm measured after addition of peptides to a preformed streptavidin-HABA. IC_{50} and K_d values were obtained after fitting the data points to the Hill equation (see Supporting Information for details). Note, that no fit could be obtained for linear SAV_1S.

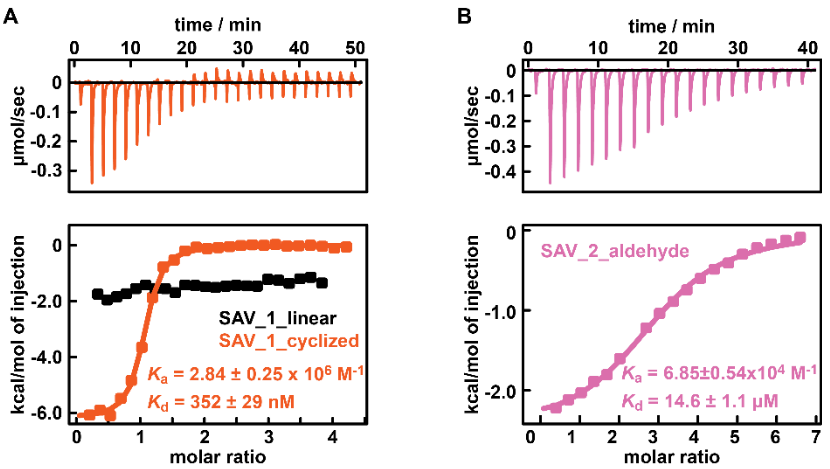

Figure S19: A-B: Isothermal titration calorimetry (ITC) measurements of linear and cyclic SAV_1 (**A**) and aldehyde-modified SAV_2 (**B**). To independently verify the extrapolated K_d values obtained from the HABA competition assay, we performed ITC measurements. The obtained binding affinities are 3-5 times higher than those determined by the competition assay, but do show the same relative difference in binding strength. Note, that data points correspond to values obtained from a single experiment.

3. Materials & Methods

Chemicals were used without further purification unless otherwise noted. All chemicals used in organic synthesis were purchased from *SigmaAldrich* or *TCI Europe*. ChemMatrix® Rink amide resin was purchased from *SigmaAldrich*. Solid-phase reaction vessels (syringes with filter) were purchased from *Torviq*. Fmoc-protected amino acids, HCTU, trifluoro acetic acid (TFA) and OxymaPure® were purchased from *ChemImpex Inc*. Solvents for solid phase peptide synthesis (dichloromethane (DCM) and dimethylformamide (DMF)) were purchased from *Biosolve*. DIPEA and piperidine was purchased from *Iris Biotech GMB*. Analytical thin-layer chromatography was carried out on pre-coated silica gel on aluminum sheets (*Merck* TLC Silica gel 60 / Kieselguhr F254), columns were performed using silica-P flash silica gel from *Silicycle* (0.040-0.063 mm 230 400 mesh). ^{1}H-NMR and ^{13}C-NMR spectra were recorded on a Bruker 400 MHz in Methanol-*d4*, $CDCl_3$ or DMSO-*d6*. 1D and 2D NMR spectra of peptides were performed in 90% H_2O/ 10% D_2O/ trifluoroacetic acid and recorded on a Bruker 600 MHz spectrometer. The strongest solvent peak was suppressed using excitation sculpting. Spectra were recorded either at 25 °C or at 4 °C. HSQC, TOCSY, and NOESY (120 ms mixing time) spectra were performed.

Analytical UPLC-MS analysis was performed on an Acquity UPLC system (Waters) coupled to a quadrupole/time-of-flight (QToF) mass spectrometer (Waters) equipped with a PDA detector. The peptides were separated on an Acquity BEH C8; 150 × 2.1 mm, 1.7 µm (Waters) column operated at 40°C. The eluent system employed was a combination of A (0.1% formic acid in water) and B (0.1% formic acid in acetonitrile) at a flow rate of 0.3 mL/min. The gradient varied linearly from 5 to 60% B (v/v) from 0-10 min., 60 to 95% B from 10-11 min., kept at 95% B from 11-13 min., returning to 5% B in 2 min., re-equilibration to 5% B from 15-20 min. Protein samples were separated on an Acquity BEH C4; 150 × 2.1 mm, 1.7 µm (Waters) column

operated at 40°C. The gradient varied linearly from 15 to 70% B (v/v) from 0-10 min., 70 to 95% B from 10-11 min., kept at 95% B from 11-13 min., returning to 20% B in 2 min., re-equilibration to 15% B from 15-20 min. The sample injection volume was 3 μL. Mass spectra were obtained in the ESI-positive ion mode over a mass range between 500 to 1500 Da at resolution >20.000 FWHM. Peptide samples were diluted to 100 μM and protein samples were diluted to 15 μM prior to analysis. Obtained charge density spectra were deconvoluted using the MagTran software.[1] Peptides SAV_1S_modified and SAV_1S cyclic were purified using a BUCHI Reveleris® Prep MPLC chromatography system with a PrepChrom HPLC C18; 150 × 21.2 mm, 10 μm (BUCHI) column operated at room temperature.

Reversed-phase HPLC was performed on a Shimadzu HPLC system equipped with LC-20AD solvent chromatographs, a DGU-20A3 degasser unit, a SIL-20A autosampler, a SPD-M20A PDA detector, a CTO-20A column oven operating at 40 °C, a CBM-20A system controller and a FRC-10A fraction collector. Preparative HPLC was performed on a Waters XSelect CSH C18 OBD™ prep column (10 x 250 mm, particle size 5 μm) using a flow of 4 mL/min. Eluents used were 0.1 % TFA in ACN (solvent A) and 0.1 % TFA in ddH2O (solvent B), using a gradient of 5% A (0-10 min.) to 70% A (10-50 min.) to 90% A (50-55 min.) remaining at 90% A (55-60 min.), returning to 5% A (60-70 min.) and re-equilibrating for 20 min. at 5% A (total runtime 90 minutes). Peptides were dissolved in 70/30 DMSO/ 0.1% TFA MQ and up to 1200 μL was injected per purification run.

Isothermal titration calorimetry (ITC) measurements were performed on a MicroCal® iTC200 MicroCalorimeter. Measurements were performed at 25 °C, with a reference power of 6 μcal/sec and with a stirring speed of 750 rpm.

Plasmid pET21b(+), bearing the ampicillin resistance gene was purchased from *Novagen*®. *Escherichia coli* strain NEB10-beta (*New England Biolabs*) was used for cloning and Primers

were synthesized by *Eurofins MWG Operon* (Germany). Plasmid Purification Kits were obtained from *QIAGEN* (Germany) and DNA sequencing carried out by *Eurofins* (Germany). Phusion polymerase, T4 ligase, and *NdeI*, *XhoI*, *SfiI*, and *EcoRI* were purchased from *New England Biolabs*. Ni-NTA resin (Ni SepharoseTM 6 Fast Flow) from *GE Healthcare Life Sciences* (Germany). Concentrations of DNA and protein solutions were determined based on the absorption at 260 nm or 280 nm on a Thermo Scientific Nanodrop 2000 UV-Vis spectrophotometer. Theoretical molecular weights of proteins were calculated using the Expasy ProtParam tool (http://web.expasy.org./protparam/). Cellular density (OD_{600}) was measured on an Ultrospec 10 Cell Density Meter (Biochrom).

E. coli TG1 cells (*Agilent*) were used for working with bacteriophages. Phage vectors fdg3p0ss21 and fd0D1D2' were kindly provided by Prof. Christian Heinis (LPPT group, EPFL Lausanne), with permission from Prof. F.X. Schmid. Buffers and solutions: PEG/NaCl: 20% PEG-6000 (w/v), 2.5 M NaCl (Sigma Aldrich) stored at 4 °C; reaction buffer: 20 mM HEPES, 5 mM EDTA, pH 8.0; reduction buffer: 50 mM MES, pH 6 (pH adjusted with NaOH and HCl; the buffers were degassed by applying a vacuum and stirring with a magnet for 3 h prior to use). Reagents: tris(2-carboxyethyl)phosphine (TCEP), 20 mM stock in H_2O (stored at 4 °C for up to 1 month); bifunctional scaffold, 50 mM stock in acetonitrile (prepared fresh). Amicon® Ultra-15 (100 kDa & 30 kDa) Centrifugal Filter Units (*SigmaAldrich*).

4. Solid phase peptide synthesis

Loading of Rink amide resin (coupling of Fmoc-Ala-OH: Rink amide resin loading = 0.4-0.6 mmol/g, 0.6 mmol/g was used as 1 eq.) was swollen in dry DMF for 1 and the resin subsequently washed with DCM (3 x 5 mL) and DMF (3 x 5 mL). Fmoc-Ala-OH (5 eq.) was pre-activated for 2 minutes in DMF (final concentration 0.5 M) following the addition of HCTU (4.7 eq.), OxymaPure® (4.7 eq.) and DIPEA (12 eq.). The preactivated Fmoc-protected amino acid was transferred to the resin and the resulting mixture was agitated (bubbling N_2 through the syringe) for 2 hours at room temperature. The resin was subsequently drained and another freshly-prepared batch of pre-activated Fmoc-Ala-OH was added. The resulting mixture was agitated for 3 hours, before the resin was drained and washed with DMF (3 x 5 mL), DCM (3 x 5 mL), and DMF (5 x 5 mL). All remaining, unreacted amine groups were capped by adding a solution of acetic anhydride:pyridine (3:2) to the resin. After agitating the mixture for 30 minutes, the resin was washed with DMF (4 x 5 mL), DCM (2 x 5 mL), DMF (3 x 5 mL), and DCM (5 x 5 mL). The resin was then dried under vacuum and stored at -20 °C until further use. A small sample (10 mg) of the dried resin was removed to determine the loading efficiency. For this, the resin was first swollen for 30 minutes in 800 μL of DMF, after which 200 μL of piperidine was added. The mixture was vortexed to ensure good mixing and left in a tabletop shaker (room temperature, 300 rpm) for 15 min. An aliquot of the mixture (100 μL) was diluted to 10 mL with 20% piperidine in DMF and the concentration of the piperidine-fulvene adduct (λ = 301 nm, ε = 7800 M cm^{-1}) was determined using a spectrophotometer. The loading of the resin was determined using the following formula

$$L = A_{301}/(78 \times M)$$

with L being the resin loading, A_{301} the absorbance at 301 nm, and M the weight of the sample. The loadings obtained ranged from 0.35-0.55 mmol/g.

Iterative peptide synthesis: Peptides were typically synthesized on a 0.05-0.1 mmol scale. After an initial washing step (3 x 5 mL DMF) peptides were assembled following a cycle of deprotection and coupling steps.

Deprotection: The resin was treated with 20% piperidine/DMF (6 mL, 1 x 2 min., 2 x 8 min.) and washed with DMF (3 x 6 mL), DCM (2 x 6 mL), and DMF (3 x 6 mL). An aliquot at the end of every deprotection was taken and the absorption at 301 nm measured on the NanoDrop™ to ensure complete deprotection. Following the deprotection of the N-terminal amino acid, the resin was washed with DMF (2 x 6 mL) and DCM (5 x 6 mL) and subsequently dried under vacuum.

Coupling: Fmoc-protected amino acids (6 eq.) were pre-activated for 2 minutes in DMF (final concentration 0.5 M) following the addition of HCTU (5.8 eq.), OxymaPure® (5.8 eq.) and DIPEA (13 eq.). Preactivated Fmoc-amino acids were transferred to the resin and the resulting mixture was agitated (bubbling N_2 through the syringe) for 1 hour at room temperature. Subsequently, the resin was drained and washed with DMF (5 x 6 mL).

Cleavage and peptide isolation: A cleavage cocktail containing TFA/TIS/EDT/water (90:4:4:2 v/v/v/v, 10 mL) was added to the dried resin and incubated at room temperature for 2 hours. Subsequently, the resulting mixture was filtered, the resin washed with TFA (2 x 3 mL) and the resulting filtrate concentrated to ~0.5 mL by blowing a constant stream of N_2 over the solution. Peptides were then precipitated by the addition of ice-cold diethyl-ether (20 mL) and subsequently pelleted by centrifugation (3,000 rpm). The supernatant was carefully removed by decantation and the precipitate washed twice with ice-cold ether (20 mL) to remove organic impurities. Residual ether was removed by blowing a constant stream of N_2 over the sample and the residue subsequently dissolved in 0.1% TFA (aq.) and freeze-dried. Peptides obtained from this procedure were used without any further purification in cyclization experiments.

AVSSGGCWA-NH_2 (74.8 mg, 79% yield), *KVSSGGCWA-NH_2* (53.1 mg, 90% yield), *Ac-KVSSGGCWA-NH_2* (34 mg, 56% yield), *AVSSGGSGCWA-NH_2* (85.2 mg, 78% yield), *AVNEKHYCWA-NH_2* (131 mg, 98% yield), *AWWTNDFCACA-NH_2* (154 mg, 86%), *AWWEDRGPPCA-NH_2* (139 mg, 83%), *ARNVGLVGMCA-NH_2* (85 mg, 68%), and *AWWTNDFCA-NH_2* (175 mg, 95%) were obtained as white solids following lyophilization.

5. Chemical synthesis:

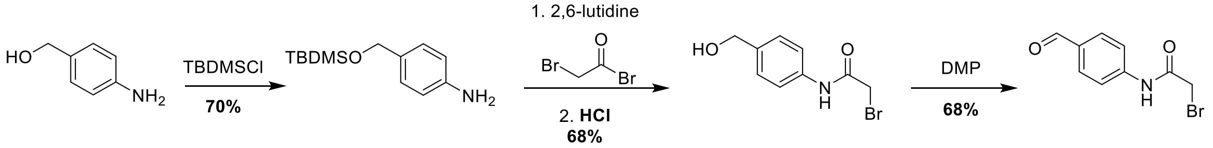

S1: Compound **S1** was synthesized as described previously.[2] 4-aminobenzyl alcohol (500 mg, 4.06 mmol), TBDMS-Cl (612 mg, 4.06 mmol), and imidazole (553 mg, 8.12 mmol), were dissolved in anhydrous DMF (7 mL). The resulting solution was stirred for 1 h at room temperature. After that, water (20 mL) was added and the product extracted with EtOAc (3 x 50 mL). The combined organic layers were washed with saturated $NaHCO_3$ (aq.), water, and brine, and dried over $MgSO_4$. The solvent was evaporated and the residue purified by silica column chromatography (10-30% EtOAc in heptane) to yield 672 mg (70%) of TBDMS ether **S1** as a pale-yellow oil. ^{1}H-NMR (400 MHz, Chloroform-d) δ 7.11 (d, J = 8.3 Hz, 2H), 6.66 (d, J = 8.4 Hz, 2H), 4.62 (s, 2H), 3.61 (s, 2H), 0.93 (s, 9H), 0.08 (s, 6H). ^{13}C-NMR (101 MHz, cdcl3) δ 145.46, 131.70, 127.83, 115.12, 65.14, 26.14, 18.58, -5.00. HRMS (ESI-TOF) m/z 238.1625 (238.1622 calc. for $C_{13}H_{24}NOSi$, $[M+H]^+$).

S2: 2,6-lutidine (0.788 mL, 6.8 mmol) and bromoacetyl bromide (0.329 mL, 3.80 mmol) were added under N_2 atmosphere at 4 °C to a solution of TBDMS protected 4-aminobenzyl alcohol (**S1**, 600 mg, 2.52 mmol) in dry DCM (6 mL). After stirring for one hour, the reaction was quenched by addition of 1 M HCl (aq., 10 mL). DCM was removed under vacuum and MeOH (20 mL) added. The reaction was then stirred for 30 min to allow for the removal of the TBDMS protecting group. Excess methanol was removed and the resulting water phase extracted three times with EtOAc (50 mL). The organic phases were combined, washed with water and brine and subsequently dried over $MgSO_4$. EtOAc was removed in vacuo and the off-white product repeatedly washed with ice-cold DCM (3x 3 mL), to yield 200 mg **S2** (68%) as a white solid. ^{1}H-NMR (400 MHz, Methanol-d4) δ 7.55 (d, J = 8.1 Hz, 2H), 7.33 (d, J = 8.0 Hz, 2H), 4.57 (s,

2H), 3.97 (s, 2H). ^{13}C-NMR (101 MHz, cd3od) δ 167.64, 139.13, 138.54, 128.64, 121.13, 64.76, 29.68. HRMS (ESI-TOF) m/z 242.9895 and 244.9868 (242.9889 and 244.9869 calc. for $C_9H_{11}BrNO_2$, $[M+H]^+$) and 225.9866 (225.9867 calc. for $C_9H_9BrNO^+$ $[M-H_2O]^+$).

2: The bi-functional scaffold **S3** was obtained by adapting a procedure of Toshima *et al.*[3] Dess-Martin periodinane (383 mg, 0.901 mmol) was added under N_2 atmosphere at room temperature to a solution of the acetamide compound **S2** (200 mg, 0.819 mmol) in acetonitrile (5 mL). The resulting solution was stirred for one hour or until TLC indicated full conversion. Subsequently, any precipitates formed during the reaction were filtered and washed with acetonitrile. The filtrate was then concentrated and purified by silica column chromatography (20-50% EtOAc in heptane), to give 128 mg **2** (68% yield) as white/translucent crystals. ^{1}H-NMR (400 MHz, DMSO-d6) δ 10.79 (s, 1H), 9.90 (s, 1H), 7.89 (d, J = 8.6 Hz, 2H), 7.80 (d, J = 8.6 Hz, 2H), 4.13 (s, 2H). ^{13}C-NMR (101 MHz, dmso) δ 191.64, 165.55, 144.04, 131.77, 130.89, 118.97, 30.27. HRMS (ESI-TOF) m/z 239.9670 and 241.9650 (239.9655 and 241.9634 calc. for $C_9H_9BrNO_2$, $[M-H]^-$).

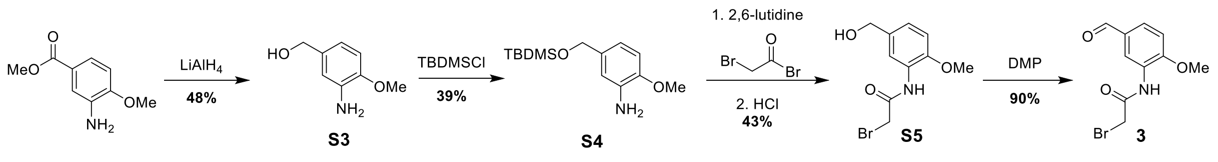

S3: To obtain amino alcohol **S3** a previously-described procedure by Toshima et al. was adapted.[3] $LiAlH_4$ (314 mg, 8.3 mmol) was added to a solution of methyl 3-amino-4-methoxybenzoate (1.0 g, 5.5 mmol) in dry THF (20.0 mL) under N_2 atmosphere in an ice bath. The reaction mixture was stirred for 1 hour at room temperature or until TLC analysis (30% EtOAc in heptane) confirmed full conversion. The reaction was quenched by the slow addition of MeOH (15 mL) and subsequently EtOAc (15 mL) was added. After the solution was stirred for 15 min at room temperature, the solvents were removed in vacuo. Water (25 mL) was added

and the product was extracted with EtOAc (60 mL × 3). The extracts were washed with brine (60 mL), dried over anhydrous Na_2SO_4, filtered, and concentrated in vacuo. The residue was purified by silica column chromatography (45-100% EtOAc in heptane) to yield **S3** (444 mg, 2.90 mmol, 48%) as a white solid. ^{1}H-NMR (400 MHz, Chloroform-d) δ 6.86 – 6.60 (m, 3H), 4.53 (s, 2H), 3.85 (s, 3H).; ^{13}C -NMR (101 MHz, Chloroform-d) δ 147.11, 135.71, 133.95, 117.41, 114.20, 110.43, 65.47, 55.72.; HRMS (ESI-TOF) m/z 154.0860 (154.0863 calc. for $C_8H_{12}NO_2$, $[M+H]^+$).

S4: TBDMS-protected compound **S4** was prepared as described for **S1** and was isolated as off-white solid (275 mg, 39%) by silica column chromatography (45%-100% EtOAc in heptane). ^{1}H-NMR (400 MHz, DMSO-d6): δ 6.72 (d, J = 8.1 Hz, 1H), 6.59 (d, J = 2.1 Hz, 1H), 6.45 (dd, J = 8.1, 2.1 Hz, 1H), 4.66 (s, 2H), 4.50 (s, 2H), 3.73 (s, 3H), 0.88 (s, 9H), 0.05 (s, 6H); ^{13}C-NMR (101 MHz, DMSO-d6): δ 145.48, 137.32, 133.42, 114.18, 112.24, 110.09, 64.59, 55.29, 26.23, 18.03, -5.74; HRMS (ESI-TOF) m/z 268.1727 (268.1727 calc. for $C_{14}H_{26}NO_2Si$, $[M+H]^+$).

S5: Following the procedure as described for compound **S2**, acetamide **S5** was obtained as brown solid (85 mg, 43%). ^{1}H-NMR (400 MHz, Methanol-d4) δ 8.05 (d, J = 2.2 Hz, 1H), 7.14 (dd, J = 8.4, 2.1 Hz, 1H), 7.00 (d, J = 8.4 Hz, 1H), 4.53 (s, 2H), 4.10 (s, 2H), 3.89 (s, 3H).; ^{13}C-NMR (101 MHz, Methanol-d4) δ 167.36, 150.60, 134.97, 127.66, 125.34, 121.83, 111.69, 64.97, 56.48, 29.80.; HRMS (ESI-TOF) m/z 274.0070 (274.0073 calc. for $C_{10}H_{13}BrNO_3$, $[M+H]^+$) and 255.9967 (255.9973 calc. for $C_{10}H_{11}BrNO_2$, $[M-H_2O]^+$).

3: Following the procedure for **2**, the bifunctional scaffold **3** was obtained as a white solid (54 mg, 0.20 mmol, 90%). ^{1}H-NMR (400 MHz, DMSO-d6) δ 9.86 (s, 1H), 9.81 (s, 1H), 8.54 (s,

1H), 7.73 (d, J = 10.2 Hz, 1H), 7.29 (d, J = 8.5 Hz, 1H), 4.23 (s, 2H), 3.97 (s, 3H).; ^{13}C-NMR (151 MHz, DMSO-d6) δ 191.51, 165.45, 154.30, 129.25, 128.54, 127.42, 120.96, 111.39, 56.41, 30.15; HRMS (ESI-TOF) m/z 270.9837 and 272.9816 (270.9839 and 272.9818 calc. For $C_{10}H_{10}BrNO_3$, $[M+H]^+$).

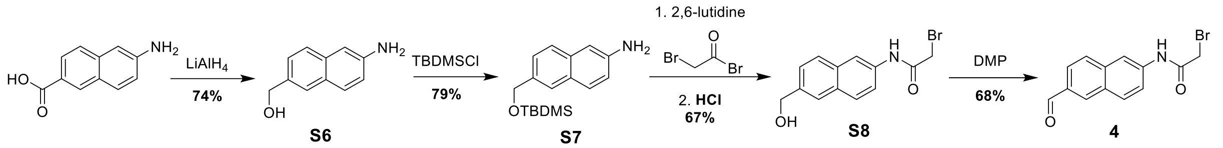

S6: (6-Aminonaphthyl)methanol (**S6**) was obtained following a procedure described by Sergeyev *et al.*[4] $LiAlH_4$ (1 M, 12.82 mmol) was added dropwise at 0 °C to a solution of (6-aminonaphthyl)carboxylic acid (800 mg, 4.27 mmol) in dry THF (20 mL). After the addition was completed, the mixture was stirred for 1 h at room temperature and then heated to reflux for an additional 2 h. The mixture was allowed to reach room temperature and quenched by the dropwise addition of water (2 mL). The inorganic precipitate was filtered and the filtrate dried over $MgSO_4$ and concentrated to give **S6** (549 mg, 74%) as a slightly brownish solid. ^{1}H-NMR (400 MHz, dmso) δ 7.57 – 7.49 (m, 2H), 7.44 (d, J = 8.4 Hz, 1H), 7.23 (dd, J = 8.4, 1.7 Hz, 1H), 6.90 (dd, J = 8.7, 2.2 Hz, 1H), 6.78 (d, J = 2.2 Hz, 1H), 5.26 (s, 2H), 5.08 (t, J = 5.7 Hz, 1H), 4.52 (d, J = 5.6 Hz, 2H). ^{13}C-NMR (101 MHz, dmso) δ 146.33, 134.89, 133.60, 128.34, 126.10, 125.56, 124.95, 122.84, 118.84, 105.96, 63.27. HRMS (ESI-TOF) m/z 174.0916 (174.0913 calc. for $C_{11}H_{12}NO$, $[M+H]^+$).

S7: TBDMS-protected compound **S7** was prepared as described for **S1** and obtained as off-white solid (619 mg, 79%). ^{1}H-NMR (400 MHz, dmso) δ 7.58 – 7.49 (m, 2H), 7.46 (d, J = 8.5 Hz, 1H), 7.22 (dd, J = 8.4, 1.7 Hz, 1H), 6.91 (dd, J = 8.7, 2.2 Hz, 1H), 6.79 (d, J = 2.2 Hz, 1H), 5.30 (s, 2H), 4.73 (s, 2H), 0.90 (s, 9H), 0.08 (s, 6H). ^{13}C-NMR (101 MHz, dmso) δ 146.51,

134.24, 133.43, 128.39, 125.98, 125.10, 124.58, 118.48, 105.89, 64.76, 25.86, 18.06, -5.15. HRMS (ESI-TOF) m/z 288.1782 (288.1778 calc. for $C_{17}H_{26}NOSi$, $[M+H]^+$).

S8: Following the procedure as described for compound **S2**, acetamide **S8** was obtained as a yellow solid 200 mg (67%). ^{1}H-NMR (400 MHz, dmso) δ 10.55 (s, 1H), 8.26 (d, J = 2.1 Hz, 1H), 7.86 (d, J = 8.8 Hz, 1H), 7.80 (d, J = 8.5 Hz, 1H), 7.76 (s, 1H), 7.55 (dd, J = 8.8, 2.1 Hz, 1H), 7.44 (dd, J = 8.5, 1.7 Hz, 1H), 4.63 (s, 2H), 4.09 (s, 2H). ^{13}C-NMR (101 MHz, dmso) δ 164.96, 139.19, 135.78, 132.38, 129.88, 128.37, 127.23, 125.92, 124.18, 119.77, 115.49, 62.95, 30.45. HRMS (ESI-TOF) m/z 293.0048 and 295.0028 (293.0046 and 295.0026 calc. for $C_{13}H_{12}BrNO_2$, $[M+H]^+$) and 276.0022 (276.0024 calc. for $C_{13}H_{11}BrNO$, $[M-H_2O]^+$).

4: Following the procedure for **2**, the bifunctional scaffold **4** was obtained as an off-white solid 102 mg (68%). ^{1}H-NMR (400 MHz, dmso) δ 10.79 (s, 1H), 10.11 (s, 1H), 8.51 (d, J = 1.6 Hz, 1H), 8.41 (d, J = 2.1 Hz, 1H), 8.14 (d, J = 8.8 Hz, 1H), 8.00 (d, J = 8.6 Hz, 1H), 7.86 (dd, J = 8.6, 1.6 Hz, 1H), 7.71 (dd, J = 8.8, 2.1 Hz, 1H), 4.13 (s, 2H). ^{13}C-NMR (101 MHz, dmso) δ 192.67, 165.45, 139.13, 136.64, 134.15, 132.89, 130.65, 129.02, 128.52, 123.05, 120.69, 115.26, 30.35. HRMS (ESI-TOF) m/z 289.98242 and 291.9803 (289.9811 and 291.9791 calc. For $C_{13}H_{19}BrNO_2$, $[M-H]^-$).

6. Peptide cyclization reactions

Cyclization reactions of model peptides (pep1-5) with cyclization moieties 1-4: Crude lyophilized peptide (25 µL of a 20 mM stock solution in 0.1% TFA), ddH_2O (187.5 µL), acetonitrile (100 µL), TCEP (37.5 µL of a 4 mM stock in ddH_2O), and cyclization units (100 µL of 15 mM stock solutions in acetonitrile) were added sequentially to MOPS buffer (500 µL, 200 mM, pH 6.5). The resulting solutions were incubated at 30 °C for 3 hours or until UPLC-MS analysis indicated >95% conversion to the modified peptide. At this stage $NaBH_3CN$ (20 µL of a 200 mM stock solution in ddH_2O) was added and the reaction was incubated overnight at 4 °C. The next morning two more portions of $NaBH_3CN$ were added in three-hour intervals (final concentration 10 mM in the reactions) and the resulting reaction mixtures were analyzed by UPLC-MS.

Modification and cyclization reactions of peptide hits SAV1 and SAV2 with scaffolds 3 and S5, and SAV3 with scaffolds 4 and S8: Crude lyophilized peptide (1 eq., ~15 mg, 0.01 mmol) and TCEP (0.5 eq., 1.25 mg, 0.005 mmol) were dissolved in a total volume of 10-20 mL of ddH_2O (40 mM Na_2HPO_4/ NaH_2PO_4, pH 6.7, 40-60% DMSO, 5% ACN). Cyclization units (1.6-2.6 eq., 0.016-0.026 mmol) were added and the resulting solutions were incubated at 30 °C for 6 hours or until UPLC-MS analysis indicated >95% conversion to the modified peptide. To proceed to the cyclic peptide, at this stage 5% TFA in MQ was added to lower the pH of the reactions to pH 5. $NaBH_3CN$ (50-100 µL of a 200 mM stock solution in ddH_2O) was next added and the reactions were put at 4 °C. Over the course of 3 days, multiple batches of freshly prepared $NaBH_3CN$ (50-100 µL of a 200 mM stock solution in ddH_2O) were added to the reactions up to a total concentration of 15 mM. UPLC-MS analyses confirmed >80% conversion and the peptides were lyophilized. Next, they were dissolved in 2-3 mL of 70/30 DMSO/ 0.1% TFA in MQ and purified using HPLC-prep (see section 3 and S14).

Cyclization for 1D and 2D-NMR spectroscopy studies: Crude, lyophilized peptide (1 eq., 3-4 mg, ~3 mmol), 100-110 µL acetonitrile, cyclization unit **1** (1.05 eq., ~3.15 mmol, 90-100 µL of a 31 mM stock solution in acetonitrile), and ddH_2O (600 µL) were added to phosphate buffer (100 µL, 200 mM, pH 6.5). The resulting solutions were incubated at 30 °C for 3 hours or until >95% of the starting peptide was modified as indicated by UPLC-MS analysis. At this stage $NaBH_3CN$ (40 µL of a 50 mM stock solution in H_2O) was added and the reaction was incubated overnight at 4 °C. The next day, three more portions of $NaBH_3CN$ (3x 20 µL of a 50 mM stock solution in H_2O) were added in four-hour intervals (final concentration 5 mM). After UPLC-MS analysis indicated full conversion to the reduced product, the reactions mixtures were lyophilized. The resulting, crude cyclic peptides were then dissolved in 550 µL of an NMR solvent consisting of: 50 µL D_2O, 100-200 µL 0.1% TFA in H_2O (pH ~4-5), and 300-400 µL H_2O. To obtain an NMR sample of the modified peptides, reaction mixtures were lyophilized following the initial modification step.

7. Molecular Biology

The gene encoding for the fusion protein pep-TEV-D1D2 was obtained via the following steps: (1) construction of AGSSGGC-D1D2 and its insertion into pET21b(+); (2) insertion of the TEV cleave site to obtain AGSSGGC-TEV-D1D2; and (3) site-directed mutagenesis of AGSSGC-TEV-D1D2 into AVSSGGC-TEV-D1D2 (= pep-TEV-D1D2).

Construction of pET21b(+)-D1D2 and pET21b(+)-AGSSGGC-D1D2: The gene encoding for the disulfide-free D1D2 domains as well as AGSSGGC-D1D2 were amplified from fdg3p0ss21[5] using the primers NdeI_D1D2_fw or NdeI_pep_D1D2_fw and XhoI_D1D2_rv, which also installed appropriate restriction enzyme sites for restriction digest (see Section 13) using the following PCR protocol: (1) initial denaturation 98 ˚C for 1 min, (2) 30 cycles of denaturation at 98 ˚C for 10 s, annealing at 54 ˚C for 30 s and extension at 72 ˚C for 30 s; (3) a final extension at 72 ˚C for 10 min. Successful amplification as well as the size of the PCR products was verified by agarose gel (1%). The remaining template was removed by DpnI digestion at 37 ˚C for 16 hours. Following PCR purification, the resulting constructs were cloned into pET21b(+) following standard restriction enzyme cloning procedures (NdeI and XhoI). Ligation mixtures were transformed into chemically-competed *E. coli* NEB10β cells and successful transformants identified after overnight incubation on selective LB agar plates containing ampicillin. Single colonies were picked and the identity of the inserts confirmed by Sanger sequencing to give the plasmids pET21b(+)-D1D2 and pET21b(+)-AGSSGGC-D1D2.

Construction of pET21b(+)-AGSSGGC-TEV-D1D2: The TEV cleavage site in the AGGSSGGC-D1D2 gene was installed by overlap extension PCR combining fragments obtained following PCR amplification of pET21b(+)-AGSSGGC-D1D2 with the primer pairs TEV_fw1/rv1 and TEV_fw2/ rv2. The two fragments were joined using TEV_fw1 and TEV_rv2 using the following touchdown PCR protocol: (1) initial denaturation 98 ˚C for 2 min,

(2) 16 cycles of denaturation at 98 ˚C for 10 s, annealing at 64-56 ˚C for 30 s and extension at 72 ˚C for 30 s; (3) 14 cycles of denaturation at 98 ˚C for 10 s, annealing at 56 ˚C for 30 s and extension at 72 ˚C for 30 s; (4) final extension at 72 ˚C for 5 min. The resulting construct AGSSGGC-TEV-D1D2 was inserted into pET21b(+) and transformed into chemically-competent *E.coli* NEB10β cells as described before. The identity of the insert in the resulting pET21b(+)AGSSGGC-TEV-D1D2 was confirmed by sequencing.

Construction of pET21b(+)pep-TEV-D1D2: pET21b(+)-pept-TEV-D1D2 was obtained by QuikChange site-directed mutagenesis of pET21b(+)-AGSSGGC-TEV-D1D2 using SDM_Val_fw and SDM_Val_rv as primers. Following DpnI digestion and PCR cleanup the resulting PCR product was transformed into chemically-competent *E.coli* NEB10β cells and the identity of the insert confirmed by sequencing. Lastly, the resulting plasmid pET21b(+)pep-TEV-D1D2 as well as the previously obtained pET21b(+)-D1D2 were transformed into chemically-competent *E. coli* BL21 DE3 cells.

Protein production and purification: Flasks containing 500 mL LB-medium with 100 µg/mL ampicillin were inoculated with 2.5 mL of a densely grown overnight culture of *E. coli* BL21(DE3) cells harboring plasmids pET21b(+)-D1D2 or pET21b(+)-pep-TEV-D1D2. Cells were incubated at 37 °C while shaking (135 rpm) until an OD_{600} of 0.4 – 0.6 was reached (approx. 4 h). At this stage, gene expression was induced by addition of isopropyl β-D-1-thiogalactopyranoside (IPTG, final concentration 1 mM) and incubation was continued for 16 hours at 30 °C. Cells were harvested by centrifugation (6,000 rpm, JLA10.500 Beckman, 20 min, 4 °C) and the supernatant discarded. The cell pellet was resuspended in 20 mL washing buffer (50 mM NaH_2PO_4, 150 mM NaCl, pH 7.5) containing 1 mg/mL egg white lysozyme and a tablet of protease inhibitor cocktail (cOmplete^{TM} Mini, Roche). Cell suspension was subsequently lysed by sonication (70% (200 W) for 10 min, 15 sec on, 10 sec off). The lysed cells were centrifuged to remove cell debris (12,000 rpm, JA-17.5, 60 min., 4 °C), the cleared

lysate was purified by Ni-NTA chromatography (GE Healthcare) according to the supplier's specifications. His-tagged D1D2 variants were eluted with elution buffer (50 mM NaH_2PO_4, 150 mM NaCl, 250 mM imidazole, pH 7.5). Protein containing fractions, as judged by SDS-PAGE, were pooled and concentrated using filtration tubes with a cut-off of 15 kDa (4,000 rpm, JLA-17.5, 60 min, 4 °C). Phosphate buffer (50 mM NaH_2PO_4, 150 mM NaCl, pH 7.5) was repeatedly added to remove the imidazole. Protein aliquots (~1 mL) were stored at 4 °C or -20 °C until further use. Concentrations were determined by measuring the absorbance at 280 nm on a NanoDrop 2000 (Thermoscientific) spectrophotometer with calculated extinction coefficients (49,850 M^{-1} cm^{-1} for D1D2 and TEV-D1D2). Yields were 137 mg for D1D2 and 97 mg pep-TEV-D1D2, per liter culture.

Construction of fd_pep -D1D2: The gene encoding for pep-TEV-D1D2 was amplified from the corresponding pET21b(+) plasmid using the primers SfiI_fd_fw and SfiI_fd_rv according to the following protocol: 1) initial denaturation 98 ˚C for 2 min, (2) 40 cycles of denaturation at 98 ˚C for 20 s, annealing at 58 ˚C for 45 s and extension at 72 ˚C for 1 min; (3) a final extension at 72 ˚C for 7 min. Following DpnI digestion at 37 ˚C for 16 hours and PCR purification, the resulting constructs were digested with SfiI for 5 h at 50 ˚C. In parallel, the recipient fd phage vector fd0D1D2 was linearized by incubation with EcoRI for 2 h at 37 ˚C and subsequently digested by SfiI at 50 ˚C for 3 h. The resulting products from both SfiI digestions were ligated using T4 ligase at 37 ˚C for 1 h and the crude ligation mixture transformed into chemically-competed TG1 *E. coli* cells. Successful transformants were identified after overnight incubation on selective 2xYT agar plates containing chloramphenicol. Single colonies were picked and the identity of the inserts confirmed by Sanger sequencing to give fd_pep-D1D2.

8. Protein cyclization & TEV cleavage

Cyclization of pep-TEV-D1D2 with cyclization moieties 1-4: TCEP (50 µL from a 10 mM stock in ddH_2O), and ddH_2O (80 µL) were added to 870 µL of a solution containing D1D2 or pep-TEV-D1D2 (92 µM stock solution in 50 mM NaH_2PO_4, 150 mM NaCl, pH 7.5). The resulting reaction was incubated at 30 °C for 30 min to allow for reduction of any disulfides formed during phage production. The sample was split evenly into 2x500 µL fractions, which were eluted over a PD MiniTrap™ column (GE Healthcare, 2.1 mL of Sephadex™ G-25 resin) with 1 mL MOPS buffer (100 mM, pH 7). At this point an aliquot of the sample was removed for UPLC-MS analysis. Next, cyclization units **1-4** (50 µL from a 5 mM stock solution in acetonitrile) were added to 500 µL of a solution containing reduced protein samples. After 5 hours, UPLC-MS analysis confirmed complete modification for pep-TEV-D1D2, after which the modified proteins were eluted over a new PD MiniTrap™ column with 1 mL MES buffer (50 mM, pH 6). At this stage $NaBH_3CN$ (10 µL from a 100 mM stock in ddH_2O) was added and the reaction was incubated overnight at 4 °C. The next day, four more portions of $NaBH_3CN$ were added (4 x 10 µL from a 100 mM stock in ddH_2O) in three-hour intervals (final concentration 5 mM) and the reactions were incubated for an additional 16 hours at 4 °C. The reaction mixtures were then subjected to UPLC-MS analysis to determine the conversion to cyclized pep-TEV-D1D2.

TEV cleavage of AVSSGGC-TEV-D1D2 after cyclization: TEV protease (1 µL, *SigmaAldrich*) was added to 50 µL of cyclized protein sample obtained from the procedure described above. The reaction mixture was incubated at 30 °C for 3 h before an additional 1 µL aliquot of TEV protease was added. The resulting reaction mixtures were incubated at 4 °C overnight and subjected to UPLC-MS analysis the next day.

9. Phage production and infectivity studies

Production fd_pep-D1D2 phages: Flasks containing 500 mL 2xYT-medium with 30 µg/mL chloramphenicol were inoculated with 2.5 mL of a densely grown overnight culture of *E.coli* TG1 cells harboring the fd_pep-D1D2 plasmid and incubated overnight at 30 °C at 135 rpm. The cultures were subsequently centrifuged (6,000 rpm, JLA10.500 Beckman, 20 min, 4 °C) and the phage-containing supernatant was decanted to new centrifuges bottles. From the phage solution, a 50 µl ‘supernatant’ aliquot was taken and stored at 4 °C for infectivity studies (see below). A solution of ice-cold PEG-NaCl solution (125 mL, 20% PEG-6000 (w/v), 2.5 M NaCl) was added to the supernatant and the resulting mixture incubated on ice for 30 min. Phages that precipitated during this time were pelleted by centrifugation (9.500 rpm, JLA10.500 Beckman, 45 min, 4 °C). The supernatant was carefully decanted and the centrifuge bottle placed upside down on a filter paper for 2 min to remove all residual liquid. The phage pellet was then resuspended in 20 mL degassed reaction buffer (20 mM HEPES containing 5 mM EDTA at pH 8.0) and the mixture was transferred into a 50 mL Greiner tube. Remaining cell/phage debris was removed by centrifugation (4000 rpm, Eppendorf A-4-62, 15 min, 4 °C) and the supernatant carefully transferred into a new 50 mL Greiner tube. At this point a 50 µl ‘PEG precipitation’ aliquot was taken and stored at 4 °C. TCEP (final concentration 1 mM) was added to the PEG-purified phages and the resulting reaction mixture was incubated at 42 °C for 1 h. Samples were concentrated to 1 mL using Amicon® Ultra-15 (cut off of 100 kDa) centrifugation tubes. Next, reduced phages were washed three times with 12 ml ice-cold reaction buffer (20 mM HEPES, 5 mM EDTA, pH 8). Phages prone to accumulate on the filter during this procedure were resuspended by pipetting samples up and down repeatedly. After the final wash, samples were concentrated to 1 mL, transferred to a 15 mL tube and the volume adjusted with reaction buffer to 9 mL. At this point, a ‘TCEP reduction’ aliquot was taken and stored at 4 °C.

Cyclization on phages: Cyclization units **1-4** (1 mL of 0.5–5 mM stock solutions in acetonitrile) were added to phage samples obtained after TCEP reduction and the resulting reaction mixtures were incubated at 30 °C for 1 h. The samples were subsequently concentrated to 1 mL and washed three times with reduction buffer (50 mM MES, pH 6) as described above. Following the final wash, samples were concentrated to 1 mL, transferred to a 15 mL tube and the volume adjusted with reduction buffer to 9 mL. At this point, a 50 'Cys-modification' aliquot was taken and stored at 4 °C. $NaBH_3CN$ (2 x 200 µL from 25 mM stock) was added to the phage samples and the reaction was incubated overnight at 4 °C. The next morning, three more batches of $NaBH_3CN$ (3 x 200 µL from 25 mM stock) were added in 2 h time intervals to reach a final concentration of 2.5 mM in the reaction mixture. Samples were subsequently concentrated to 1 mL and washed three times with reduction buffer (50 mM MES, pH 6) as described above. Following the final wash, samples were concentrated to 1 mL, transferred to a 15 mL tube and the volume adjusted with reduction buffer to 10 mL. At this point a 50 µl '$NaBH_3CN$ reduction' aliquot was taken and stored at 4 °C.

Infectivity studies: Stored 50 µL aliquots of the 'supernatant', 'PEG-purifaction', 'TCEP reduction', 'cyc-modification', and '$NaBH_3CN$ reduction' were used to determine the phage infectivity after every handling step. For each sample, seven 10-fold dilutions in 2xYT were prepared. Aliquots (20 µL) of samples corresponding to 10^{-5}, 10^{-6}, and 10^{-7} dilutions were added to 180 µL of *E.coli* TG1 cells growing in the mid-log phase (OD_{600} ~0.4). Phages in the samples were allowed to infect cells at 37 °C at 135 rpm. for 90 min. An aliquot (50 µL) of each dilution was then plated onto 2xYT/chloramphenicol agar plates and incubated overnight at 37 °C. The next day, colonies on the plates were counted and the number of infectious phages was calculated by adjusting for the corresponding dilution factors.

10. Library construction

The phage library displaying peptide variants of A(X)$_8$C, where X is any amino acid, was constructed by adapting a previously established procedure.[6] Library construction consisted of the following steps: (1) PCR amplification of the genetic fragment GGSG-D1D2-*SfiI*; (2) construction of the library *SfiI*-A(X)$_8$C-GSGG-D1D2-*SfiI*; and (3) cloning of the library *SfiI*-A(X)8C-GGSG-D1D2-*SfiI* into the recipient vector fd0D1D2 and transformation of the plasmid library into *E. coli* TG1 cells.

Preparation of GSGG-D1D2-*SfiI*: In an initial PCR, the gene encoding for the disulfide-free D1D2 domains was amplified from fdg3p0ss21[6] using the primers GGSG_fd_fw and fd_SfiI_rv. The PCR, which also appended a GGSG-linker and installed the downstream SfiI restriction site, was performed using the following protocol: (1) initial denaturation 95 ˚C for 2 min, (2) 30 cycles of denaturation at 95 ˚C for 50 s, annealing at 58 ˚C for 50 s and extension at 72 ˚C for 2.5 min; (3) a final extension at 72 ˚C for 10 min. Successful amplification as well as the size of the PCR products was verified by gel electrophoresis (1%). The remaining template was digested with DpnI at 37 ˚C for 16 hours. Following PCR purification, the resulting fragment was used for the construction of the library.

Construction of the fragment library *SfiI*-A(X)$_8$C-GGSG-D1D2-*SfiI*: The sequence encoding for the randomized peptide A(X)$_8$C, as well as another *SfiI* restriction site were installed in a second PCR amplification. To a total of 100 ng of GGSG-D1D2-SfiI PCR product was added nuclease-free water (to obtain 0.5 mL final volume), 500 nM degenerate primer SfiI_AX8C_fw (final conc.), 500 nM fd_SfiI_rv (final conc.), 250 µM dNTP mix (final conc.) and 50 µL 10x Taq buffer. After mixing, the PCR master mix was distributed over ten 0.1 mL tubes on ice and 1 µL Taq polymerase (5 units) was added to each tube. The fragment library was obtained using the following PCR protocol: (1) initial denaturation 95 ˚C for 2 min, (2) 28 cycles of denaturation at 95 ˚C for 50 s, annealing at 60 ˚C for 50 s and extension at 72 ˚C for

2.5 min; (3) final extension at 72 ˚C for 7 min. The PCR product was purified by gel electrophoresis (1% agarose) followed by gel extraction (QIAGEN). The fragment library was eluted in Tris-HCl buffer (pH 8.5 to ensure compatibility with the buffer system used for *SfiI* digestion).

Cloning of *SfiI*-A(X)$_8$C-GGSG-D1D2-*SfiI* into fd0D1D2: Purified PCR product SfiI-A(X)8C-GGSG-D1D2-SfiI (27 µg) was digested in 300 µL Tris-HCl 5 mM, pH 8.5 (final volume) with restriction enzyme SfiI (12 µL, 240 units) for 16 h at 50 °C. In parallel, recipient vector fd0D1D2 (25 µg) was first linearized using EcoRI (8 µL, 160 units) for 4 h at 37 °C, followed by inactivation at 65 °C for 20 min, and subsequent SfiI digestion (8 µL, 160 units) for 16 h at 50 °C. Next, the linearized digested vector was purified by extraction from agarose gel (1%) using a gel extraction kit and digested PCR product was purified with a PCR purification kit. DNA was eluted with Tris-HCl (5 mM, pH 8.5). Next, digested vector (4 µg) was ligated with digested insert (1.25 µg; 3-fold molar excess) in 450 µL ligation mixture containing nuclease-free water (to a volume of 450 µL), 45 µL 10x ligation buffer containing ATP, and T4 ligase (22.5 µl; 112.5 Weiss units). The reaction was mixed and incubated for 24 h at 16 °C. The ligase was inactivated by incubation at 65 °C for 15 min. and the ligation mixture desalted using a PCR purification kit.

The desalted A(X)$_8$C-library was then transformed into electrocompetent *E. coli* TG1 by electroporation. For this, 300 µL of electrocompetent TG1 cells were mixed with 27 µL ligated DNA (99 ng/ µL) in three cuvettes (100 µL/ cuvette). The mixture was incubated for 5 minutes on ice, before electroporation was performed with MicroPulser™ Electroporation Apparatus (BIORAD) according to the supplier’s instructions. Immediately after electroporation, 900 µL SOC medium at 37 °C was added to the cells and they were incubated for 1 h (37 °C, 135 rpm). To assess the size of the library, a 20 µL aliquot of transformed cells was taken and a series of 10-fold dilutions were plated on 2xYT/chloramphenicol agar plates. The remaining mixture

was plated on large 2xYT/chloramphenicol agar plates and incubated overnight at 37 °C. The next day, colonies were counted on the dilution plates and cells were harvested from the large plates with 4 mL 2x YT per plate. To the harvested cells, glycerol was added to 20% and aliquots of the library (0.5 mL) were stored at -80 °C until further use.

11. Phage selections

Production fd_A(X)$_8$C-D1D2 phages: Flasks containing 500 mL 2xYT-medium with 30 µg/mL chloramphenicol were inoculated with *E. coli* TG1 glycerol stock harboring the A(X)$_8$C library to an OD_{600} of 0.1. After incubation overnight (30 °C at 200 rpm) phage particles were purified and subsequently all cysteines reduced with TCEP as described in **Section 9**.

Negative selection: For every 500 mL 2x YT starting culture, 50 µL of streptavidin coated magnetic beads (New England BioLabs) were transferred to a 1.5 mL Eppendorf tube and placed in a magnetic rack (NEB). The supernatant was removed and the beads were washed 3 times with 1 mL of binding buffer (10 mM Tris-Cl pH 7.4, 150 mM NaCl, 10 mM $MgCl_2$, 1 mM $CaCl_2$). After the final wash, the beads were resuspended in 300 µL binding buffer, complemented with 150 µL blocking buffer (binding buffer, 0.3% Tween-20, 3% (w/v) BSA) and incubated on a slowly rocking benchtop shaker for 30 min at room temperature. After that, the beads were washed with 1 mL binding buffer and added to the reduced phages in reduction buffer (2 mL) in 15 mL falcon tubes. The phage-bead suspension was incubated for 30 min at room temperature before being placed back into a magnetic rack. The supernatant was removed and used for the following cyclization on phages (vide infra). In parallel, the remaining magnetic beads were washed twice with 1 mL binding buffer, replacing the Eppendorf tubes after every wash to eliminate phages binding to plastic. Linear peptides binding to streptavidin were next eluted by resuspending the beads in 100 µL elution buffer (50 mM glycine-HCl, pH 2.2) for 5 min before placing the tube(s) back into the magnet and transferring the supernatant into new tubes containing 50 µL neutralization buffer (1 M Tris-Cl, pH 8). The so-obtained 'negative selection' sample(s) is/ are stored at 4 °C.

Cyclization on phages: The supernatant recovered from the negative selection step was concentrated to 1 mL using Amicon® Ultra-15 (cut off of 100 kDa) centrifugation tubes. Next, reduced phages were washed three times with 12 ml ice-cold reaction buffer (20 mM HEPES,

5 mM EDTA, pH 8). Phages prone to accumulate on the filter during this procedure were resuspended by pipetting samples up and down repeatedly. After the final wash, samples were concentrated to 1 mL, transferred to a 15 mL tube and the volume adjusted with reaction buffer to 4.5 mL.

Cyclization units **3** or **4** (0.5 mL of 1 mM stock solutions in acetonitrile) were added to phage samples obtained after TCEP reduction and the resulting reaction mixtures were incubated at 30 °C for 1 h. Modified phages were subsequently precipitated (1.5 mL 20% PEG-6000 (w/v), 2.5 M NaCl), harvested by centrifugation (4000 rpm, Eppendorf F34-6-38, 25 min, 4 °C) and pelleted phages were re-dissolved in 4.5 mL reduction buffer (50 mM MES, pH 6). $NaBH_3CN$ (1 x 100 µL from 50 mM stock) was added to the phage sample and the reaction was incubated overnight at 4 °C. The next morning, three more batches of $NaBH_3CN$ (3 x 100 µL from 50 mM stock) were added in 2 h time intervals. The following day, a final batch (100 µL of 50 mM stock) was added to reach a final concentration of 5 mM in the reaction mixture. On the following day, samples were concentrated to 1 mL and washed three times with binding buffer as described before. Following the final wash, samples were concentrated to 2 mL and transferred to a 15 mL falcon tube.

Positive selection: Strep-coated magnetic beads (2x 50 µL) were transferred to a 1.5 mL Eppendorf tube, washed three times with 1 mL binding buffer as described previously (see negative selection) and resuspended in 300 µL binding buffer complemented with 150 µL blocking buffer per sample. The beads were incubated on a slowly rocking benchtop shaker for 30 min at room temperature. In parallel, each phage library in 2 mL binding buffer was blocked by addition of 1 mL blocking buffer and incubated for 30 min at room temperature. During the incubation, flasks containing 2x YT (50 mL per phage library) were inoculated with 500 µL of a 5 mL overnight culture of E. coli TG1 cells (37 °C at 135 rpm). Magnetic beads were added to blocked phage libraries in new 15 mL tubes and incubated on a slowly rocking benchtop

shaker for 30 min at room temperature. Next, the tubes were transferred into a magnetic rack and the supernatant removed. The beads were washed eight times with 1 mL washing buffer (binding buffer, 0.1% Tween-20) and twice with 1 mL binding buffer. During the washes, tubes were replaced at least six times to remove plastic-binding peptides as described previously. After the final wash, the beads were resuspended in 100 µL elution buffer (50 mM glycine-HCl, pH 2.2) for 5 min before placing the tube(s) back into the magnet and transferring the supernatant to new tubes containing 50 µL neutralization buffer (1 M Tris-Cl, pH 8). A 5 µL aliquot of each library was added to 45 µL 2x YT and stored at 4 °C as ‘positive selection’ sample.

Eluted phage particles (145 µL) were added to 25 mL of the E. coli TG1 culture grown to an OD_{600} of 0.4 and incubated for 90 min. at 37 °C without shaking. Subsequently, cells were pelleted by centrifugation at 4000 rpm for 8 min at 4 °C. The pellets were resuspended in 600 µL 2x YT medium and plated on three 2x YT/ chloramphenicol plates and incubated at 37 °C overnight. The next day, cells were harvested with 1 mL 2x YT per plate, glycerol was added to 20% and aliquots of 0.5 mL were stored at -80 °C for use in the next round of selection.

Infectivity studies: Stored aliquots of the ‘negative selection’ and ‘positive-selection’ samples were used to determine the number of retained phages in the different selections over 3 rounds of phage display (see workflow described in **Section 9**).

12. HABA competition binding assay

Production of SAV_1-3 displaying bacteriophages: To validate the selection of cyclic peptide binders over their linear, and modified counterparts, phages displaying the peptide sequences of the two most enriched hits **SAV_1**, **SAV_2** and **SAV_3** were panned against streptavidin either in linear, modified or cyclic peptide form. Phage titers pre- and post-streptavidin panning were determined and compared between all the samples.

In brief, 700 μL of densely grown *E. coli* TG1 overnight cultures harboring the AWWTNDFCAC-fd plasmid (**SAV_1**), AWWEDRGPPC-fd plasmid (**SAV_2**) and ARNVGLVGMC-fd plasmid (**SAV_3**) respectively, were used to inoculate 500 mL 2xYT-medium containing 30 μg/mL chloramphenicol. After incubation overnight (30 °C at 190 rpm) phage particles were purified and all cysteines were reduced with TCEP as described in **Section 9**. At this point the phage samples were split into 3 fractions of 2.5 mL. One sample was stored at 4 °C as 'linear phage' sample, the other two samples were modified with scaffold **3** (SAV_1 and SAV_2) or scaffold **4** (SAV_3) and purified as described in **Section 9**. One 'modified phage' sample was stored at 4 °C, while the other was cyclized as described previously, yielding the 'cyclized phage' sample. All samples were subsequently purified by PEG-precipitation and dissolved in 1 mL reduction buffer for consistency (50 mM MES, pH 6). Phage titers of these samples were determined (**Figure S12A**) and all samples were diluted to approximately 10^{11} colony forming units using reduction buffer. Of all samples, 50 μL 'pre-SAV' aliquots were stored at 4 °C.

Streptavidin panning: Streptavidin-coated magnetic beads (190 μL) were first washed, then resuspended in 540 μL of binding buffer, and 270 μL blocking buffer (binding buffer, 0.3% Tween-20, 3% (w/v) BSA) added as described in **Section 11**. The resulting mixtures were incubated while slowly rocking for 30 min at room temperature, after which the beads were washed with 1 mL of binding buffer. In parallel, 500 μL blocking buffer was added to 950 μL

of the previously prepared phage samples (adjusted for equal phage concentrations). The mixtures were incubated on a slowly rocking benchtop shake plate for 30 min at room temperature. Next, pre-treated magnetic beads were divided over the blocked phage samples (20 µL SAV beads per sample, corresponding to ~1.5 nmol SAV-tetramer), and the resulting mixtures were again incubated while slowly rocking for 30 min at room temperature. The tubes were placed in a magnetic rack, and the supernatant was removed. After this, the beads were washed with washing buffer (7 x 500 µL, binding buffer, 0.1% Tween-20), and the tubes were replaced two times to eliminate phages binding to plastic. After the final washes (2 x 500 µL, binding buffer), the beads were resuspended in 100 µL elution buffer (50 mM glycine-HCl, pH 2.2) for 5 min., before placing the tubes back in the magnetic rack. The supernatant was subsequently transferred to new tubes containing 50 µL neutralization buffer (1 M Tris-Cl, pH 8). The elution mixtures were then used together with all the stored 'pre-SAV' samples to determine the phage titers as described in **Section 9** (**Figs. S12B-C**).

In vitro affinity measurements: To determine the affinity of the selected peptides for Streptavidin, a competitive binding experiment was performed similarly to previously described literature procedures.[7] In brief, for the competitive binding experiment, increasing amounts of peptide (final concentration 0.01 – 500 µM in 12.5% ACN + 0.1% TFA in PBS phosphate buffered saline (PBS, 137 mM NaCl, 2.7 mM KCl, 10 mM Na_2HPO_4, 2 mM KH_2PO_4, pH = 7.4)) were titrated into a PBS solution containing fixed concentrations of HABA/ Streptavidin monomer (final concentration 1.5 mM/ 40 µM (SAV_1), 33 µM (SAV_2), 32 µM (SAV_3)). The reactions were allowed to reach equilibrium at room temperature (60 min.) and the characteristic absorbance of the HABA/ streptavidin complex at 500 nm was measured. IC50 values were obtained by fitting the average of three measurements to equation 1:

$$A_{500nm} = A_{500nm,min} + \frac{A_{500nm,max} - A_{500nm,min}}{1 + \left(\frac{[L]}{IC_{50}}\right)^{H}} \qquad \mathbf{(1)}$$

where, A500nm is the measured absorbance at unbound peptide concentration, [L], and H is the Hill coefficient (H = 1 assumed for streptavidin-binding peptides). $A_{500nm,min}$ corresponds to the value measured in presence of 1 mM biotin and $A_{500nm,max}$ corresponds to absorbance values measured in absence of the peptide.

The K_d values for SAV_1_mod, SAV_1_cycl, and SAV_2_mod, SAV_1S_mod, and SAV_1S_cycl were obtained from their respective IC_{50} values using equation 2:

$$K_{d(pept)} = \frac{IC_{50}}{1 + \frac{[L_1]}{K_{d,L1}}} \quad \mathbf{(2)}$$

where, $[L_1]$ is the concentration of HABA (1.5 mM) and $K_{d,L1}$ the K_d for the HABA-streptavidin complex (100 µM).[8]

13. Isothermal titration calorimetry

In order to determine the K_d of SAV_1_cyclic and SAV_2_aldehyde directly, isothermal titration calorimetry (ITC) measurements were performed. SAV_1_linear was included for comparison. Titrations were performed with peptides in the cell (205 μL, 21 μM) and streptavidin in the syringe (40 μL, 405 μM). The concentration of available binding sites for streptavidin was determined via the characteristic absorbance of the HABA/streptavidin complex at 500 nm. The titrations were performed by injecting one 0.5 μL injection and 24 injections of 1.6 μL for the SAV_1 measurements and 19 injections of 2 μL for SAV_2_aldehyde into the ITC cell with 120 sec. spacing. The release of heat from each injection was plotted as ITC compensating power over time (dQ/dt). The heat (Q, kcal/mol) associated with each injection was obtained by calculating the area of each peak and normalizing by the number of moles of ligand added in that injection. The heat was plotted as a function of the molar ratio of ligand/receptor. A reference titration experiment of streptavidin into buffer (Tris-buffered saline: 50 mM Tris-HCl, 120 mM NaCl, 5 mM KCl, pH 7.4) was performed. Values for K_a and the number of binding sites, N, were obtained by non-linear fitting of the binding isotherm data to a model containing a single binding site using the equations provided in the instrument manual (*Origin v.7*). Reference signals were subtracted from the SAV_1 measurements and saturation normalized to zero.

14. Primer List

**Name**	**Sequence 5' → 3'**
XhoI_D1D2_rv	GTGGTGCTCGAGAGCATTGACAGGAGGTTGAGG
NdeI_D1D2_fw	GGAATTCCATATGGCTGAAACTGTTGAAAGT
NdeI_pept-D1D2_fw	GGAATTCCATATGGCTGGTAGCAGTGGCGGTTGCGCTGAAACTGTTGAAAGTAG
TEV_fw1	AAGTGGCGAGCCCGATCT
TEV_fw2	GAAAACCTGTACTTCCAGTCGGCTGAAACTGTTGAAAGTAGTTTAGC
TEV_rv1	CGACTGGAAGTACAGGTTTTCGCAACCGCCACTGCTACC
TEV_rv2	GTGGTGCTCGAGAGCATTGAC
SDM_Val_fw	GGAGATATACATATGGCTGTTAGCAGTGGCGGTTGCG
SDM_Val_rv	CGCAACCGCCACTGCTAACAGCCATATGTATATCTCC
SfiI_fd_fw	TATGCGGCCCAGCCGGCCATGGCAGCTGTTAGCAGTGGCGGTTGCG
SfiI_fd_rv	GAAGCCATGGCCCCCGAGGCCCCGGACGGAGCATTGACAGG
GGSG_fd_fw	GGCGGTTCTGGCGCTGAAACTGTTGAAAGTAG
SfiI_AX8C_fw	TATGCGGCCCAGCCGGCCATGGCAGCG(NNK)$_8$ TGCGGCGGTTCTGGCGCTG

15. NMR SPECTRA

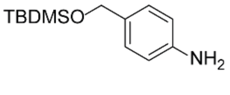

S1

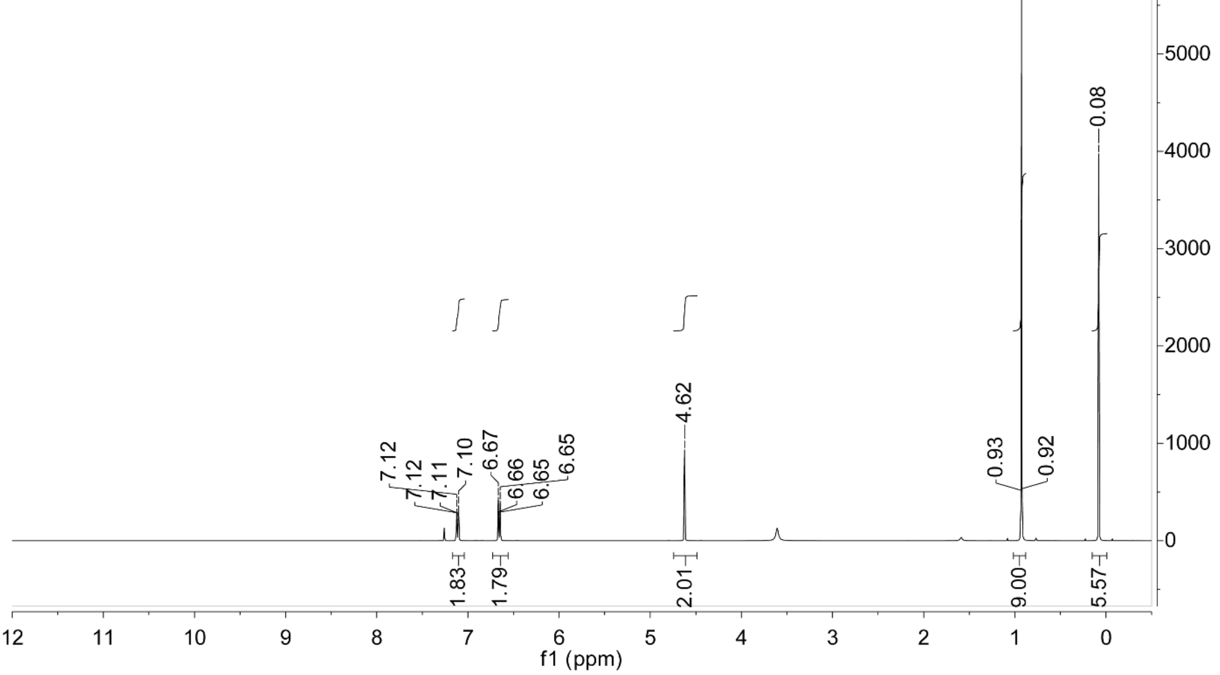

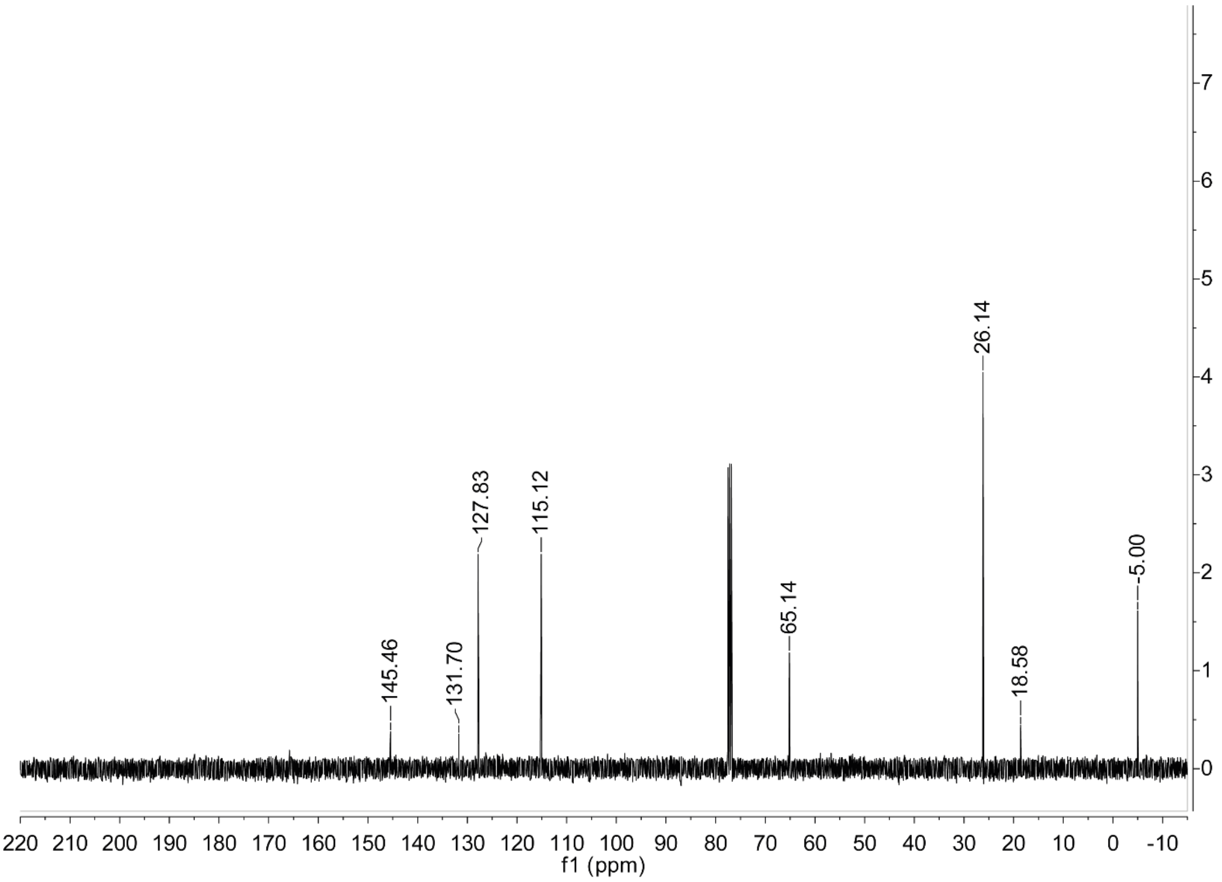

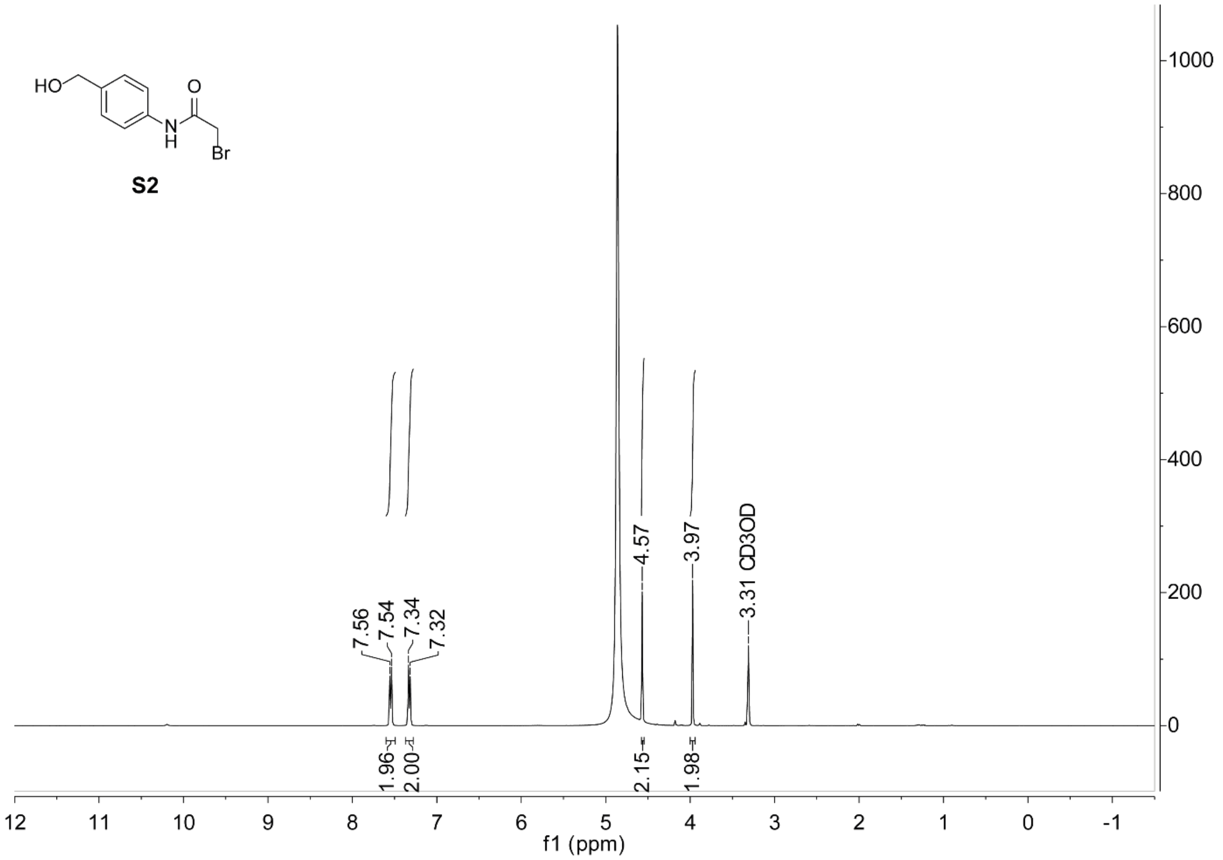

HO
O
N
H
Br
S2
7.56
7.54
7.34
7.32
4.57
3.97
3.31 CD3OD
1.96
2.00
2.15
1.98
f1 (ppm)

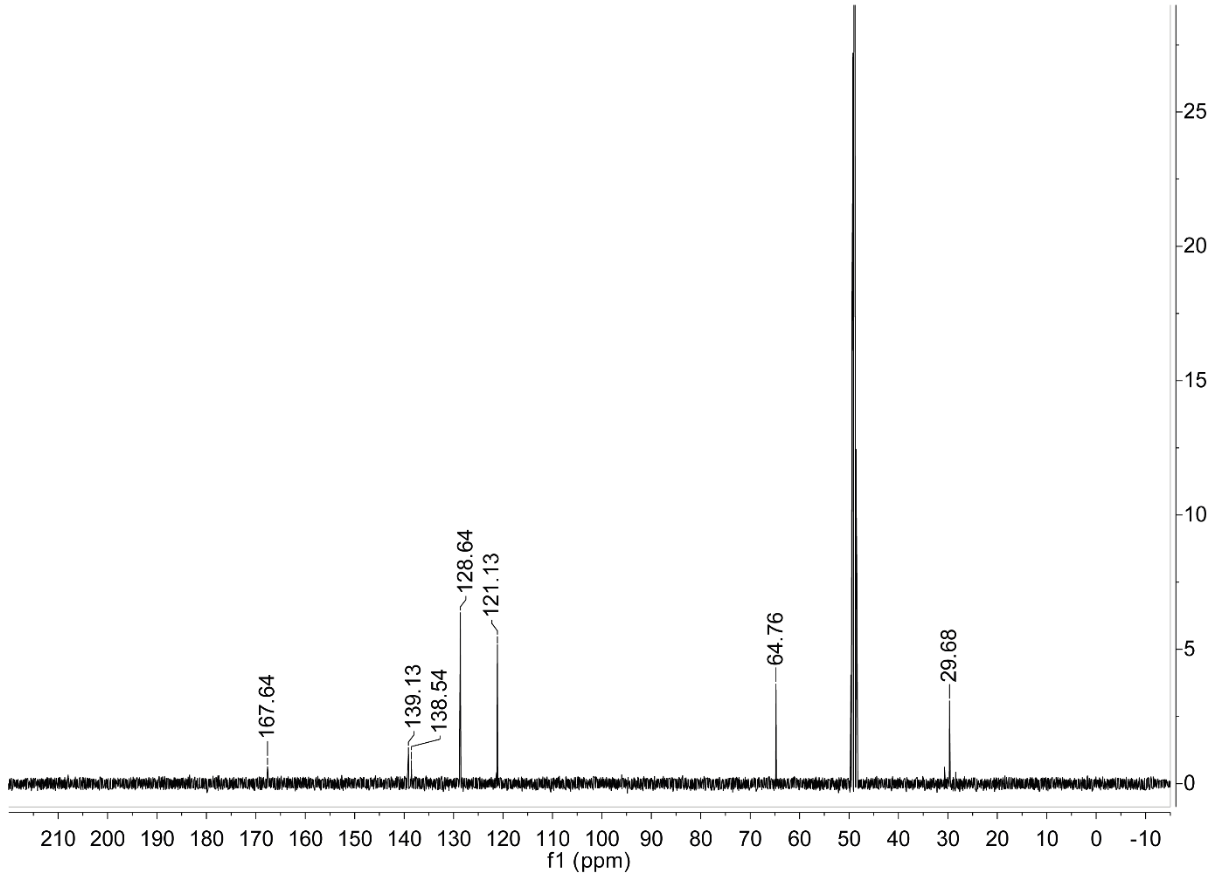

167.64
139.13
138.54
128.64
121.13
64.76
29.68
f1 (ppm)

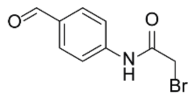
O
O
N
H
Br

S3

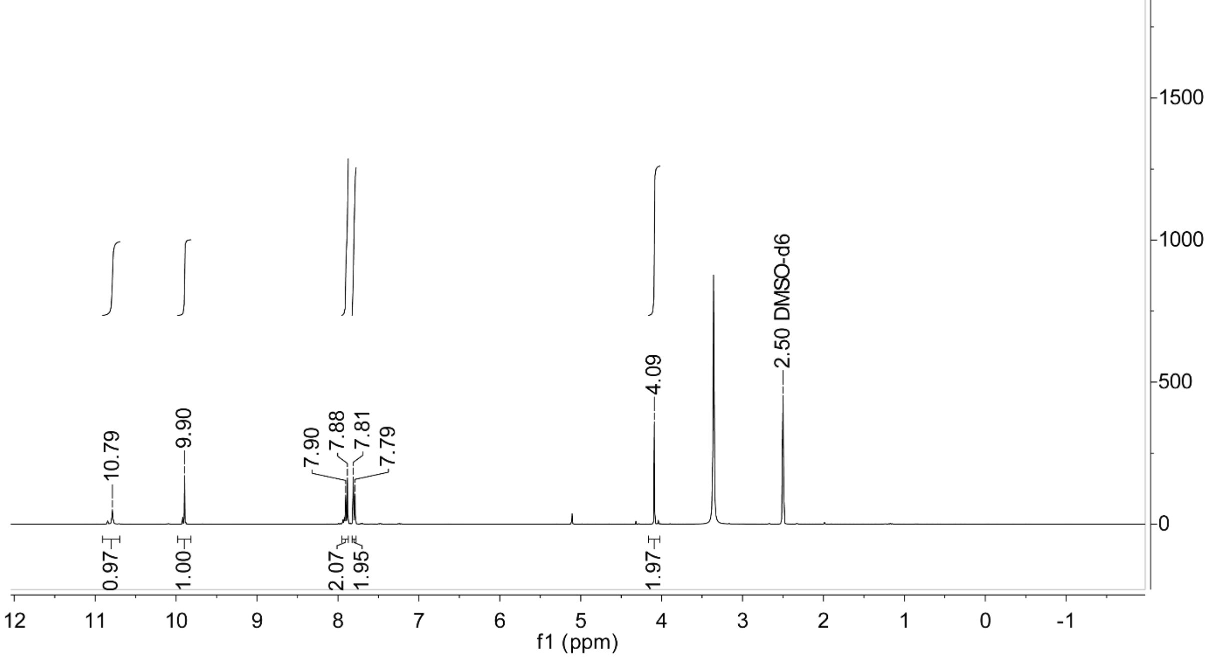
10.79
9.90
7.90
7.88
7.81
7.79
4.09
2.50 DMSO-d6
0.97
1.00
2.07
1.95
1.97
f1 (ppm)

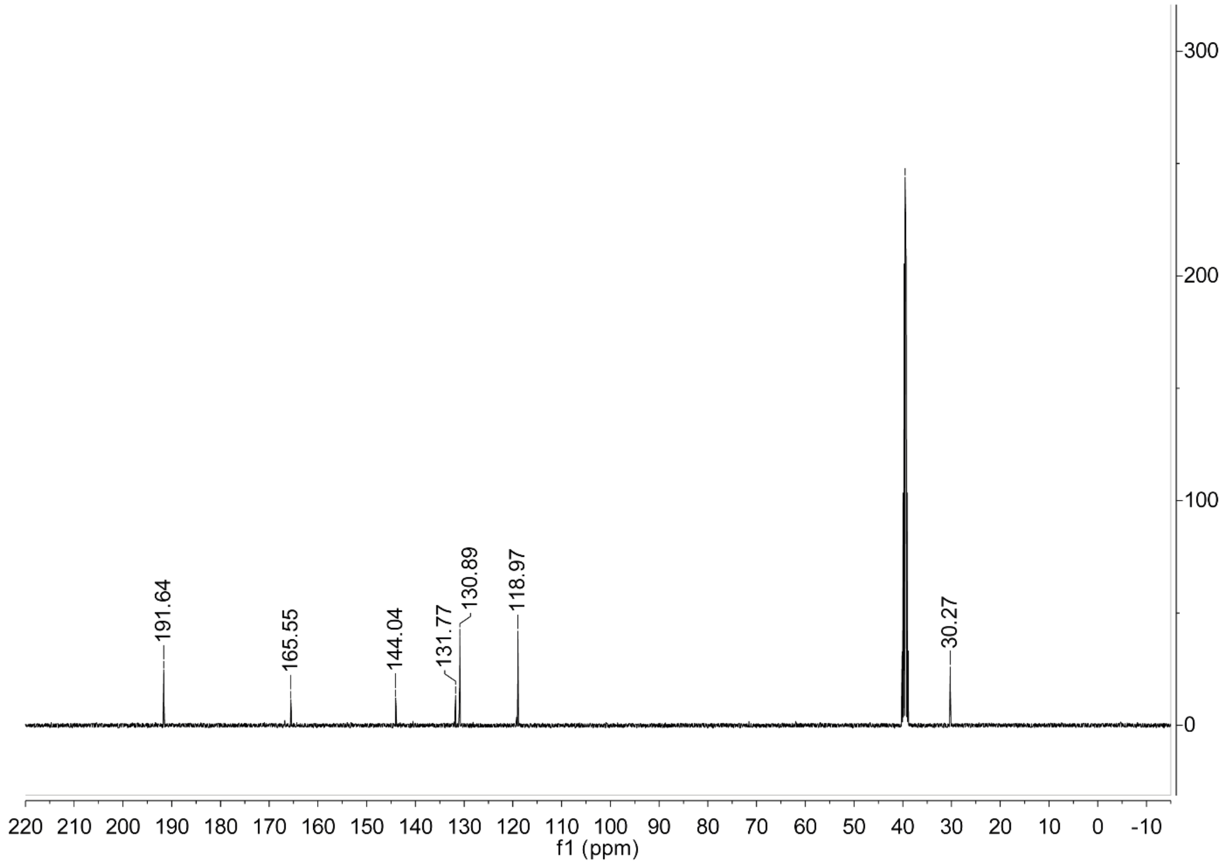
191.64
165.55
144.04
131.77
130.89
118.97
30.27
f1 (ppm)

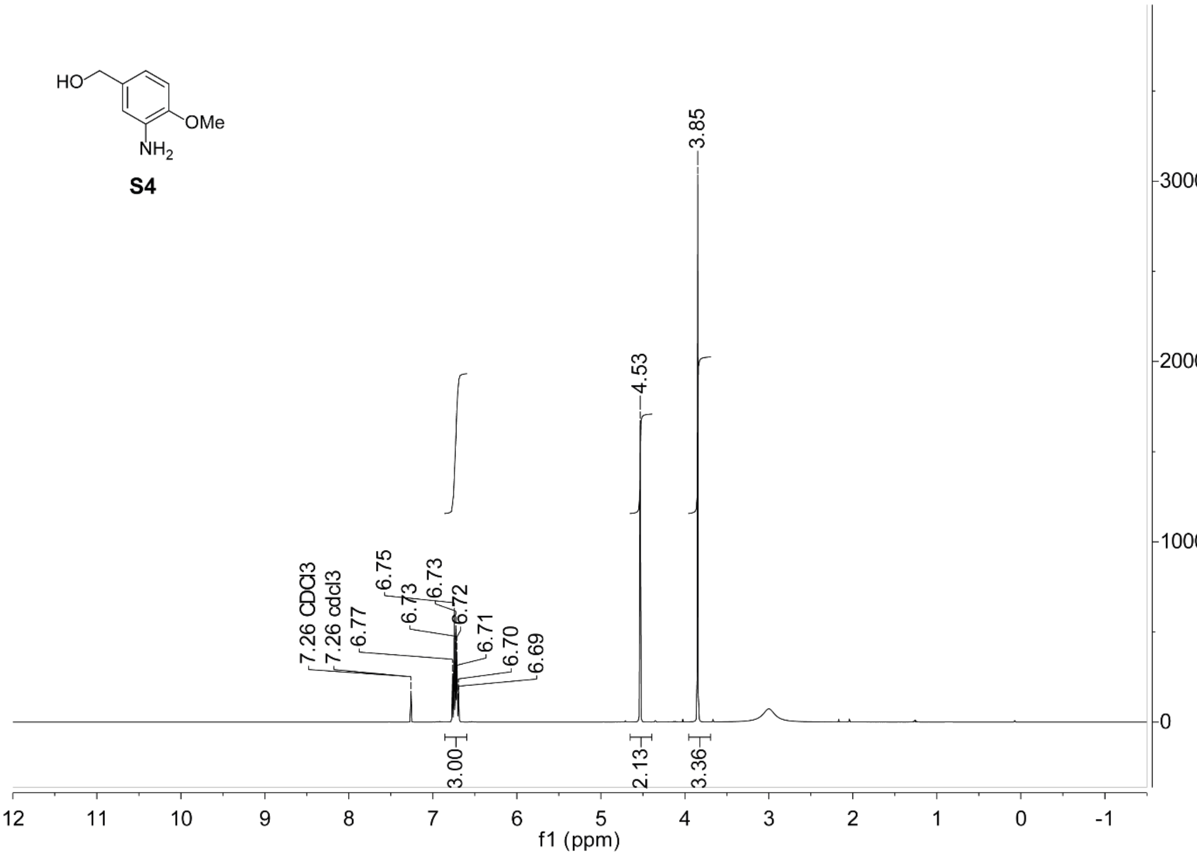

S4
3.85
4.53
7.26 CDCl3
7.26 cdcl3
6.77
6.75
6.73
6.73
6.72
6.71
6.70
6.69
3.00
2.13
3.36
f1 (ppm)

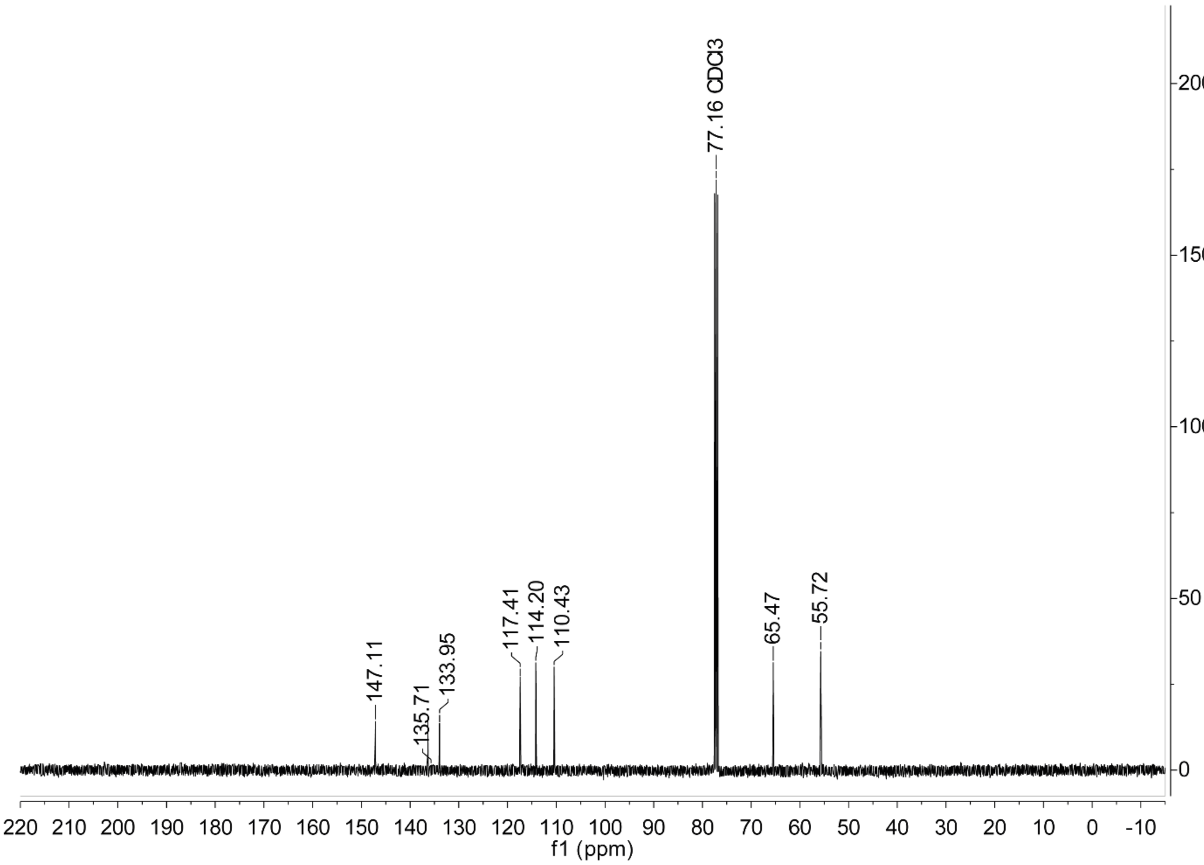

77.16 CDCl3
147.11
135.71
133.95
117.41
114.20
110.43
65.47
55.72
f1 (ppm)

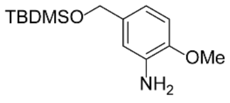

S5

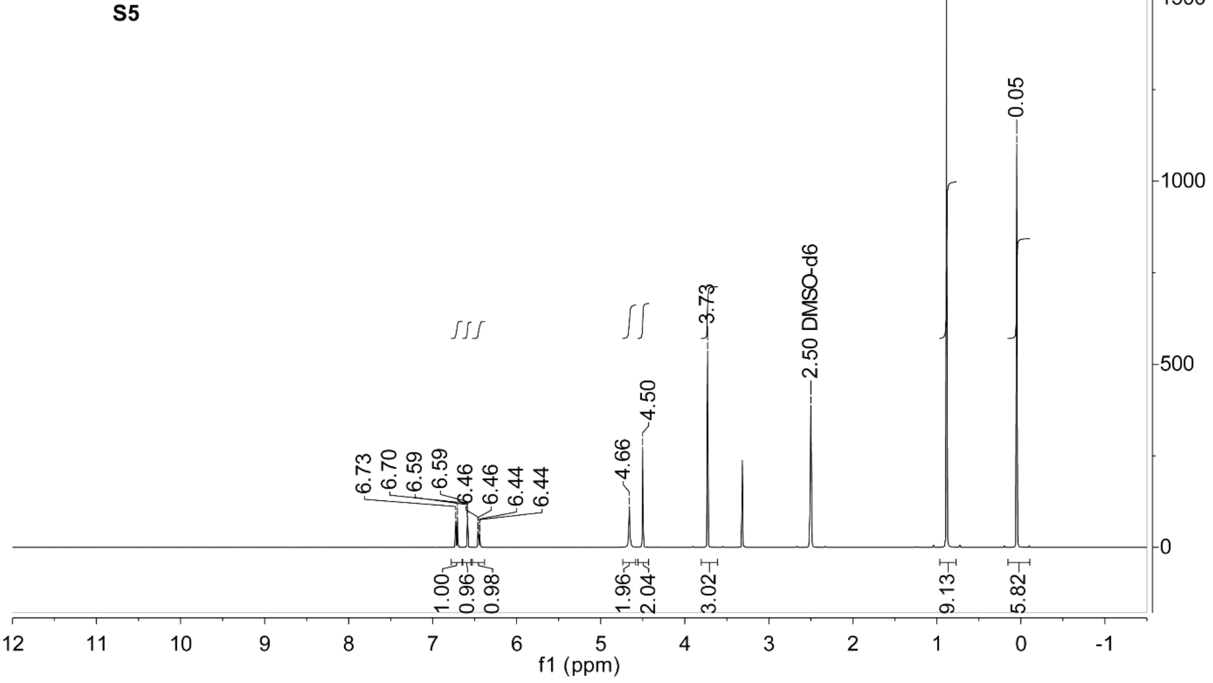

6.73
6.70
6.59
6.59
6.46
6.46
6.44
6.44
4.66
4.50
3.73
2.50 DMSO-d6
0.88
0.05
1.00
0.96
0.98
1.96
2.04
3.02
9.13
5.82
1500
1000
500
0
12 11 10 9 8 7 6 5 4 3 2 1 0 -1
f1 (ppm)

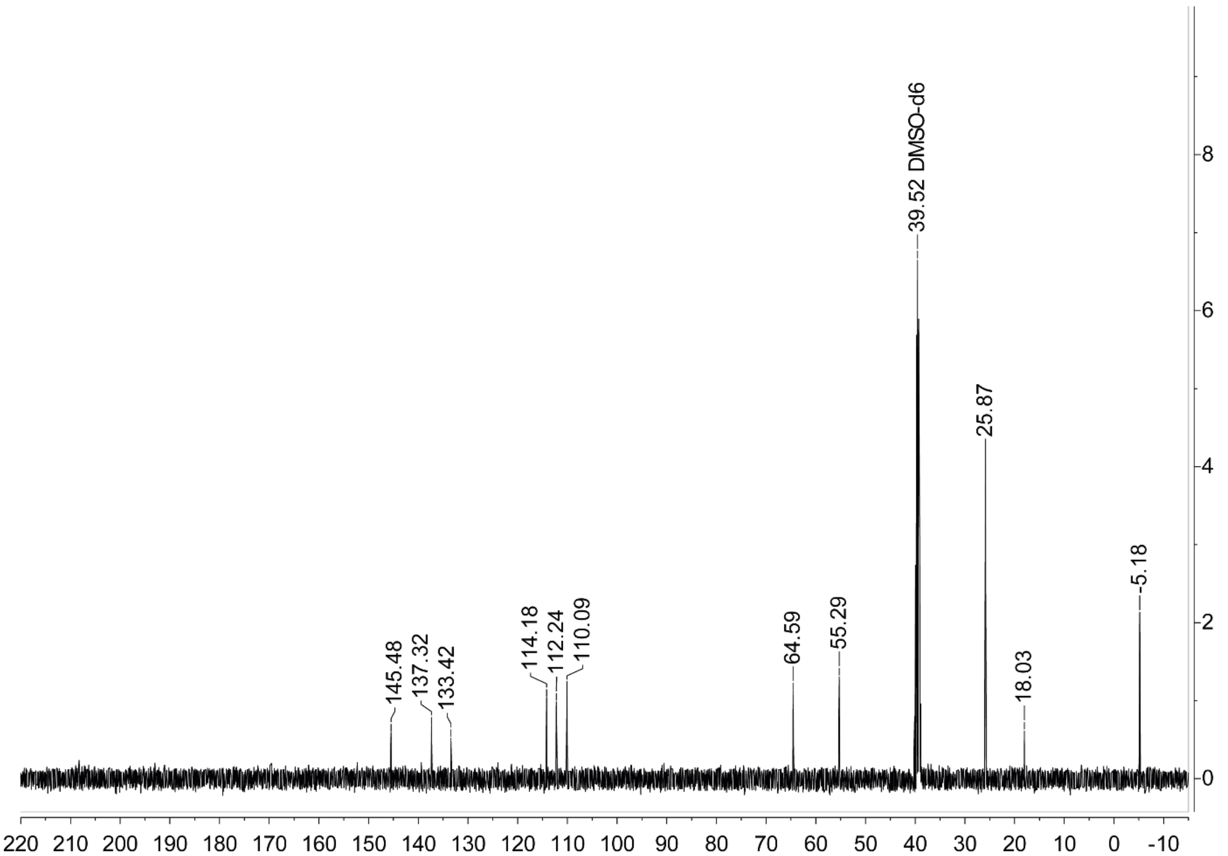

145.48
137.32
133.42
114.18
112.24
110.09
64.59
55.29
39.52 DMSO-d6
25.87
18.03
-5.18
8
6
4
2
0
220 210 200 190 180 170 160 150 140 130 120 110 100 90 80 70 60 50 40 30 20 10 0 -10
f1 (ppm)

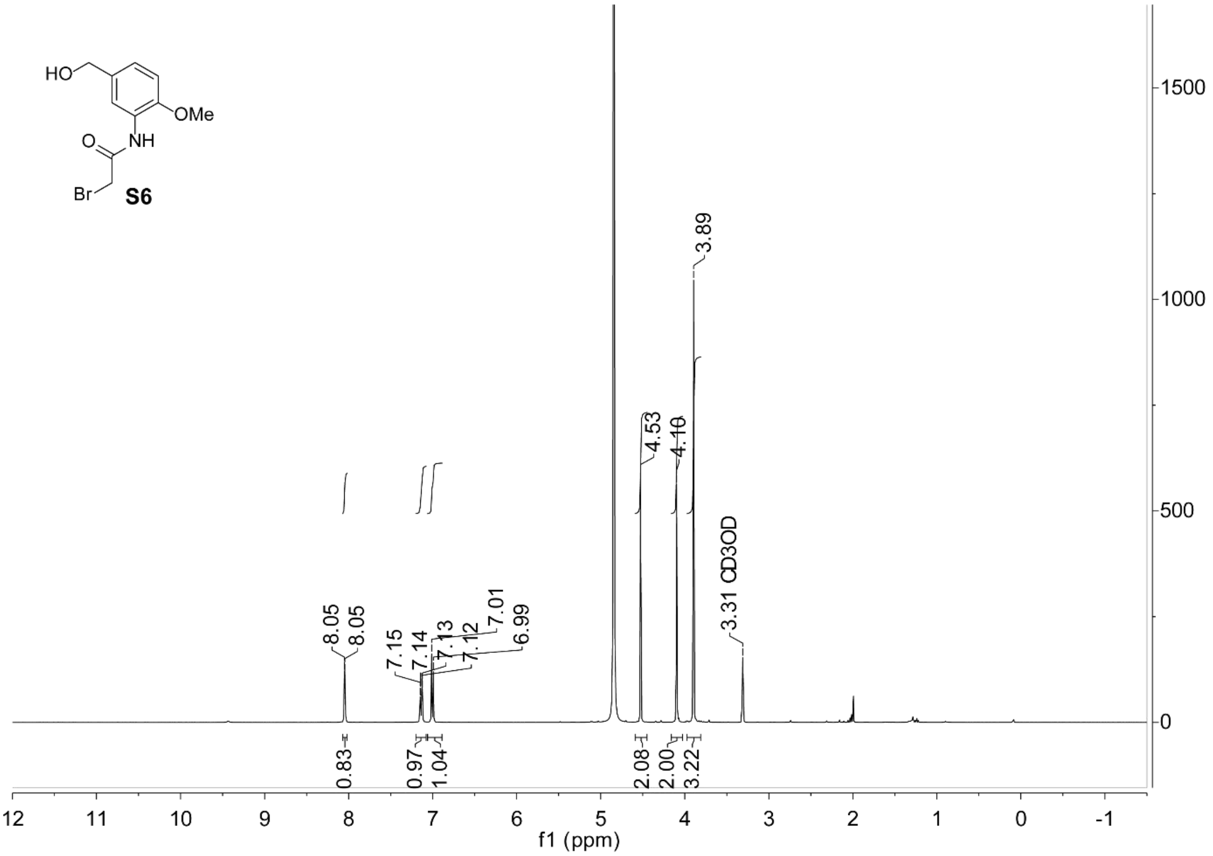
HO
OMe
O
NH
Br
S6
8.05
8.05
7.15
7.14
7.13
7.12
7.01
6.99
4.53
4.10
3.89
3.31 CD3OD
0.83
0.97
1.04
2.08
2.00
3.22
1500
1000
500
0
12
11
10
9
8
7
6
5
4
3
2
1
0
-1
f1 (ppm)

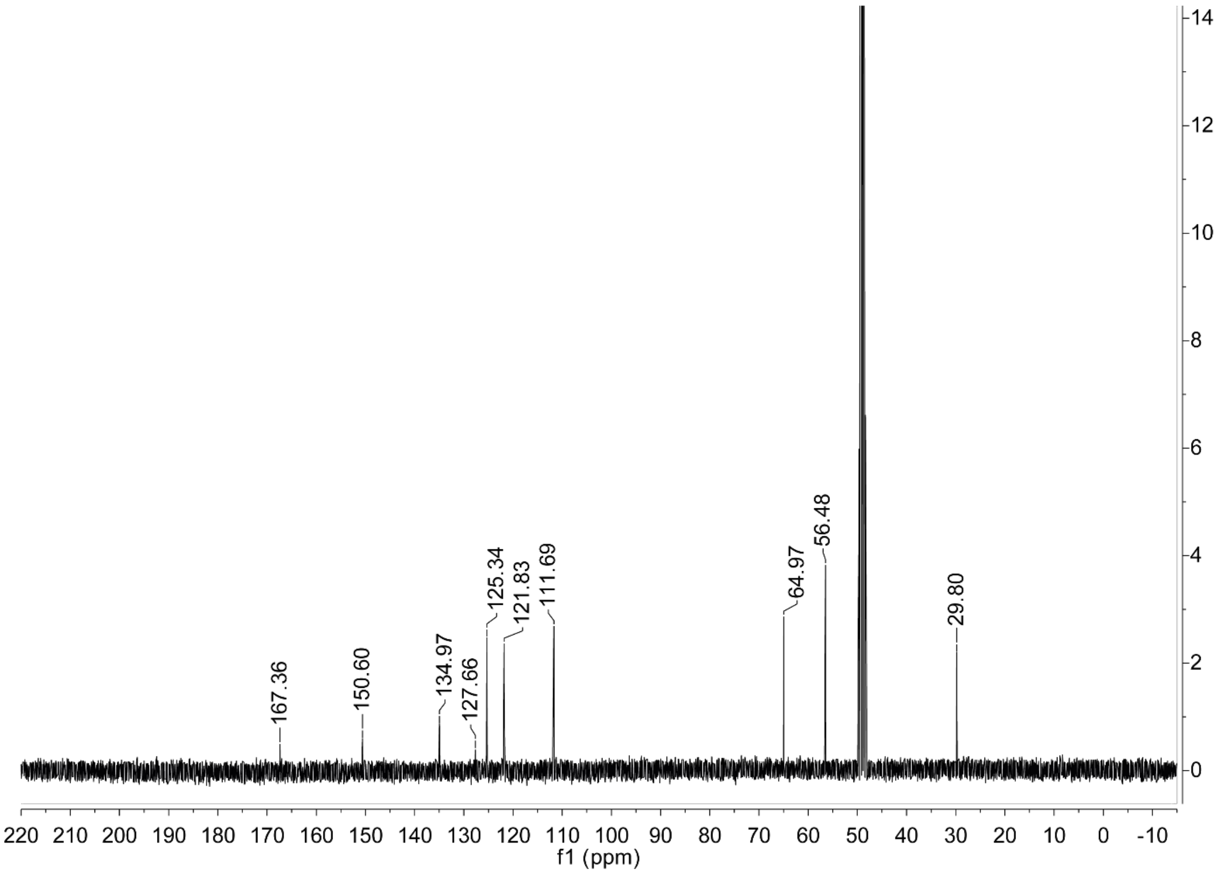
167.36
150.60
134.97
127.66
125.34
121.83
111.69
64.97
56.48
29.80
14
12
10
8
6
4
2
0
220 210 200 190 180 170 160 150 140 130 120 110 100 90 80 70 60 50 40 30 20 10 0 -10
f1 (ppm)

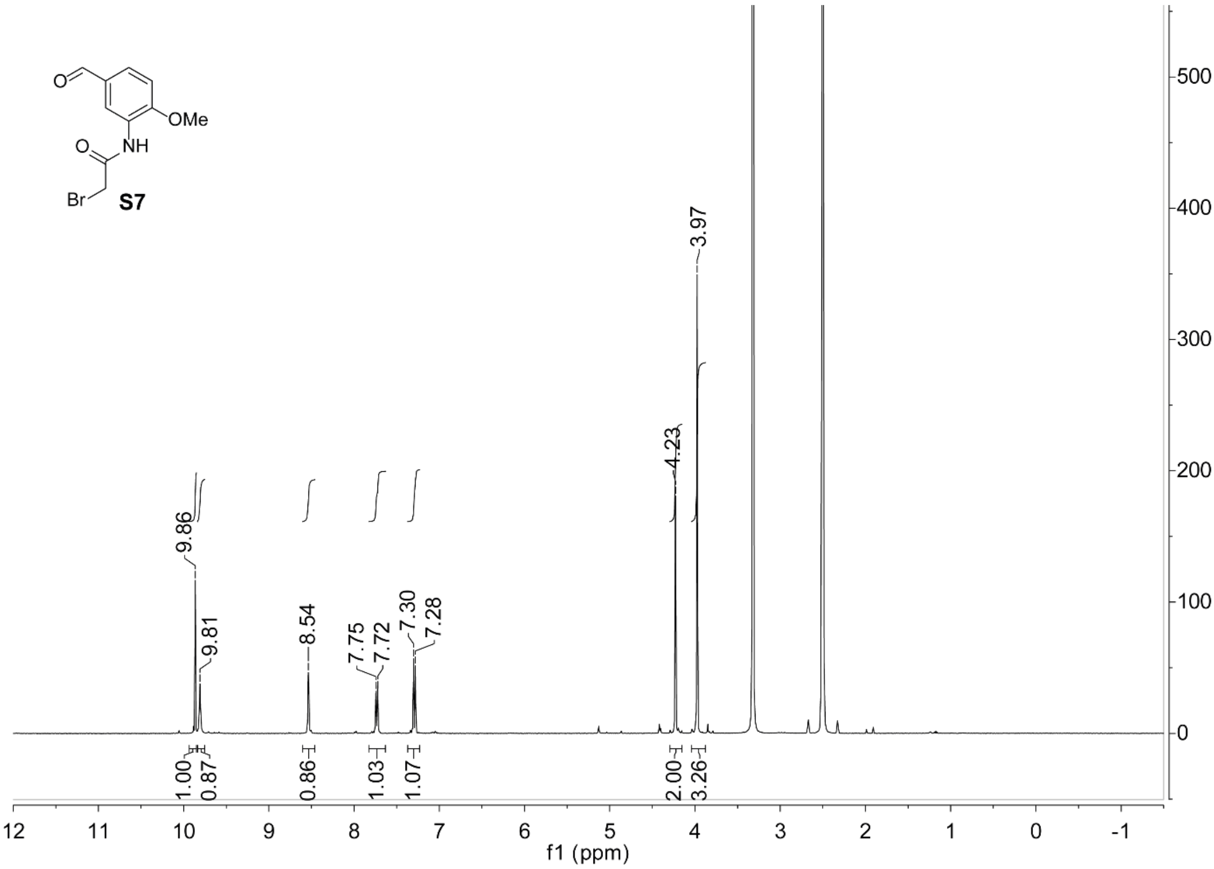
O
OMe
NH
O
Br
S7
9.86
9.81
8.54
7.75
7.72
7.30
7.28
4.23
3.97
1.00
0.87
0.86
1.03
1.07
2.00
3.26
f1 (ppm)

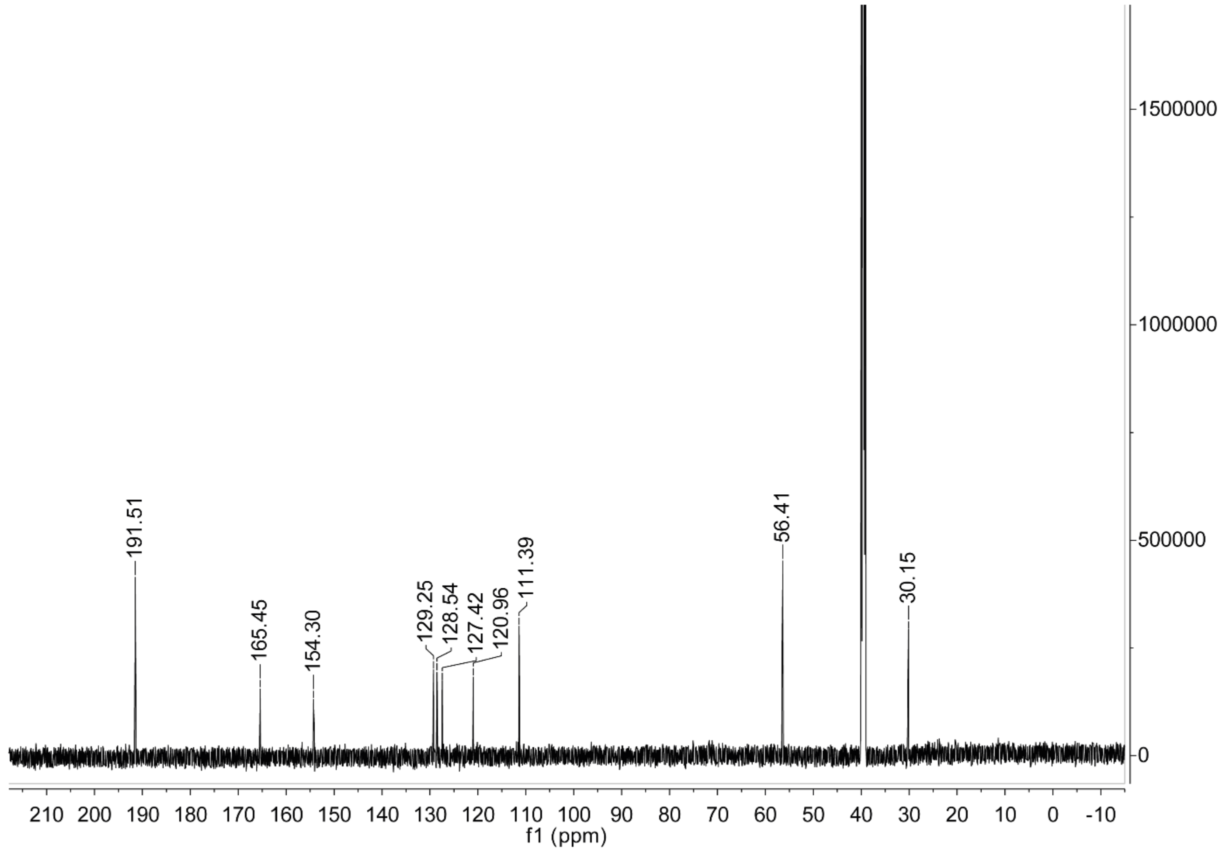
191.51
165.45
154.30
129.25
128.54
127.42
120.96
111.39
56.41
30.15
f1 (ppm)

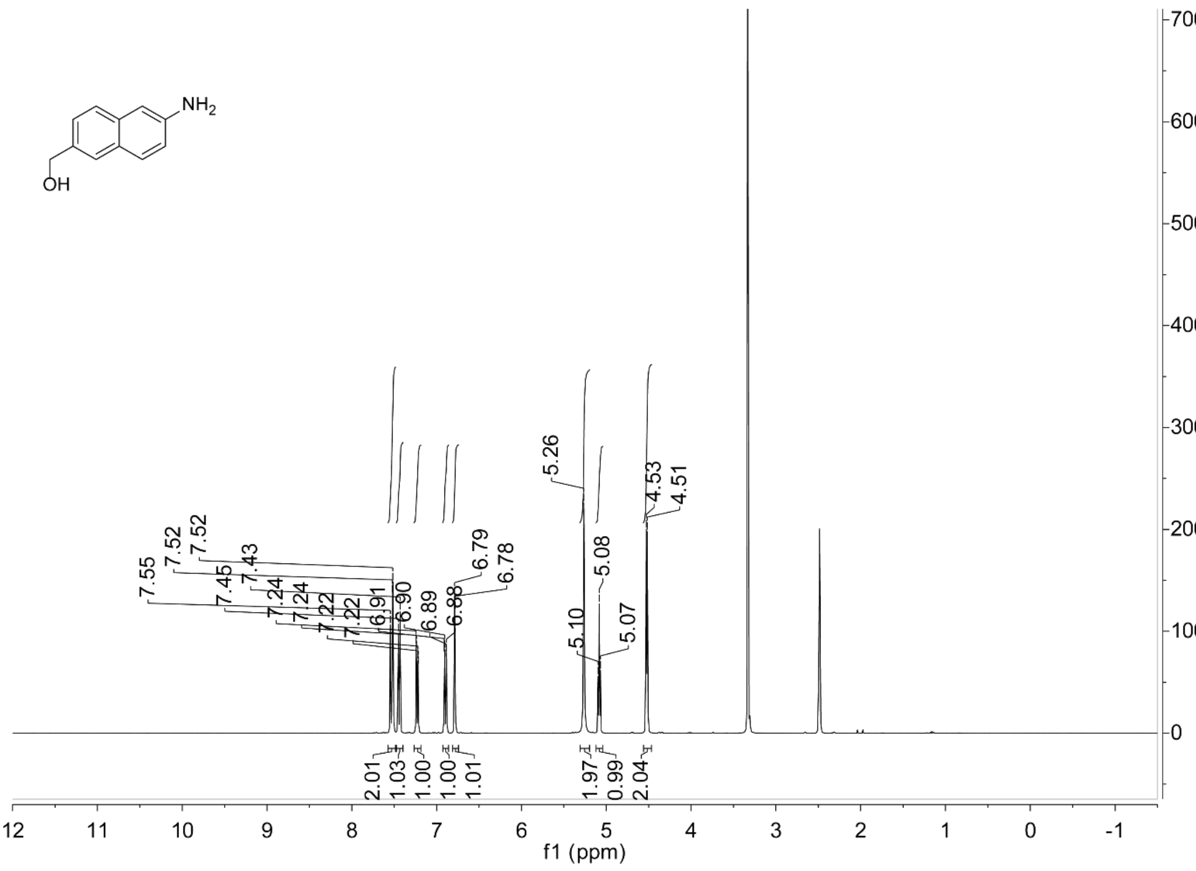
NH2
OH
7.55
7.52
7.52
7.45
7.43
7.24
7.24
7.22
7.22
6.91
6.90
6.89
6.88
6.79
6.78
5.26
5.10
5.08
5.07
4.53
4.51
2.01
1.03
1.00
1.00
1.01
1.97
0.99
2.04
f1 (ppm)

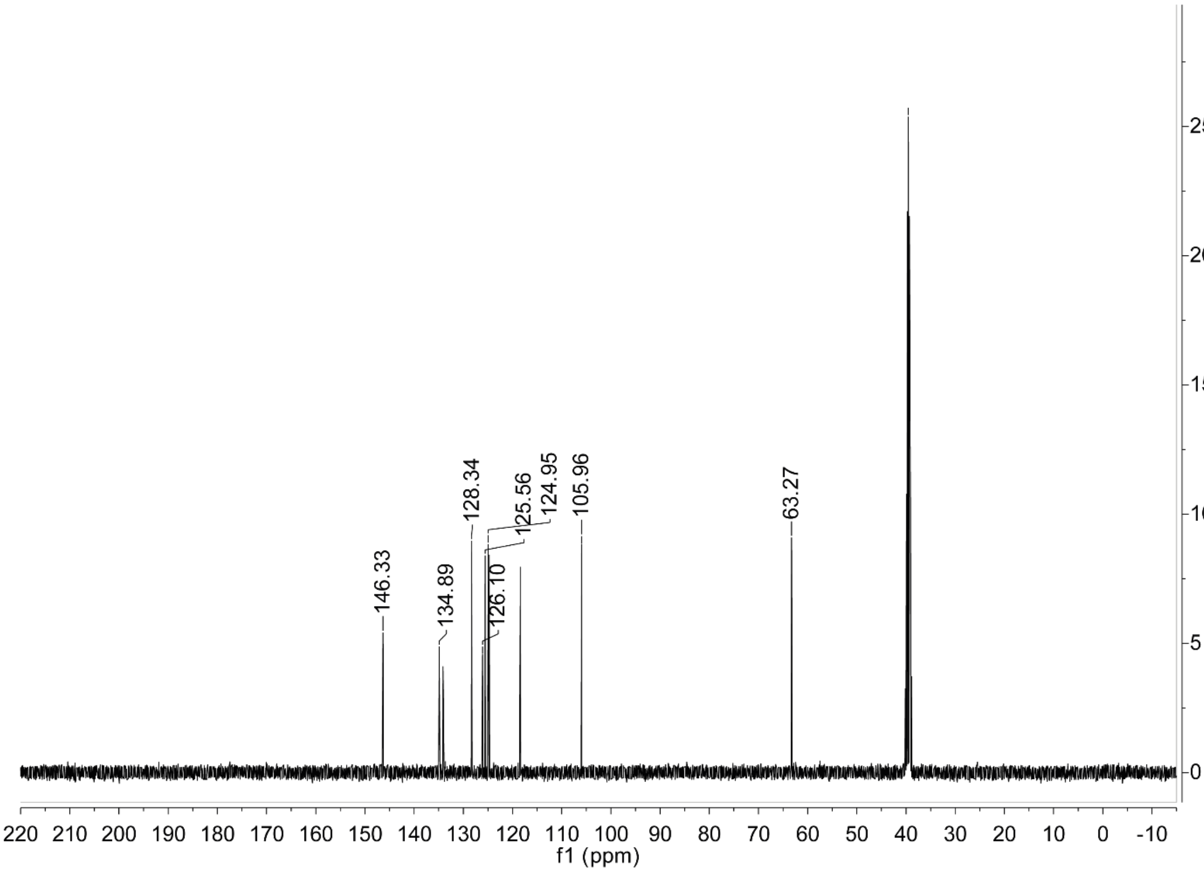
146.33
134.89
128.34
126.10
125.56
124.95
105.96
63.27
f1 (ppm)

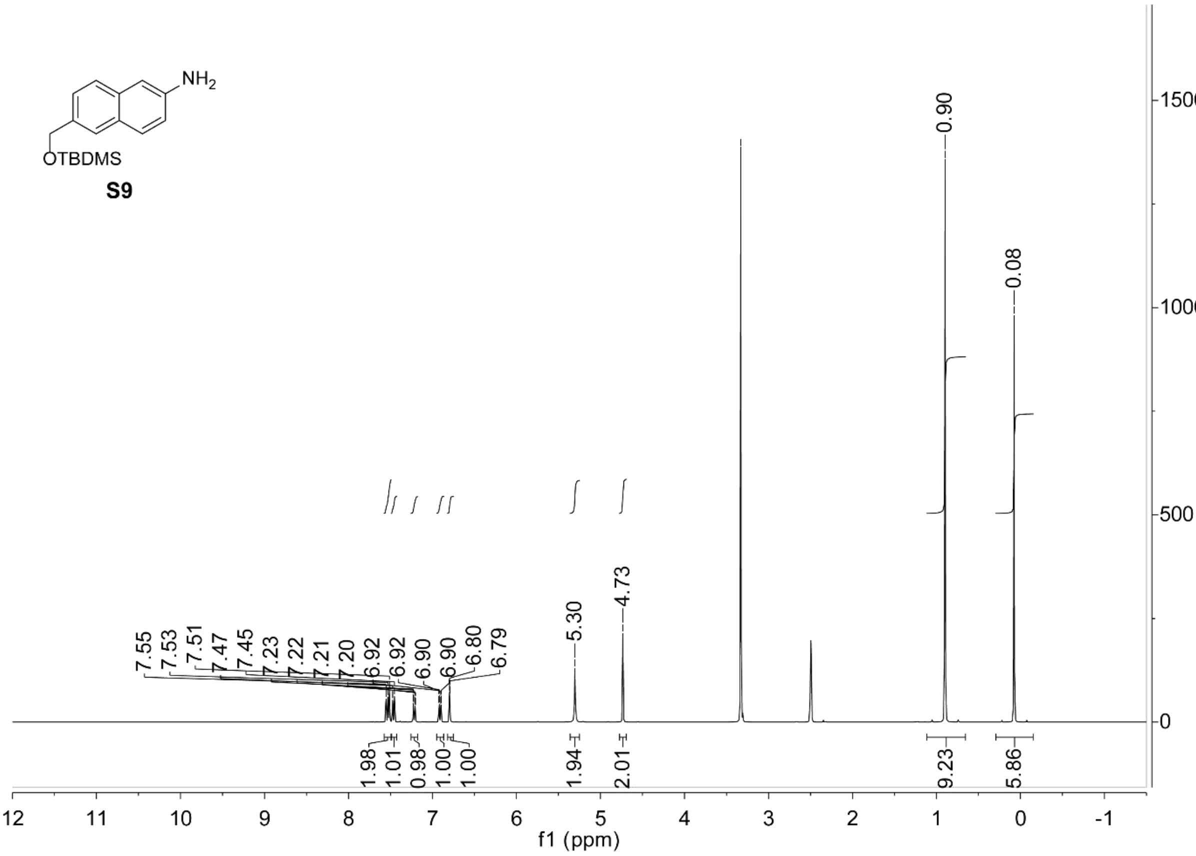
NH2
OTBDMS
7.55
7.53
7.51
7.47
7.45
7.23
7.22
7.21
7.20
6.92
6.92
6.90
6.90
6.80
6.79
5.30
4.73
0.90
0.08
1.98
1.01
0.98
1.00
1.00
1.94
2.01
9.23
5.86
1500
1000
500
0
12
11
10
9
8
7
6
5
4
3
2
1
0
-1
f1 (ppm)

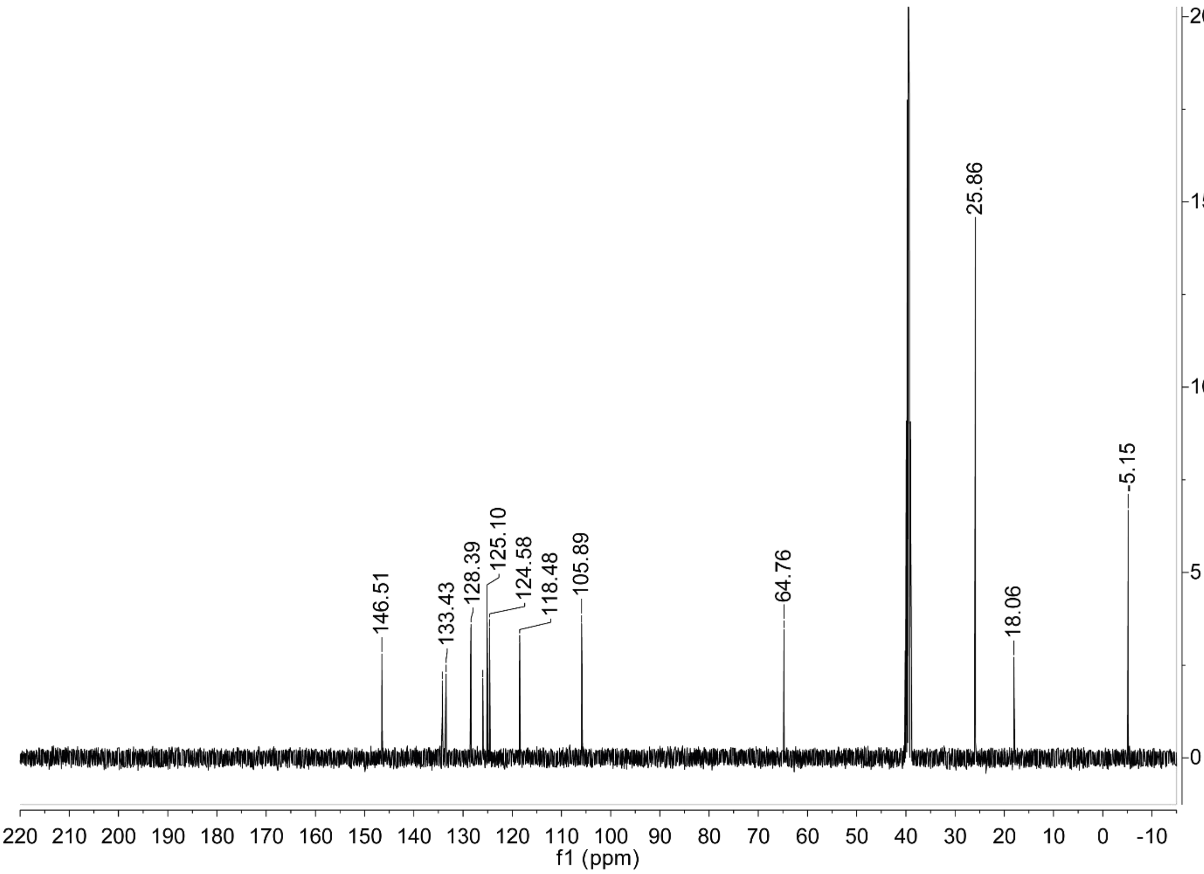
146.51
133.43
128.39
125.10
124.58
118.48
105.89
64.76
25.86
18.06
-5.15
20
15
10
5
0
220 210 200 190 180 170 160 150 140 130 120 110 100 90 80 70 60 50 40 30 20 10 0 -10
f1 (ppm)

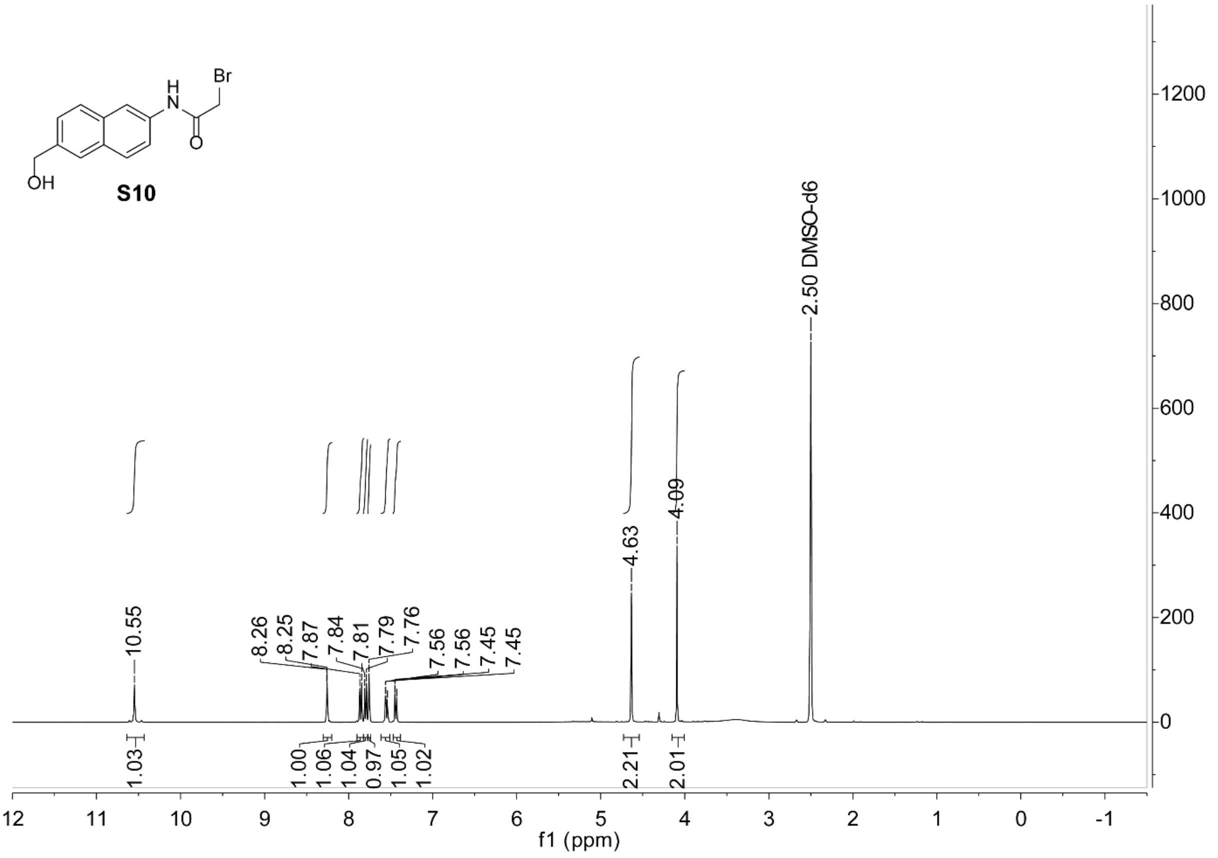

Br
H
N
O
OH
S10
2.50 DMSO-d6
4.09
4.63
10.55
8.26
8.25
7.87
7.84
7.81
7.79
7.76
7.56
7.56
7.45
7.45
1.03
1.00
1.06
1.04
0.97
1.05
1.02
2.21
2.01
f1 (ppm)

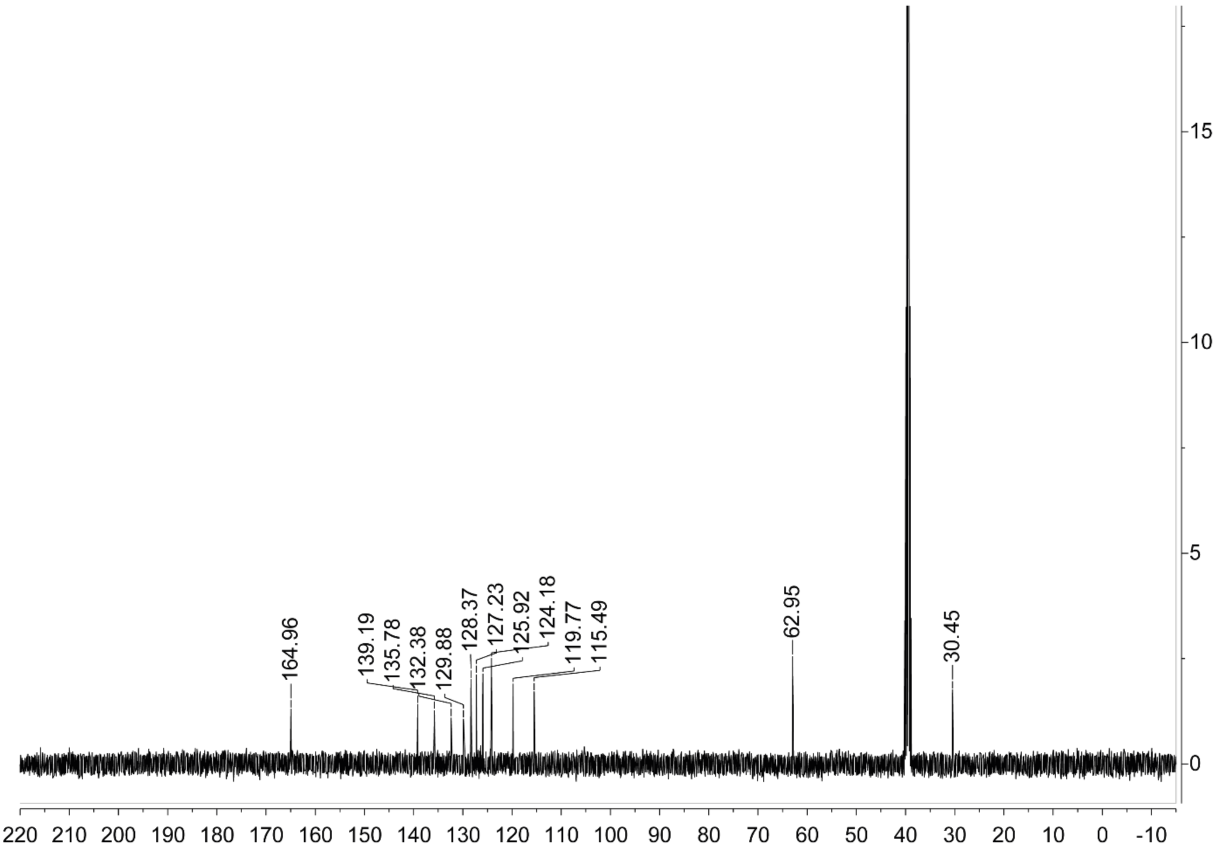

164.96
139.19
135.78
132.38
129.88
128.37
127.23
125.92
124.18
119.77
115.49
62.95
30.45
f1 (ppm)

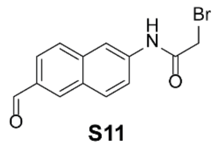
H
N
Br
O
O

S11

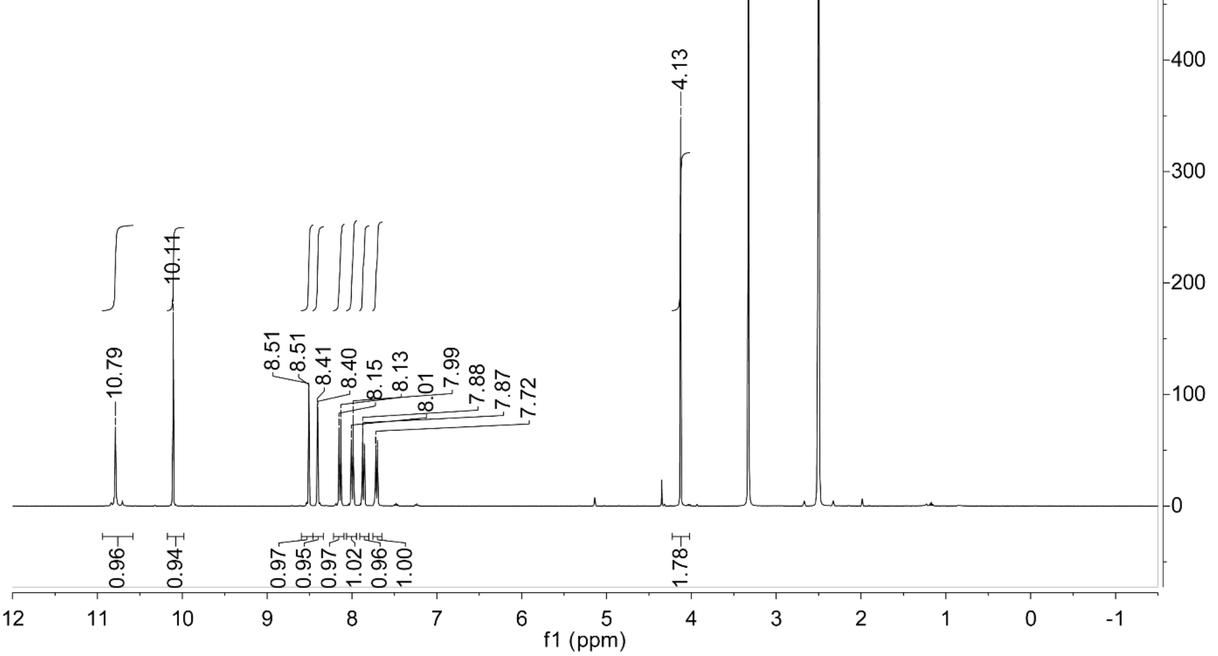
10.79
10.11
8.51
8.51
8.41
8.40
8.15
8.13
8.01
7.99
7.88
7.87
7.72
4.13
0.96
0.94
0.97
0.95
0.97
1.02
0.96
1.00
1.78
f1 (ppm)

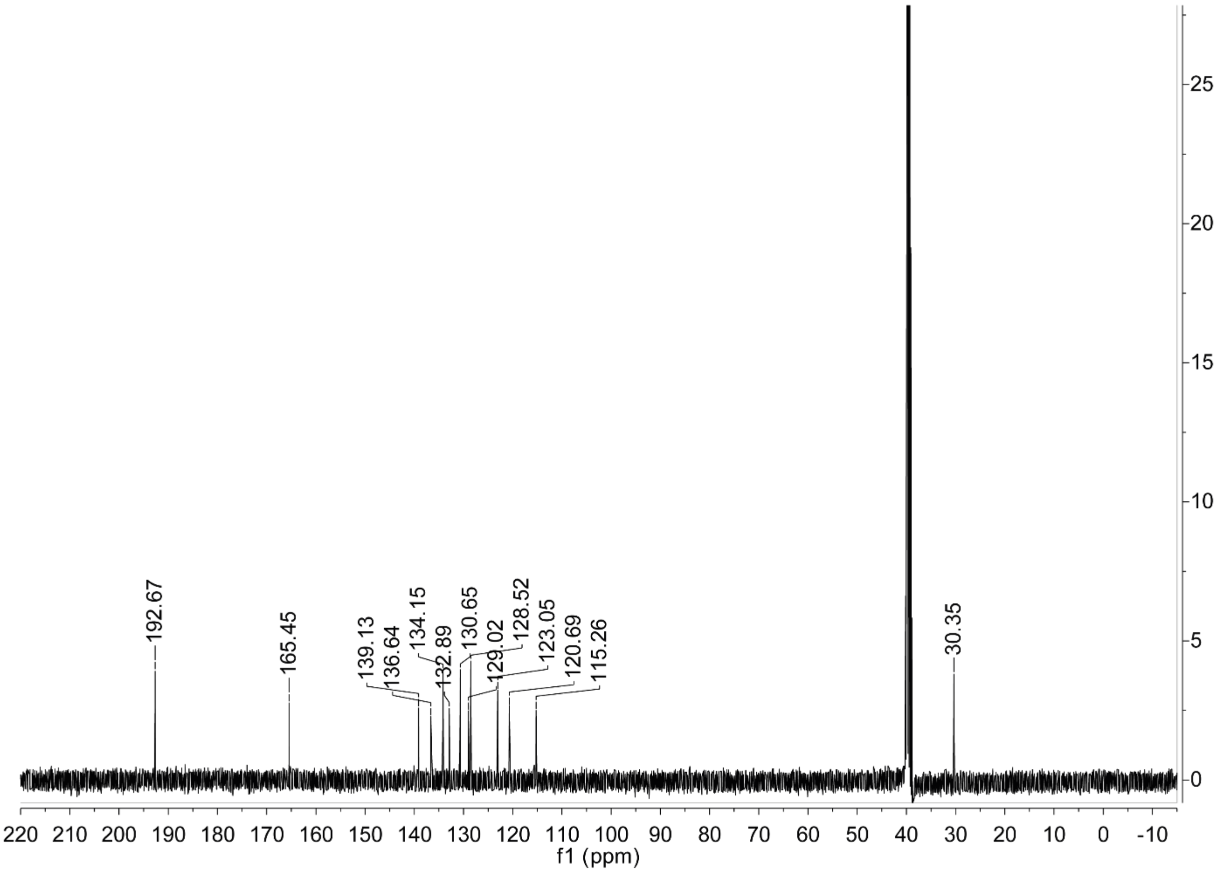
192.67
165.45
139.13
136.64
134.15
132.89
130.65
129.02
128.52
123.05
120.69
115.26
30.35
f1 (ppm)

16. REFERENCES

[1] Z. Zhang, A. G. Marshall, *J Am Soc Mass Spectrom* **1998**, *9*, 225-233.

[2] H. Xie, D. Ng, S. N. Savinov, B. Dey, P. D. Kwong, et al., *J. Med. Chem.* **2007**, *50*, 4898-4908.

[3] D. Kuwahara, T. Hasumi, H. Kaneko, M. Unno, D. Takahashi, et al., *Chem. Commun.* **2014**, *50*, 15601-15604.

[4] S. Sergeyev, M. Schar, P. Seiler, O. Lukoyanova, L. Echegoyen, et al., *Chem. Eur. J.* **2005**, *11*, 2284-2294.

[5] I. Kather, C. A. Bippes, F. X. Schmid, *J. Mol. Biol.* **2005**, *354*, 666-678.

[6] I.R. Rebello, C. Heinis, *Methods* **2013**, 60, 46-54.

[7] S.C. Meyer, T. Gaj, I.Ghosh, Chem. Biol. Drug Des. **2006**, 68, 3-10.

[8] N.M. Green, *Methods Enzymol.* **1990,** 184, 51-67.
